# Clog P‐Guided Development of Multi‐Colored Buffering Fluorescent Probes for Super‐Resolution Imaging of Lipid Droplet Dynamics

**DOI:** 10.1002/advs.202408030

**Published:** 2024-10-30

**Authors:** Jie Chen, Qinglong Qiao, Hanlixin Wang, Wenchao Jiang, Wenjuan Liu, Kai An, Zhaochao Xu

**Affiliations:** ^1^ Institution Dalian Institute of Chemical Physics Chinese Academy of Sciences 457 Zhongshan Road Dalian 116023 China; ^2^ University of Chinese Academy of Sciences Beijing 100049 China

**Keywords:** buffering fluorogenic probe, dynamics, lipid droplet, photostable, super‐resolution fluorescence imaging

## Abstract

Super‐resolution fluorescence imaging of live cells increasingly demands fluorescent probes capable of multi‐color and long‐term dynamic imaging. Understanding the mechanisms of probe‐target recognition is essential for the engineered development of such probes. In this study, it is discovered that the molecular lipid solubility parameter, Clog *P*, determines the staining performance of fluorescent dyes on lipid droplets (LDs). Fluorescent dyes with Clog *P* values between 2.5 and 4 can form buffering pools outside LDs, replacing photobleached dyes within LDs to maintain constant fluorescence intensity in LDs, thereby enabling dynamic super‐resolution imaging of LDs. Guided by Clog *P*, four different colored buffering LD probes spanning the visible light spectrum have been developed. Using Structured Illumination Microscopy (SIM), the role of LD dynamics have been tracked during cellular ferroptosis with the secretion, storage, and degradation of overexpressed ACSL3 proteins. It is found that LDs serve as storage sites for these proteins through membrane fusion, and further degrade overexpressed proteins via interactions with organelles like lysosomes or through lipophagy, thereby maintaining cellular homeostasis.

## Graphical abstract

Fluorescent dyes with Clog *P* values between 2.5 and 4 are identified to exhibit optimal buffering capabilities for LD staining. Using Clog *P* as a critical evaluation criterion, a range of fluorescent dyes are systematically screened and discovered four distinct‐colored BFPs for LDs, enabling multi‐target super‐resolution fluorescence imaging of dynamic interaction between LDs and other organelles.

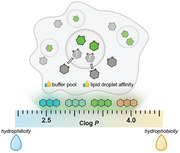

## 1 Introduction

Lipid droplets (LDs) are crucial organelles involved in lipid storage and energy metabolism within cells.^[^ ^1^ ^]^ They play pivotal roles in maintaining cellular homeostasis by dynamically responding to metabolic demands and environmental cues.^[^ ^2^ ^]^ The functions of LDs are diverse, encompassing lipid storage,^[^ ^3^ ^]^ signaling,^[^ ^4^ ^]^ and participation in various disease processes,^[^ ^5^ ^]^ highlighting their biological significance. However, studying the dynamics of LDs presents significant challenges, primarily due to their small size, rapid changes, heterogeneity, and unpredictability in cellular environments.^[^ ^6^ ^]^ Conventional imaging techniques often struggle to accurately capture these dynamics in real‐time due to the need to observe interactions at the nanoscale between LDs, and between LDs and other organelles, as well as the requirement for long‐term imaging to explore the complete and previously unknown dynamics of LDs.^[^ ^7^ ^]^

**Figure 1:**
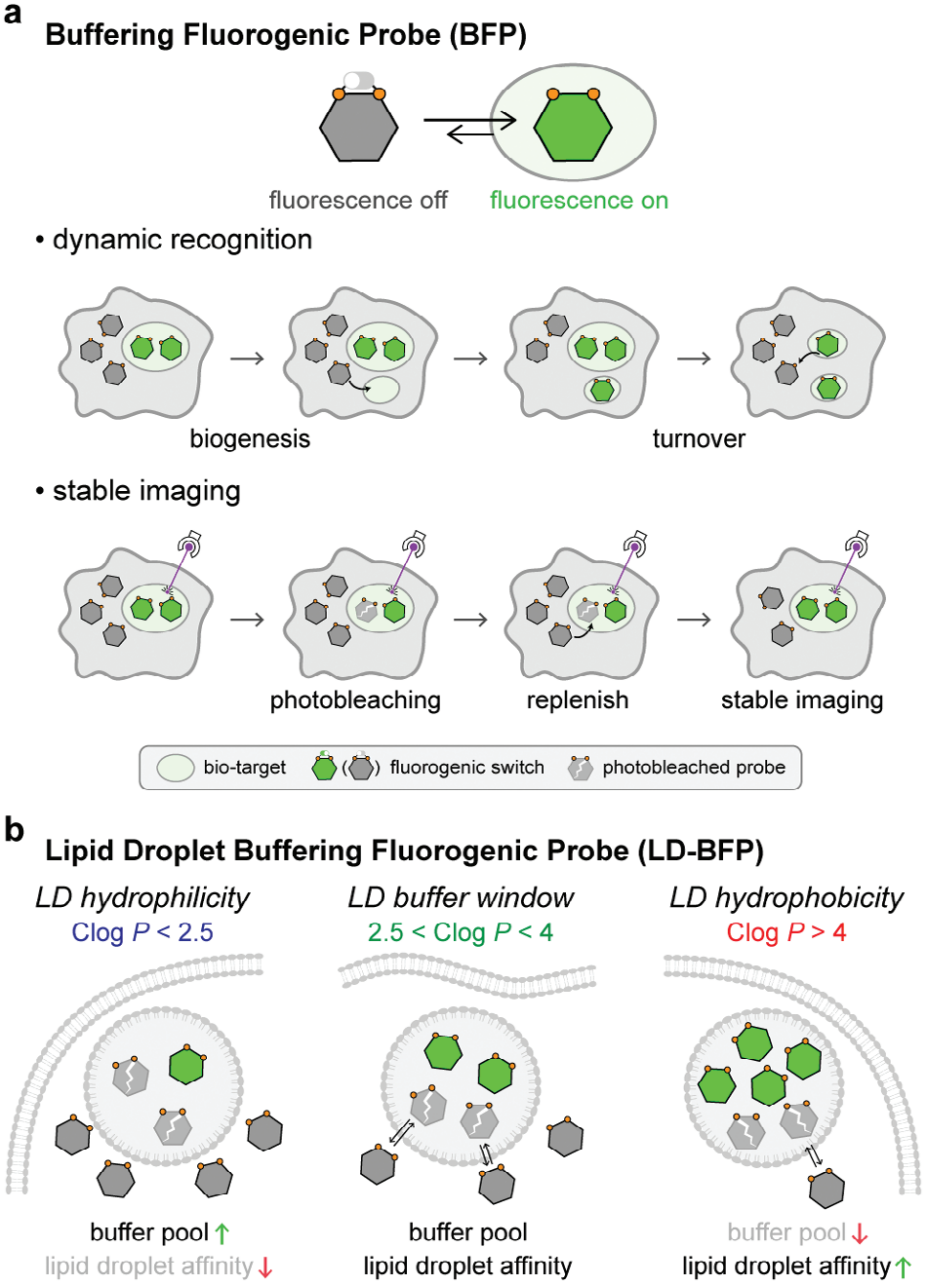
a) BFP as a general strategy for photostable dynamic imaging of bio‐targets. b) Rationally designing LD‐BFPs guided by Clog *P*.

To address these challenges, it is essential to develop specialized tools and techniques, particularly fluorescent probes that can specifically target LDs with high sensitivity, temporal and spatial resolution, and photostability to enable long‐term super‐resolution imaging.^[^ ^8^ ^]^ Photobleaching, which arises from the oxidation of fluorophores by reactive species, presents a persistent challenge for long‐term super‐resolution imaging of LDs, making it challenging to decipher nanoscale LDs dynamics. To address this, various buffering probes have been introduced to mitigate photobleaching issues in super‐resolution imaging. Once the probes within target structures undergo photobleaching, nearby fluorophores in the reservoir can efficiently replace the faded ones to ensure stable super‐resolution imaging over extended periods. In our previous work, we tackled the issues of dynamic labeling and photobleaching in LD Structured Illumination Microscopy (SIM) imaging by introducing a buffering fluorescent probe **LD‐FG**.^[^ ^9^ ^]^ The buffering probes replace photobleached molecules inside LDs with intact ones from outside LDs, thereby ensuring the long‐term stability of fluorescence imaging (**Figure** **1**a). This approach led to the discovery of various dynamic processes of LDs, including two novel modes of LD fusion and the heterogeneity in different cellular regions and between different cells.^[^ ^10^ ^]^

Building on this foundation, this study investigates the key factors controlling the buffering capability of LD probes, leading to the development of multi‐colored buffering fluorescent probes for LDs. By employing Clog *P*‐a parameter assessing lipid solubility‐we identified that fluorescent dyes with Clog *P* values between 2.5 and 4 exhibit optimal buffering capabilities for LD staining (Figure 1b).^[^ ^11^ ^]^ Furthermore, using Clog *P* as a critical evaluation criterion, we systematically screened a range of fluorescent dyes including naphthalimide, NBD, and BODIPY derivatives, leading to the discovery of four distinct‐colored buffering fluorescent probes for LDs, named **LD‐BFP405**, **LD‐BFP450**, **LD‐BFP488**, and **LD‐BFP543**. These probes have moderate lipid solubility, enabling wash‐free imaging of lipid droplets, and exhibit high photostability, making them suitable for multi‐target super‐resolution fluorescence imaging. Using SIM microscopy, we discovered that during ferroptosis, overexpressed ACSL3 proteins may be secreted into the cytoplasm via vesicle forms, with lipid droplets interacting with these vesicles through membrane fusion, serving as storage sites for the expressed proteins. Additionally, these proteins may further interact with organelles such as lysosomes or be degraded through lipophagy to maintain cellular homeostasis. These probes not only enhance our ability to visualize LD dynamics but also hold promise for advancing our understanding of LD biology in cellular processes and disease contexts.

## 2 Results and Discussion

### 2.1 Clog *P* Regulates the Buffering Capacity of LD‐BFPs

Lipid droplets (LDs) are structures composed of a monolayer of phospholipids surrounding a hydrophobic core of neutral lipids. Therefore, we hypothesize that the hydrophobicity of probes may be closely related to the buffering capacity of LD‐BFPs. Calculated log P (Clog *P*) is a quantitative descriptor used to assess lipophilicity, derived through substructure or whole molecule approaches, and widely employed in drug development and related fields. In this study, we introduced Clog *P* to investigate the relationship between the lipophilicity of LD‐BFPs and their buffering capacity, guiding the rational design of LD‐BFPs.

**Figure 2:**
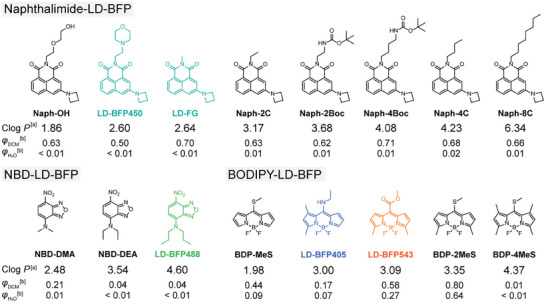
Chemical structures and physical properties of LD‐BFPs with various Clog *P* values. a) Clog *P* value was calculated by ChemDraw. b) Coumarin 153 was used to obtain relative fluorescence quantum yields (φ).

To investigate the relationship between Clog *P* and the buffer capacity of LD‐BFPs, we designed and synthesized a series of probes with varying Clog *P* values (**Figure** **2**). We introduced diverse hydrophilic and hydrophobic groups onto the naphthalimide scaffold to achieve Clog *P* values ranging from 1.86 to 6.34 for the Naphthalimide‐LD‐BFPs (Naph‐LD‐BFPs). This enabled us to explore how different Clog *P* values affect buffer capacity and identify an optimal range. Subsequently, we designed NBD‐LD‐BFPs and BODIPY‐LD‐BFPs (BDP‐LD‐BFPs) to evaluate whether this Clog *P* range could serve as a universal “LD buffer window”.

We initially characterized the fluorescence properties of these probes in vitro using dichloromethane (DCM) and water to simulate the low polarity, aprotic environment found in lipid droplets (LDs) and the high polarity, protic environment of the cytoplasm, respectively. As depicted in Figure 2 and Figures S1–S3 (Supporting Information), and Tables S1 and S2 (Supporting Information), probes sharing the same scaffold exhibited highly similar photophysical properties, demonstrating significant environmental sensitivity. The maximum emission wavelength (*λ_em_*) of Naphthalimide‐LD‐BFPs displayed a marked red shift from DCM to water (*λ_em_* ≈550 nm vs ≈640 nm), accompanied by a substantial decrease in fluorescence quantum yield (*φ_DCM_* ≈0.65 vs *φ_H2O_* ≈0.01). In our prior research, we proposed that upon photoexcitation, hydrogen bond (HB) accepting sites (oxygen atoms) undergo significant changes in charge density, leading to increased HB vibrations and efficient fluorescence quenching in protic solvents.

Similarly, NBD‐LD‐BFPs exhibited a comparable trend, with *λ_em_* also shifting ≈40 nm from DCM to water *(λ_em_* ≈530 nm vs *λ_em_* ≈570 nm), and a dramatic decrease in fluorescence quantum yield (*φ_DCM_* ≈0.20 vs *φ_H2O_* ≈0.01). We hypothesize that fluorescence quenching in water occurs due to two main reasons. First, analogous to 3‐azetidinyl‐substituted naphthalimides, the charge density of nitro‐ and benzofuroxan groups increases substantially post‐photoexcitation, leading to intense HB interactions with protic solvents. Second, the *N*,*N*‐dialkyl groups at the 7‐position facilitate the fluorophore's transition into the twisted intramolecular charge transfer (TICT) state after photoexcitation, further quenching fluorescence.^[^ ^12^ ^]^

For BODIPY‐LD‐BFPs, the fluorescence quantum yields of these probes also significantly decreased from DCM to water. Previous studies have suggested that twisted intramolecular charge shuttle (TICS), involving charge shuttle processes accompanied by the rotation of groups at the meso‐position, could be a plausible mechanism for fluorescence quenching.^[^ ^13^ ^]^ These findings collectively highlight the excellent fluorogenic properties of these LD‐BFPs, rendering them promising for imaging lipid droplets in living cells.

Subsequently, we selected Naphthalimide‐LD‐BFPs as model compounds to investigate the impact of Clog *P* on lipid droplet (LD) selectivity in live cells. As depicted in Figure S4 (Supporting Information), **Naph‐OH** (Clog *P* = 1.86), possessing relatively low lipophilicity, exhibited weak fluorescence in LDs and strong background fluorescence in the cytoplasm after 1 h of incubation with living cells. In contrast, other probes demonstrated robust and selective imaging of LDs in living cells, as validated by colocalization experiments with commercial LD probes (Figure S5, Supporting Information). Based on these observations, we propose that Naphthalimide‐LD‐BFPs with Clog *P* < 2.5 exhibit “LD hydrophilicity” and do not efficiently target LDs. Conversely, Naphthalimide‐LD‐BFPs with Clog *P* > 2.5 demonstrate effective LD imaging capabilities with high selectivity in living cells.

Next, we explored the relationship between the Clog *P* value and the buffering capacity of naphthalimide‐LD‐BFPs. After incubating living HeLa cells with the probes for 30 min, we subjected a small area to higher laser intensity for 10 s to induce rapid photobleaching of the probe. Subsequently, we switched to a milder laser intensity to minimize further photobleaching and monitored the fluorescence changes. During photobleaching, the photoexcited fluorophores may undergo oxidation, increasing their hydrophilicity and transient accumulation in LDs. This accelerated the exchange between photobleached fluorophores and intact probes in the buffer pool within LDs and the cytoplasm, resulting in fluorescence recovery. We termed this phenomenon “Buffering Fluorescence Recovery after Photobleaching” (BuFRAP). We hypothesized that LD‐BFPs with the same fluorophore exhibit similar fluorescence bleaching rates, allowing us to assess the buffer capacity of LD‐BFPs based on the rate of fluorescence recovery (*k*_t_) and the extent of final fluorescence recovery.

**Figure 3:**
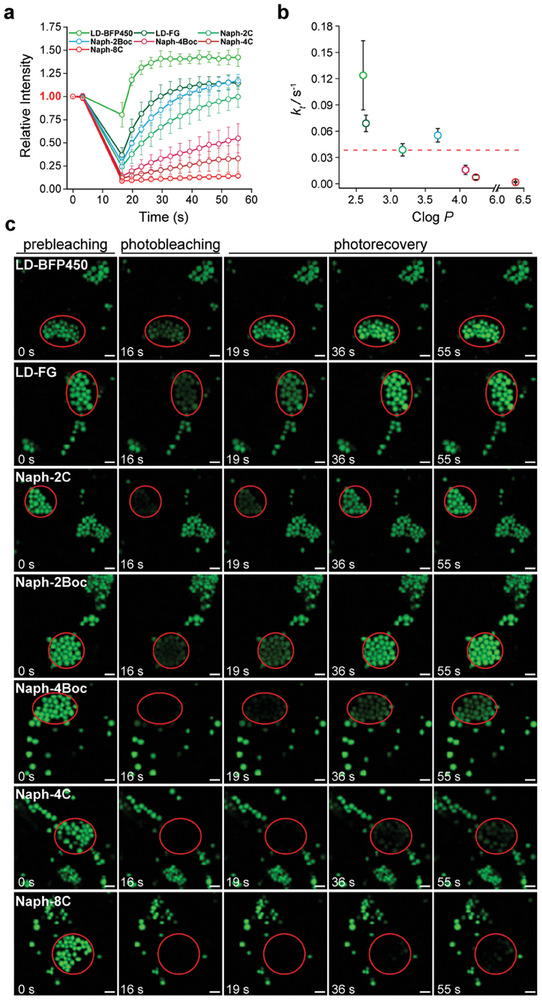
BuFRAP experiments of Naph‐LD‐BFPs in living HeLa cells. a) The relative intensity of the bleaching area during photobleaching and photorecovery processes of Naph‐LD‐BFPs. The ± s.d. from at least 5 BuFRAP experiments each. b) The rate of fluorescence recovery (*k*_t_) of Naph‐LD‐BFPs with various Clog *P* values. c) Confocal images of 2 µM Naph‐LD‐BFPs in living HeLa cells during photobleaching and photorecovery processes. Red circles highlighted the bleaching area. Scale bar = 2 µm.

We conducted BuFRAP experiments on Naphthalimide‐LD‐BFPs to investigate their buffering capacity, as depicted in **Figure** **3**a, revealing a strong correlation with Clog *P*. Probes with moderate lipophilicity (2.5 < Clog *P* < 4) exhibited high photostability, particularly **LD‐BFP450** (Clog *P* = 2.60). When subjected to continuous laser irradiation in a selected area, **LD‐BFP450**’s fluorescence intensity decreased by only ≈20% and rapidly recovered to ≈125% of the initial fluorescence intensity (F_0_) within 3 s. Clear LD images were observed in the second frame after bleaching and remained stable thereafter (Figure 3c). The minimal photobleaching and rapid photorecovery were attributed to the suitable affinity of **LD‐BFP450** for LDs and the larger buffer pool in the cytoplasm, controlled by its Clog *P* value. Interestingly, the fluorescence intensity of **LD‐BFP450** after strong laser bleaching exceeded F_0_, suggesting that the local reduction of phospholipids on LD membranes or stress response due to continuous laser irradiation allowed more buffer probes to enter LDs.

**LD‐FG** (Clog *P* = 2.64) exhibited the second‐best fluorescence recovery ability. Its fluorescence intensity decreased to ≈36% of F_0_ after photobleaching, recovered rapidly to F_0_ within 10 s, and finally stabilized at ≈115% of F_0_. Although there was a slight difference between **Naph‐2C** (Clog *P* = 3.17) and **Naph‐2Boc** (Clog *P* = 3.68), both probes fully recovered to F_0_ within 1 min. In contrast, **Naph‐4Boc** (Clog *P* = 4.08), **Naph‐4C** (Clog *P* = 4.23), and **Naph‐8C** (Clog *P* = 6.34) exhibited dramatic declines in fluorescence intensity after photobleaching, recovering only to 54, 33, and 14% of F_0_, respectively. Figure 3c also illustrated that the brightness of LDs in the bleached area was significantly darker than that in the unbleached area after 1 min.

Furthermore, we observed a notable linear relationship between *k*_t_ and imaging time during the early stages of BuFRAP. As depicted in Figure 3b, *k*_t_ exhibited a distinct negative correlation with Clog *P*, making it a straightforward indicator of the buffer capacity of Naphthalimide‐LD‐BFPs. Probes within the “LD buffer window” demonstrated high *k*_t_ values (*k*_t_ > 0.04), with **LD‐BFP450** (*k*_t_ = 0.124 ± 0.039 s^−1^) exhibiting the most robust resistance to photobleaching. In contrast, Naphthalimide‐LD‐BFPs with Clog *P* > 4 generally displayed *k*_t_ values less than 0.02 s^−1^, indicating poor buffer capacity and slow recovery after photobleaching. These findings underscore that LD‐BFPs with moderate lipophilicity enable effective loading of buffer probes within LDs while maintaining good buffering capacity outside LDs.

To investigate the direct influence of buffer probe quantity on the photorecovery process, we conducted BuFRAP experiments using **Naph‐4Boc**, which exhibits a “medium buffer capacity,” at various staining concentrations. As illustrated in Figure S6 (Supporting Information), increasing the staining concentration from 0.5 to 4 µM resulted in a corresponding increase in the photorecovery degree from ≈45 to ≈60%, accompanied by an increase in kt from 0.0126 ± 0.0019 s^−1^ to 0.0188 ± 0.0063 s^−1^. We propose that higher staining concentrations enhance the number of buffer probes available outside LDs, thereby accelerating the replenishment rate and enhancing the photorecovery degree. These findings underscore that the lipophilicity range of 2.5 < Clog *P* < 4 represents an optimal “LD buffer window” for Naphthalimide‐LD‐BFPs.

Within this range, probes achieve high selectivity in LD imaging while ensuring an adequate supply of buffer probes for stable imaging. Conversely, Naphthalimide‐LD‐BFPs with Clog *P* > 4 exhibit “LD hydrophobicity,” leading to strong affinity to LDs but fewer buffer probes in the cytoplasm. Consequently, these probes face challenges in achieving rapid replenishment and fluorescence recovery after photobleaching.

### 2.2 Clog *P* Guides the Development of Multicolor LD‐BFPs

Subsequently, we conducted BuFRAP experiments on NBD‐LD‐BFPs and BDP‐LD‐BFPs to verify the applicability of the “LD buffer window” (2.5 < Clog *P* < 4) for designing LD‐BFPs. As depicted in Figures S3, S5, and S7 (Supporting Information), the “LD hydrophilic” probes **NBD‐DMA** (Clog *P* = 2.48) and **BDP‐MeS** (Clog *P* = 1.98) did not exhibit selective targeting of LDs, whereas other probes enabled wash‐free imaging of LDs. Among the NBD‐LD‐BFPs, **NBD‐DEA** (Clog *P* = 3.54) demonstrated only a modest 10% decrease in fluorescence intensity after intense laser bleaching, indicating rapid replenishment of buffer probes. Despite a *k*_t_ of 0.0320 ± 0.013 s^−1^, it fully recovered to F_0_ within 10 s. Conversely, **LD‐BFP488** (Clog *P* = 4.60) experienced a 55% loss in fluorescence intensity post‐bleaching. Remarkably, its fluorescence recovered to 90% F_0_ within 10 s, with a *k*_t_ of 0.102 ± 0.025 s^−1^. We attribute this to **LD‐BFP488**’s strong affinity for LDs due to its slightly higher Clog *P*. Both **NBD‐DEA** and **LD‐BFP488** achieved stable LD imaging through the buffering strategy. Considering **LD‐BFPh488** exhibited a higher imaging signal‐background ratio than **NBD‐DEA** (Figures S7 and S8, Supporting Information), **LD‐BFP488** appears more suitable for 488 nm laser‐excited LD imaging.

Similarly, for BDP‐LD‐BFPs, **BDP‐2MeS** (Clog *P* = 3.35) demonstrated an increase in fluorescence from ≈25% F_0_ to 75% F_0_ within 40 s post‐bleaching, with a *k*_t_ of 0.0208 ± 0.0037 s^−1^. Conversely, **BDP‐4MeS** (Clog *P* = 4.37) only recovered from 42% F_0_ to 63% F_0_, with a *k*_t_ of 0.00733 ± 0.0014 s^−1^. Fluorescence images also revealed darker LDs in the bleached area compared to the unbleached area at 60 s.

These results confirm that the “LD buffer window” of 2.5 < Clog *P* < 4 is applicable across different types of LD‐BFPs, ensuring effective LD imaging with sufficient buffer probe availability for photostable imaging. LD‐BFPs outside this range either fail to selectively target LDs or exhibit inadequate buffer capacity, leading to slower fluorescence recovery after photobleaching.

In addition, **LD‐BFP405** (Clog *P *= 3.00) and **LD‐BFP543** (Clog *P* = 3.09), two multicolor LD probes with moderate lipophilicity, exhibited robust buffer capacity in BuFRAP experiments (Figure S9, Supporting Information). Both probes showed rapid fluorescence recovery to F_0_ within 40 s, with *k*_t_ values of 0.0610 ± 0.0084 s^−1^ and 0.0455 ± 0.0027 s^−1^, respectively. Notably, **LD‐ BFP543** contains an ester group at the meso‐position, which can undergo hydrolysis to form a carboxylate in the presence of carboxylesterases in living cells, resulting in a blue shift of *λ_em_* to ≈490 nm. Carboxylesterases are predominantly located in liver and intestine cell lines. We hypothesized that **LD‐BFP543** would exhibit greater stability in cell lines with lower carboxylesterase activity, such as HeLa cells. After incubating **LD‐BFP543** in living HeLa cells for 5 h and performing confocal imaging, Figure S8 (Supporting Information) shows that **LD‐BFP543** maintained wash‐free LD imaging capability, with no observable imaging signals in the green channel (488 nm excitation). Moreover, **LD‐BFP543** retained good buffer capacity, albeit with a slightly reduced *k*_t_ of 0.0392 ± 0.040 s^−1^ (Figure S10, Supporting Information).

In contrast, the commercial LD probe LD 540, which has a similar excitation wavelength to **LD‐BFP543** but higher LD hydrophobicity (Clog *P* = 6.00), exhibited a significantly lower *k*_t_ of 0.00205 ± 0.0014 s^−1^. The fluorescence intensity in the selected area showed minimal increase after photobleaching (Figure S11, Supporting Information). These findings underscore the advantage of **LD‐BFP543**’s moderate lipophilicity within the “LD buffer window” (2.5 < Clog *P* < 4), enabling effective LD imaging with robust buffer capacity and stability even under conditions where similar commercial probes fail to perform.

These results confirm that multicolor LD‐BFPs with moderate lipophilicity, namely **LD‐BFP405**, **LD‐BFP450**, **LD‐BFP488**, and **LD‐BFP543**, are capable of achieving highly selective LD imaging and timely replenishment of photobleached probes, thus enabling photostable LD imaging. Moreover, the range of 2.5 < Clog *P* < 4 can serve as a general “LD buffer window” for designing multicolor LD‐BFPs.

It is important to note that while Clog *P* is a significant factor influencing the buffer capacity of LD‐BFPs, it is not the sole determinant. For instance, **Naph‐2Boc** has a slightly higher Clog *P* than **Naph‐2C**, yet it exhibits a slightly higher *k*_t_ than **Naph‐2C**. Additionally, the structural characteristics of LD‐BFPs themselves, such as observed with **LD‐BFP488** and **BDP‐4MeS** where the NBD fluorophore shows higher affinity to LDs, also play a crucial role in probe design. Furthermore, the accumulation of buffer probes in the cytoplasm may introduce background interference in imaging, highlighting the need for further investigation into these influential factors. Future studies should explore these aspects to optimize the design and performance of LD‐BFPs for enhanced LD imaging in living cells.

### 2.3 Buffering Capacity in Cellular Imaging

**Figure 4:**
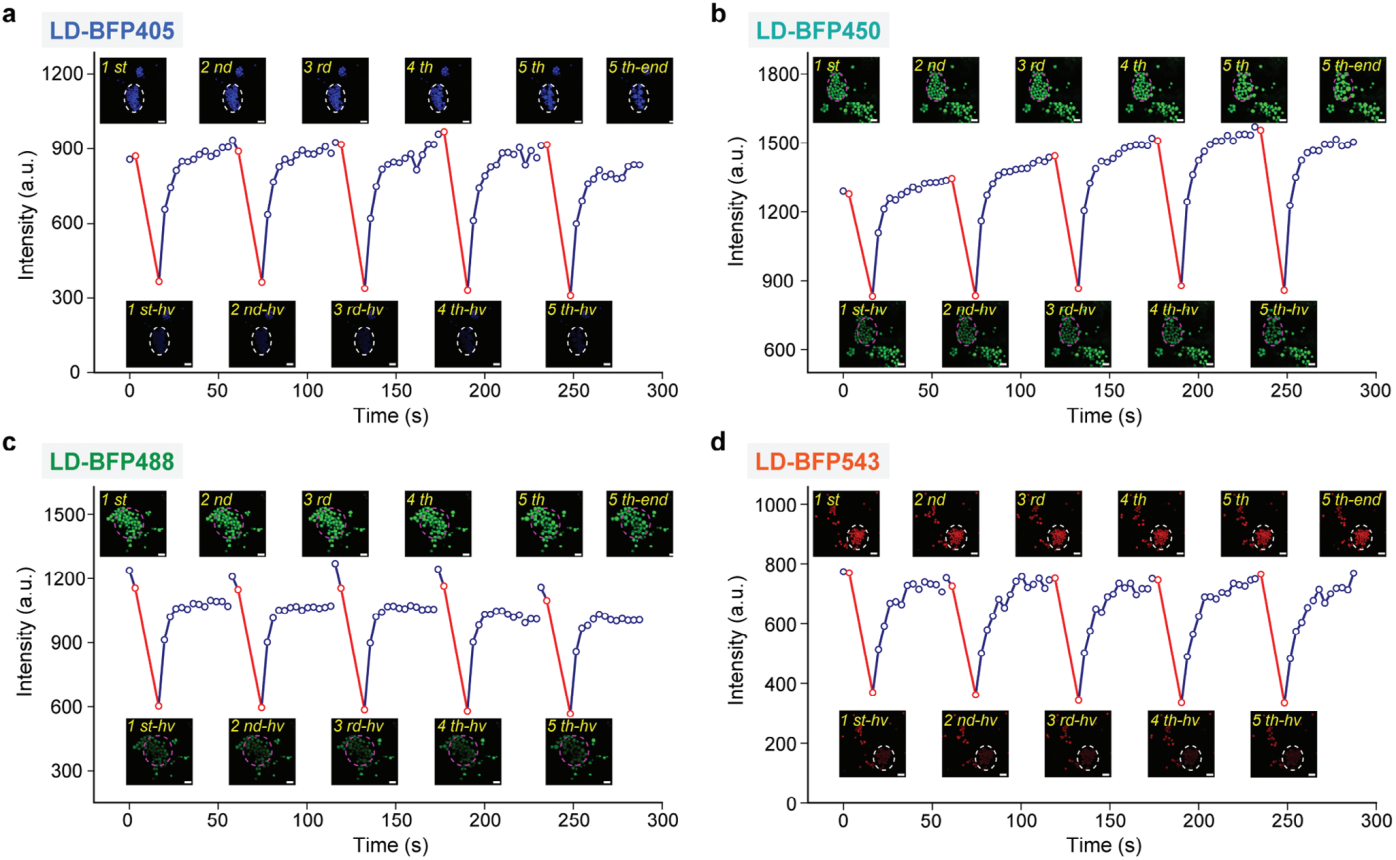
Continuous BuFRAP experiments of 2 µM LD‐BFPs in living HeLa cells. The interval between each BuFRAP experiment was 2 s. The dotted circle highlighted the bleaching area. Scale bar = 2 µm.

Accumulating evidence underscores the dynamic interactions of lipid droplets (LDs) with other organelles. To achieve stable and dynamic imaging of LDs, LD‐BFPs must maintain continuous buffer capacity during long‐term imaging sessions. Therefore, we conducted consecutive BuFRAP experiments on multicolor LD‐BFPs in living cells to assess their sustained buffer capacity. As depicted in **Figure** **4** and Movies S1–S4 (Supporting Information), we subjected selected areas to five intense laser bleaching sessions. **LD‐BFP405**, **LD‐BFP450**, and **LD‐BFP543**, all falling within the “LD buffer window” with moderate lipophilicity, demonstrated rapid restoration of fluorescence intensity to initial levels, maintaining stability throughout the experiment. **LD‐BFP488**, with slightly higher lipophilicity, exhibited weaker photorecovery but stabilized quickly at 85% of initial fluorescence (F_0_) for stable LD imaging. Notably, LD‐LD fusion events were observed in bleached areas, likely due to intense laser perturbation, yet these did not compromise the stable imaging capabilities of these LD‐BFPs. This observation also motivates future efforts to develop high‐brightness LD‐BFPs, enabling the reduction of laser power during imaging procedures.

Afterward, we assessed the photostability of these molecules in living cells using structured illumination microscopy (SIM). We conducted continuous imaging sessions over 15 min at a frame rate of 5 s per frame. The fluorescence intensity of all four probes could be maintained at 70% of initial fluorescence (F_0_), demonstrating their high photostability in LD super‐resolution dynamic imaging (Figure S12, Supporting Information). Additionally, SIM imaging of these probes revealed high spatial resolution, achieving details as fine as 170 nm (Figure S13, Supporting Information). Cytotoxicity experiments further indicated high biocompatibility, as cell viability remained unaffected following staining at a concentration of 5 µM for 24 h (Figure S14, Supporting Information). Based on these findings, **LD‐BFP405**, **LD‐BFP450**, **LD‐BFP488**, and **LD‐BFP543** are deemed suitable for multi‐color LD super‐resolution dynamic imaging.

The experimental results both extracellularly and intracellularly in this study demonstrate the decisive influence of Clog *P* on the buffer capacity of LD‐BFPs. When Clog *P* < 2.5, the molecules exhibit “LD hydrophilicity,” providing a larger buffer pool outside lipid droplets (LDs) but showing weaker affinity toward LDs. This presents two potential drawbacks: first, the probes may not efficiently and selectively target LDs upon cellular entry, thereby failing to enable wash‐free imaging of LDs; second, the slow entry of buffer probes into LDs during photobleaching prevents rapid stabilization of imaging. Conversely, when Clog *P* > 4, the probes display “LD hydrophobicity,” indicating a strong affinity for LDs but with a smaller buffer pool outside LDs. This scenario also has two limitations: excessive probe molecules entering LDs may disturb the physiological state of LDs, and the inadequate buffer pool outside LDs results in insufficient replenishment of photobleached molecules, leading to decreased fluorescence intensity during imaging. In contrast, the range 2.5 < Clog *P* < 4 represents an optimal “LD buffer window.” Here, these molecules exhibit appropriate LD affinity upon cellular entry, with sufficient molecules entering LDs to emit fluorescence, while the buffer pool outside LDs ensures timely replenishment post‐bleaching, facilitating stable dynamic imaging of LDs (Figure 1b).

### 2.4 LD as Storage Site for Overexpress ACSL3 During Ferroptosis

Ferroptosis, a form of programmed cell death catalyzed by redox‐active iron, involves the oxidation of polyunsaturated fatty acid‐containing phospholipids.^[^ ^14^ ^]^ There is accumulating evidence suggesting that modulating ferroptotic cell death can offer substantial clinical benefits for certain diseases.^[^ ^15^ ^]^ Recent studies have highlighted the involvement of lipid droplets (LDs) in the regulation of ferroptosis.^[^ ^16^ ^]^ LDs play multifaceted roles as signaling precursors and cellular stress managers, interacting with various organelles to regulate biological processes. Multicolor imaging techniques enable the exploration of LD interactions with other cellular targets, potentially unveiling novel LD functions.

Using our multicolor LD‐BFPs for tracking LD dynamics via structured illumination microscopy (SIM), we observed during ferroptosis that overexpressed ACSL3 proteins induce endoplasmic reticulum (ER) stress and are subsequently secreted into the cytoplasm within vesicles. These highly active vesicles engage in extensive interactions with LDs, which serve as transient storage sites for the excess proteins through membrane fusion events with the vesicles. The proteins are then likely degraded through active recruitment involving interactions with other organelles such as lysosomes or proteasomes, thereby maintaining intracellular homeostasis.

**Figure 5:**
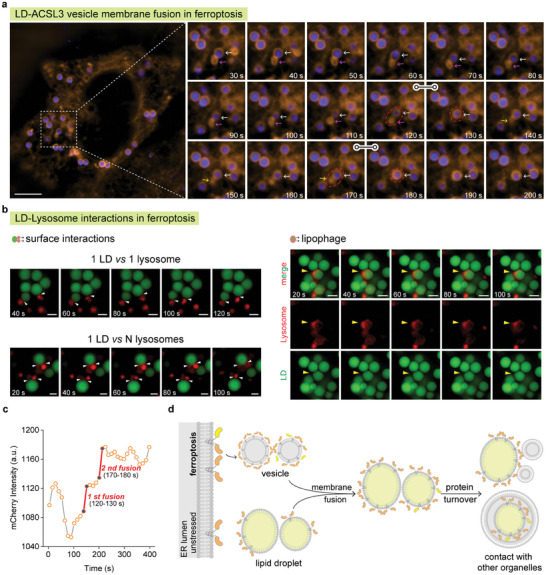
The role of LD dynamics during cellular ferroptosis. a) Membrane fusion of LD and ACSL3 vesicle in ferroptosis. ACSL3 labeled with mCherry (orange) via the genetically encoded method, LDs stained with 2 µM **LD‐BFP405** (blue). b) The LD‐Lysosome contact in ferroptosis. LDs stained with 2 µM **LD‐BFP488** (green) and lysosome stained by a lysosome probe (red) developed by our lab. HeLa cells were treated with 10 µM sorafenib for 3 h to induce ferroptosis. Scale bar = 5 µm. c) Relative intensity changes of ACSL3‐mcherry on LD membrane during membrane fusion. d) LD as a protein “transit station” during ferroptosis.

ACSL3, a class I protein found in both the endoplasmic reticulum (ER) bilayer and the monolayer of lipid droplets (LDs),^[^ ^17^ ^]^ has been implicated in ferroptosis due to its overexpression,^[^ ^18^ ^]^ which promotes a ferroptosis‐resistant state. To investigate the role of ACSL3 and LDs during ferroptosis, we transfected HeLa cells to express a fusion protein, ACSL3‐mCherry, for labeling ACSL3. Concurrently, LDs were labeled using **LD‐BFP405**. After treating the cells with 10 µM sorafenib for 3 h to induce ferroptosis, we observed several intriguing phenomena. As depicted in Figure S15 and Movie S5 (Supporting Information), sorafenib treatment resulted in the formation of numerous ACSL3‐mCherry puncta and small vesicles within the cells. These vesicles exhibited rapid intracellular transport, and notably, they frequently interacted with LDs, particularly on LD membranes. This interaction often involved the formation of “LD‐vesicle complexes” or transient interactions termed “kiss and run.” Of particular interest were two instances of rapid membrane fusion events observed between ACSL3 vesicles and LDs at ≈120–130 s and 170–180 s into the observation period. Following these fusions, there was a significant increase in the fluorescence intensity of mCherry on the fused LD surfaces (**Figures** **5**a,c).

These observations suggest two potential roles of LDs in the context of ferroptosis induced by ACSL3 overexpression: First, LDs may be directly targeted by cells overexpressing ACSL3 to regulate LD functions. Second, the overexpression of ACSL3 could lead to protein misfolding within the ER membrane. These misfolded and overloaded proteins are then secreted into the cytoplasm in vesicular form. Nearby LDs can subsequently recruit these vesicles and incorporate the proteins into LD membranes through membrane fusion events. LDs thus serve as temporary storage sites for these proteins or facilitate their degradation through interactions with other organelles, thereby contributing to intracellular homeostasis maintenance. These findings underscore the dynamic and multifaceted roles of LDs in cellular processes, particularly in the context of ferroptosis regulation.

To delve deeper into the fate of overexpressed proteins during ferroptosis, we utilized **LD‐BFP488** to label lipid droplets (LDs) and a custom‐developed lysosome probe in our experiments. Figure 5b illustrates two potential pathways involving lysosomal‐mediated protein degradation. First, we observed numerous transient interactions between lysosomes and the surface of LD membranes. These interactions suggest that LDs can facilitate the degradation of overexpressed proteins by presenting them to lysosomes on their membrane surfaces. This process likely involves the direct fusion of LDs with one or more lysosomes, enabling the transfer and subsequent degradation of proteins within the lysosomal environment. Second, we identified the potential involvement of lipophagy, a crucial pathway for protein turnover (Figure S16, Supporting Information). Lysosomes contain a plethora of hydrolytic enzymes capable of degrading proteins associated with LD membranes. This mechanism underscores an alternative route through which LDs contribute to the turnover and maintenance of cellular homeostasis during ferroptosis.

These findings collectively highlight the pivotal role of LDs in the regulation of ferroptosis. By acting as temporary storage sites through membrane fusion and facilitating interactions with organelles like lysosomes, LDs play a crucial role in maintaining cellular homeostasis. The application of photostable, multi‐color LD‐BFPs has been instrumental in uncovering these novel functions of LDs (Figure 5d), furthering our understanding of their dynamic involvement in cellular processes, particularly in contexts such as ferroptosis regulation.

We further investigated the active recruitment process of lipid droplets (LDs) to other organelles, observing dynamic interactions facilitated by **LD‐BFP450** in living HeLa cells. Our observations, leveraging the high photostability of LD‐BFPs, revealed intriguing recruitment dynamics. In the case of LDs and lysosomes, we tracked the recruitment process using **LD‐BFP450**. Initially, a lysosome gradually approached an LD, and over the subsequent 180 s, they moved together to form an “LD‐Lysosome complex”. Subsequently, another lysosome from a distance joined this complex, eventually interacting with the LD on its membrane surface (Figure S17, Movie S6, Supporting Information).

Additionally, we observed the active recruitment of a large LD cluster toward a smaller LD cluster (Figure S18, Movie S7, Supporting Information). These findings suggest that LDs may play an active role as recruiters in various biological processes, possibly mediating interactions between different organelles. The molecular mechanisms underlying these phenomena warrant further investigation. However, our study underscores the dynamic and versatile nature of LDs in cellular biology, facilitated by the use of photostable, multi‐color LD‐BFPs to illuminate these intricate processes in living cells.

## 3 Conclusion

In conclusion, our study highlights the significant impact of lipophilicity on the buffer capacity of LD‐BFPs, emphasizing the utility of the Clog *P* descriptor for the rational development of LD‐BFPs. Specifically, probes with Clog *P* < 2.5 exhibit “LD hydrophilicity,” lacking selective LD targeting; those with Clog *P* > 4 demonstrate “LD hydrophobicity,” characterized by insufficient buffer outside LDs and inadequate replenishment post‐photobleaching. The range 2.5 < Clog *P* < 4 represents an optimal “LD buffer window,” facilitating both LD selectivity and photostable dynamic imaging. Building on these insights, we developed four multicolor LD‐BFPs—**LD‐BFP405**, **LD‐BFP450**, **LD‐BFP488**, and **LD‐BFP543**—for super‐resolution dynamic imaging of LDs. Utilizing SIM imaging, we achieved high temporal‐spatial resolution and observed LDs functioning as protein “recruiters” and “transit stations” during ferroptosis. The interaction between LD recruiter proteins and other organelles is crucial for intracellular homeostasis. These findings contribute significantly to uncovering novel roles of LDs in cell biology.

## Conflict of Interest

The authors declare no conflict of interest.

## Supporting information



Supporting Information

Supplemental Movie 1

Supplemental Movie 2

Supplemental Movie 3

Supplemental Movie 4

Supplemental Movie 5

Supplemental Movie 6

Supplemental Movie 7

## Data Availability

The data that support the findings of this study are available in the supplementary material of this article.
