# Supplementary material for: Clog P‐Guided Development of Multi‐Colored Buffering Fluorescent Probes for Super‐Resolution Imaging of Lipid Droplet Dynamics
Source: Adv Sci (Weinh). 2024 Oct 30;11(48):2408030. doi: 10.1002/advs.202408030 (PMC11672300; doi:10.1002/advs.202408030)
Supplement: Supplementary file 1 — Supporting Information [file ADVS-11-2408030-s001.pdf]

ADVANCED SCIENCE
Open Access

Supporting Information

for *Adv. Sci.*, DOI 10.1002/advs.202408030

Clog P-Guided Development of Multi-Colored Buffering Fluorescent Probes for Super-Resolution Imaging of Lipid Droplet Dynamics

Jie Chen, Qinglong Qiao, Hanlixin Wang, Wenchao Jiang, Wenjuan Liu, Kai An and Zhaochao Xu**

Supporting Information

Clog P-Guided Development of Multi-Colored Buffering Fluorescent Probes for Super-Resolution Imaging of Lipid Droplet Dynamics

Jie Chen, *Qinglong Qiao**, *Hanlixin Wang*, *Wenchao Jiang*, *Wenjuan Liu*, *Kai An*, *Zhaochao Xu**

1. Materials and Instruments

1.1. Materials

Unless otherwise specifically stated, all reagents were purchased from commercial suppliers (Sigma-Aldrich, *J&K*, Innochem and Aladdin) and used without further purification. Solvents [dimethyl sulfoxide (DMSO), methanol, ethanol, chloroform and acetonitrile] were purchased from *J&K* and used without further treatment or distillation. Silica gel (200-300 mesh) was purchased from Innochem.

1.2. Instruments

^{1}H-NMR and ^{13}C-NMR spectra were recorded on a Bruker 400 spectrometer, using TMS as an internal standard. Chemical shifts were given in ppm and coupling constants (J) in Hz. High-resolution mass spectrometry (HRMS) data were obtained using ESI (6540 UHD Q-TOF, positive ion).

For UV-vis absorption spectra were collected on an Agilent Cary 60 UV-Vis Spectrophotometer. Fluorescence measurements were performed on an Agilent CARY Eclipse fluorescence spectrophotometer.

Confocal images were performed on Olympus FV1000 MPE with a microscope IX 71, a 100× / NA 1.40 oil objective lens, LU-NV series laser unit (laser combination: 405 nm; 543 nm; 488 nm; 635 nm). Single photon confocal microscope system was built on an Olympus inverted microscope IX81.

Structured illumination microscopy (SIM) super-resolution images were taken on a Nikon N-SIM system with a 100×oil immersion objective lens, 1.49 NA (Nikon). Images were captured using Nikon NIS-Elements and reconstructed using slice reconstruction in NIS-elements.

2. Synthesis of BF-probe

2.1 Synthesis of Naph-OH

Synthesis of **OH-Br**

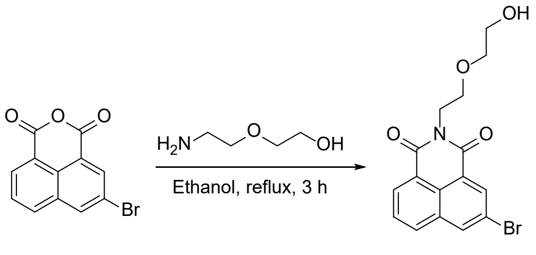

OH-Br

2-(2-Aminoethoxy)ethanol (114 mg, 1.08 mmol) was dissolved in 2 mL ethanol, then added to a suspension of 3-bromo-1,8-naphthalic anhydride (100 mg, 0.36 mmol) in 5 mL

ethanol. After stirred and refluxed for 3 h, the mixture was cooled to room temperature. The solvent was removed under reduced pressure and the residue was purified by flash column chromatography (DCM:EA = 30:1) to obtain a white solid of 42 mg, yield 32%.

^{1}H NMR (400 MHz, $CDCl_3$) δ 8.57 (d, *J* = 1.5 Hz, 1H), 8.55 (d, *J* = 7.3 Hz, 1H), 8.30 (d, *J* = 1.3 Hz, 1H), 8.08 (d, *J* = 8.2 Hz, 1H), 7.74 (t, *J* = 7.8 Hz, 1H), 4.41 (t, *J* = 5.6 Hz, 2H), 3.85 (t, *J* = 5.6 Hz, 2H), 3.70 (d, *J* = 4.5 Hz, 2H), 3.68-3.64 (m, 2H), 2.58 (s, 1H).

^{13}C NMR (100 MHz, $CDCl_3$) δ 163.86, 163.24, 135.58, 134.08, 132.91, 132.72, 131.53, 128.03, 126.53, 123.92, 122.58, 121.09, 72.28, 68.31, 61.81, 39.68.

HRMS (ESI) m/z Found 364.0172 $(M+H)^+$, calculated 364.0184 for $C_{16}H_{15}BrNO_4^+$.

Synthesis of **Naph-OH**

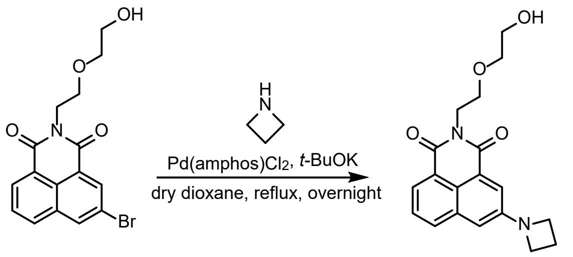

OH-Br **Naph-OH**

At nitrogen atmosphere, **OH-Br** (40 mg, 0.11 mmol), Pd(amphos)Cl_2 (12 mg, 0.017 mmol), *t*-BuOK (39 mg, 0.35 mmol) was suspended into 3 mL dry toluene, azetidine (57 μL, 0.85 mmol) was dissolved in 2 mL dry dixone then added to the reaction mixture by injection syringe. The reaction was heated to reflux, after stirring overnight in a nitrogen atmosphere, solvent was removed under reduced pressure and the residue was further purified by flash column chromatography (DCM:MeOH = 100:1) to obtain an orange solid of 14 mg, yield 37%.

^{1}H NMR (400 MHz, $CDCl_3$) δ 8.26 (d, *J* = 7.1 Hz, 1H), 7.91 (d, *J* = 8.2 Hz, 1H), 7.78 (d, *J* = 2.3 Hz, 1H), 7.61-7.53 (m, 1H), 6.89 (d, *J* = 2.2 Hz, 1H), 6.89 (d, *J* = 2.2 Hz, 1H), 4.41 (t, *J* = 5.7 Hz, 2H), 4.05 (t, *J* = 7.3 Hz, 4H), 3.84 (t, *J* = 5.7 Hz, 2H), 3.70 (d, *J* = 4.4 Hz, 2H), 3.66 (dd, *J* = 4.8, 3.2 Hz, 2H), 2.59 (s, 1H), 2.52-2.43 (m, 2H).

^{13}C NMR (100 MHz, $CDCl_3$) δ 164.84, 164.73, 150.08, 133.23, 131.87, 127.09, 127.07, 122.92, 122.21, 121.73, 118.57, 110.26, 72.24, 68.48, 61.86, 52.35, 39.45, 16.87.

HRMS (ESI) m/z Found 341.1494 $(M+H)^+$, calculated 341.1501 for $C_{19}H_{21}N_2O_4^+$.

2.2 Synthesis of probe LD-BFP450

Synthesis of **Mor-Br**

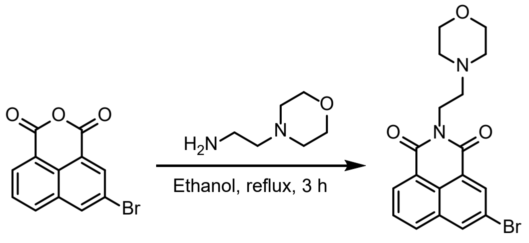

Mor-Br

4-(2-Aminoethyl)morpholine (130 mg, 1 mmol) was dissolved in 2 mL ethanol, then added to a suspension of 3-bromo-1,8-naphthalic anhydride (100 mg, 0.36 mmol) in 5 mL ethanol. After stirred and refluxed for 3 h, the mixture was cooled to room temperature. The solvent was removed under reduced pressure and the residue was purified by flash column chromatography (DCM:EA = 50:1) to obtain a white solid of 82 mg, yield 58%.

^{1}H NMR (400 MHz, $CDCl_3$) δ 8.64 (s, 1H), 8.58 (d, *J* = 7.2 Hz, 1H), 8.36 (s, 1H), 8.12 (d, *J* = 8.3 Hz, 1H), 7.78 (t, *J* = 7.7 Hz, 1H), 4.34 (dd, *J* = 14.4, 7.7 Hz, 2H), 3.71-3.63 (m, 4H), 2.70 (t, *J* = 6.7 Hz, 2H), 2.59 (s, 4H).

^{13}C NMR (100 MHz, $CDCl_3$) δ 163.64, 163.01, 135.48, 133.98, 132.82, 132.79, 131.35, 128.04, 126.62, 124.15, 122.80, 121.12, 67.03, 56.10, 53.82, 37.38.

HRMS (ESI) m/z Found 389.0473 $(M+H)^+$, calculated 389.0501 for $C_{18}H_{18}BrN_2O_3^+$.

<u>Synthesis of **LD-BFP450**</u>

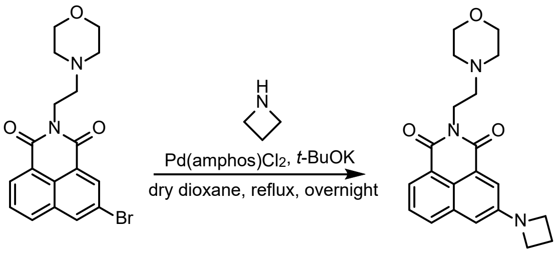

Mor-Br **LD-BFP450**

At nitrogen atmosphere, **Mor-Br** (50 mg, 0.13 mmol), Pd(amphos)Cl_2 (12 mg, 0.017 mmol), *t*-BuOK (39 mg, 0.35 mmol) was suspended into 3 mL dry toluene, azetidine (57 μL, 0.85 mmol) was dissolved in 2 mL dry dixone then added to the reaction mixture by injection syringe. The reaction was heated to reflux, after stirring overnight in a nitrogen atmosphere, solvent was removed under reduced pressure and the residue was further purified by flash column chromatography (DCM:MeOH = 200:1) to obtain an orange solid of 16 mg, yield 35%.

^{1}H NMR (400 MHz, $CDCl_3$) δ 8.25 (d, *J* = 7.2 Hz, 1H), 7.91 (d, *J* = 8.3 Hz, 1H), 7.77 (t, *J* = 3.2 Hz, 1H), 7.57 (t, *J* = 7.7 Hz, 1H), 6.89 (d, *J* = 1.6 Hz, 1H), 4.32 (t, *J* = 7.0 Hz, 2H), 4.05 (t, *J* = 7.2 Hz, 4H), 3.71-3.65 (m, 4H), 2.69 (t, *J* = 7.0 Hz, 2H), 2.60 (s, 4H), 2.53-2.41 (m, 2H).

^{13}C NMR (100 MHz, $CDCl_3$) δ 164.51, 164.42, 150.07, 133.91, 133.24, 131.71, 131.16, 127.08, 126.92, 126.85, 123.06, 122.36, 121.70, 118.37, 110.10, 67.08, 56.20, 53.84, 52.34, 37.16, 16.88.

HRMS (ESI) m/z Found 366.1807 $(M+H)^+$, calculated 366.1808 for $C_{21}H_{24}N_3O_3^+$.

2.3 Synthesis of Naph-2C

Synthesis of **2C-Br**

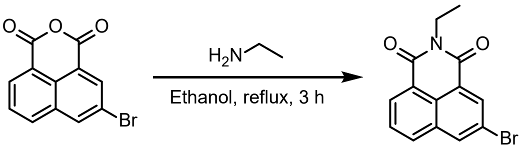

2C-Br

Ethylamine (2 mol/L in THF) (0.5 mL, 1 mmol) was added to a suspension of 3-bromo-1,8-naphthalic anhydride (100 mg, 0.36 mmol) in 5 mL ethanol. After stirred and refluxed for 3 h, the mixture was cooled to room temperature. The solvent was removed under reduced pressure and the residue was purified by flash column chromatography (PE:DCM = 5:1) to obtain a white solid of 82 mg, yield 75%.

^{1}H NMR (400 MHz, $CDCl_3$) δ 8.63-8.54 (m, 2H), 8.31 (s, 1H), 8.09 (d, J = 8.0 Hz, 1H), 7.75 (t, J = 7.5 Hz, 1H), 4.22 (d, J = 6.8 Hz, 2H), 1.33 (t, J = 6.6 Hz, 3H).

^{13}C NMR (100 MHz, $CDCl_3$) δ 163.39, 162.76, 135.35, 133.86, 132.77, 132.67, 131.24, 127.98, 126.53, 124.25, 122.89, 121.07, 35.69, 13.31.

HRMS (ESI) m/z Found 303.9967 $(M+H)^+$, calculated 303.9973 for $C_{14}H_{11}BrNO_2^+$.

Synthesis of **Naph-2C**

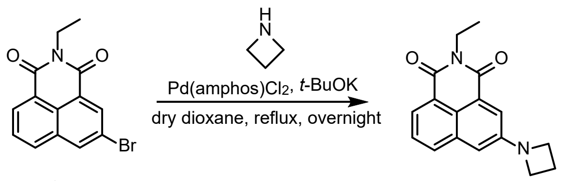

2C-Br Naph-2C

At nitrogen atmosphere, **2C-Br** (50 mg, 0.17 mmol), Pd(amphos)Cl_2 (12 mg, 0.017 mmol), *t*-BuOK (39 mg, 0.35 mmol) was suspended into 3 mL dry toluene, azetidine (57 μL, 0.85 mmol) was dissolved in 2 mL dry dixone then added to the reaction mixture by injection syringe. The reaction was heated to reflux, after stirring overnight in a nitrogen atmosphere, solvent was removed under reduced pressure and the residue was further purified by flash column chromatography (DCM:MeOH = 200:1) to obtain an orange solid of 30 mg, yield 65%.

^{1}H NMR (400 MHz, $CDCl_3$) δ 8.28 (d, *J* = 7.2 Hz, 1H), 7.92 (d, *J* = 8.2 Hz, 1H), 7.82 (d, *J* = 1.9 Hz, 1H), 7.58 (t, *J* = 7.8 Hz, 1H), 6.91 (d, *J* = 1.6 Hz, 1H), 4.22 (q, *J* = 7.0 Hz, 2H), 4.07 (t, *J* = 7.2 Hz, 4H), 2.55-2.42 (m, 2H), 1.32 (t, *J* = 7.0 Hz, 3H).

^{13}C NMR (100 MHz, $CDCl_3$) δ 164.39, 164.28, 150.14, 133.25, 131.64, 127.08, 126.82, 123.26, 122.54, 121.73, 118.40, 110.09, 52.40, 35.43, 16.89, 13.38.

HRMS (ESI) m/z Found 281.1290 $(M+H)^+$, calculated 281.1284 for $C_{17}H_{17}N_2O_2^+$.

2.4 Synthesis of Naph-2Boc

<u>Synthesis of **2Boc-Br**</u>

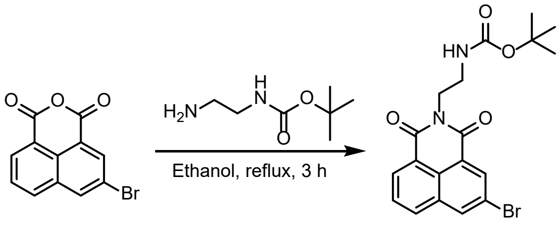

2Boc-Br

t-Butyl 2-aminoethylcarbamate (160 mg, 1 mmol) was dissolved in 2 mL ethanol, then added to a suspension of 3-bromo-1,8-naphthalic anhydride (100 mg, 0.36 mmol) in 5 mL ethanol. After stirred and refluxed for 3 h, the mixture was cooled to room temperature. The solvent was removed under reduced pressure and the residue was purified by flash column chromatography (PE:DCM = 5:1) to obtain a white solid of 80 mg, yield 53%.

^{1}H NMR (400 MHz, $CDCl_3$) δ 8.59 (d, *J* = 1.3 Hz, 1H), 8.56 (d, *J* = 7.2 Hz, 1H), 8.31 (s, 1H), 8.09 (d, *J* = 8.2 Hz, 1H), 7.75 (t, *J* = 7.8 Hz, 1H), 4.98 (s, 1H), 4.33 (t, *J* = 5.5 Hz, 2H), 3.53 (d, *J* = 4.8 Hz, 2H), 1.27 (s, 9H).

^{13}C NMR (100 MHz, $CDCl_3$) δ 163.93, 163.34, 156.07, 135.52, 134.08, 132.85, 132.73, 131.50, 128.00, 126.61, 123.97, 122.63, 121.06, 40.09, 39.49, 31.58, 28.20, 22.65, 14.12.

HRMS (ESI) m/z Found 419.0594 $(M+H)^+$, calculated 419.0606 for $C_{19}H_{20}BrN_2O_4^+$.

<u>Synthesis of **Naph-2Boc**</u>

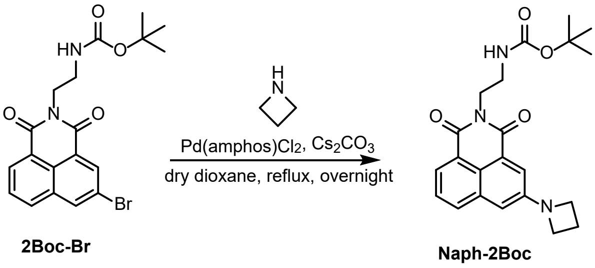

2Boc-Br **Naph-2Boc**

At nitrogen atmosphere, **2Boc-Br** (50 mg, 0.12 mmol), Pd(amphos)Cl_2 (12 mg, 0.017 mmol), Cs_2CO_3 (114 mg, 0.35 mmol) was suspended into 3 mL dry toluene, azetidine (57 μL,

0.85 mmol) was dissolved in 2 mL dry dixone then added to the reaction mixture by injection syringe. The reaction was heated to reflux, after stirring overnight in a nitrogen atmosphere, solvent was removed under reduced pressure and the residue was further purified by flash column chromatography (DCM:MeOH = 300:1) to obtain an orange solid of 25.5 mg, yield 54%.

^{1}H NMR (400 MHz, $CDCl_3$) δ 8.25 (d, J = 7.2 Hz, 1H), 7.91 (d, J = 8.2 Hz, 1H), 7.78 (d, J = 1.6 Hz, 1H), 7.56 (t, J = 7.8 Hz, 1H), 6.89 (s, 1H), 5.04 (s, 1H), 4.36-4.27 (m, 2H), 4.06 (t, J = 7.2 Hz, 4H), 3.52 (d, J = 4.7 Hz, 2H), 2.53-2.44 (m, 2H), 1.32 (s, 9H).

^{13}C NMR (100 MHz, $CDCl_3$) δ 164.94, 164.77, 156.05, 150.06, 133.20, 131.83, 127.05, 122.88, 122.17, 120.53, 118.58, 110.22, 52.35, 39.72, 28.26, 16.87.

HRMS (ESI) m/z Found 396.1916 $(M+H)^+$, calculated 396.1923 for $C_{22}H_{26}N_3O_4^+$.

2.5 Synthesis of Naph-4Boc

<u>Synthesis of **4Boc-Br**</u>

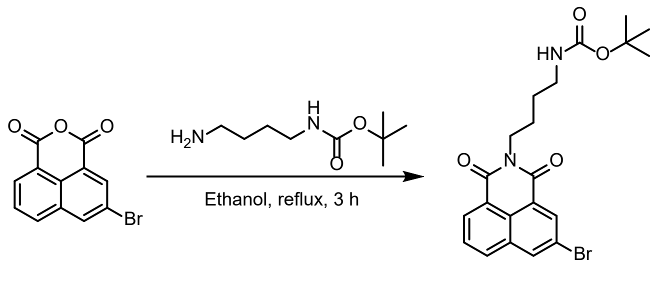

t-Butyl 2-aminobutylcarbamate (190 mg, 1 mmol) was dissolved in 2 mL ethanol, then added to a suspension of 3-bromo-1,8-naphthalic anhydride (100 mg, 0.36 mmol) in 5 mL ethanol. After stirred and refluxed for 3 h, the mixture was cooled to room temperature. The solvent was removed under reduced pressure and the residue was purified by flash column chromatography (PE:DCM = 4:1) to obtain a white solid of 80 mg, yield 49.3%.

^{1}H NMR (400 MHz, $CDCl_3$) δ 8.62 (s, 1H), 8.58 (d, J = 7.1 Hz, 1H), 8.35 (s, 1H), 8.12 (d, J = 8.1 Hz, 1H), 7.77 (t, J = 7.8 Hz, 1H), 4.66 (s, 1H), 4.18 (t, J = 7.3 Hz, 2H), 3.19 (d, J = 6.0 Hz, 2H), 1.83 – 1.70 (m, 4H), 1.61 (dd, J = 14.7, 7.2 Hz, 2H), 1.43 (s, 9H).

^{13}C NMR (100 MHz, $CDCl_3$) δ 163.64, 163.02, 155.96, 135.51, 134.02, 132.82, 131.40, 128.03, 126.59, 124.15, 122.78, 121.10, 79.10, 40.20, 40.07, 28.42, 27.55, 25.37.

HRMS (ESI) m/z Found 447.0900 $(M+H)^+$, calculated 419.0919 for $C_{21}H_{24}BrN_2O_4^+$.

<u>Synthesis of **Naph-4Boc**</u>

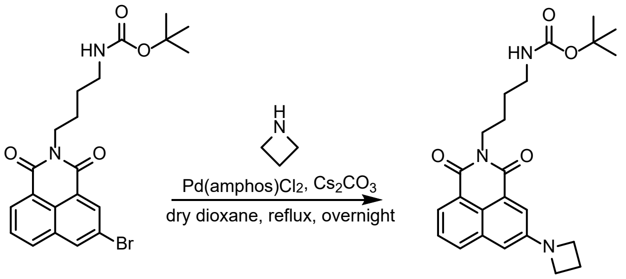

4Boc-Br **Naph-4Boc**

At nitrogen atmosphere, **4Boc-Br** (50 mg, 0.11 mmol), Pd(amphos)Cl_2 (12 mg, 0.017 mmol), Cs_2CO_3 (114 mg, 0.35 mmol) was suspended into 3 mL dry toluene, azetidine (57 μL, 0.85 mmol) was dissolved in 2 mL dry dixone then added to the reaction mixture by injection syringe. The reaction was heated to reflux, after stirring overnight in a nitrogen atmosphere, solvent was removed under reduced pressure and the residue was further purified by flash column chromatography (DCM:MeOH = 300:1) to obtain an orange solid of 28 mg, yield 61%.

^{1}H NMR (400 MHz, $CDCl_3$) δ 8.27 (d, *J* = 7.1 Hz, 1H), 7.94 (d, *J* = 8.2 Hz, 1H), 7.82 (d, *J* = 2.1 Hz, 1H), 7.59 (t, *J* = 7.8 Hz, 1H), 6.93 (d, *J* = 2.1 Hz, 1H), 4.64 (s, 1H), 4.23-4.13 (m, 2H), 4.08 (t, *J* = 7.3 Hz, 4H), 3.19 (d, *J* = 6.1 Hz, 2H), 2.55-2.42 (m, 2H), 1.76 (dt, *J* = 15.1, 7.7 Hz, 2H), 1.63-1.56 (m, 2H), 1.43 (s, 9H).

^{13}C NMR (100 MHz, $CDCl_3$) δ 164.59, 164.49, 155.96, 150.16, 133.26, 131.76, 127.11, 126.94, 123.10, 122.39, 121.73, 118.50, 110.21, 79.04, 52.40, 40.24, 39.83, 28.43, 27.55, 25.46, 16.89.

HRMS (ESI) m/z Found 424.2231 $(M+H)^+$, calculated 424.2236 for $C_{24}H_{30}N_3O_4^+$.

2.6 Synthesis of Naph-4C

Synthesis of **4C-Br**

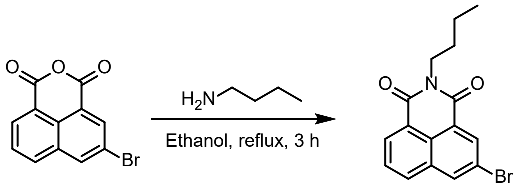

4C-Br

Butylamine (73 mg, 1 mmol) was dissolved in 2 mL ethanol, then added to a suspension of 3-bromo-1,8-naphthalic anhydride (100 mg, 0.36 mmol) in 5 mL ethanol. After stirred and refluxed for 3 h, the mixture was cooled to room temperature. The solvent was removed under reduced pressure and the residue was purified by flash column chromatography (PE:DCM = 20:1) to obtain a white solid of 76 mg, yield 65%.

^{1}H NMR (400 MHz, $CDCl_3$) δ 8.64 (d, *J* = 1.7 Hz, 1H), 8.59 (d, *J* = 7.3 Hz, 1H), 8.35 (d, *J* = 1.7 Hz, 1H), 8.11 (d, *J* = 8.2 Hz, 1H), 7.77 (t, *J* = 7.8 Hz, 1H), 4.20-4.14 (m, 2H), 1.76-1.66 (m, 2H), 1.49-1.39 (m, 2H), 0.98 (t, *J* = 7.3 Hz, 3H).

^{13}C NMR (100 MHz, $CDCl_3$) δ 163.67, 163.04, 135.40, 133.98, 132.84, 132.70, 131.32, 128.01, 126.63, 124.31, 122.94, 121.10, 58.49, 40.43, 30.17, 20.35, 18.45, 13.83.

HRMS (ESI) m/z Found 332.0277 $(M+H)^+$, calculated 332.0286 for $C_{16}H_{15}BrNO_2^+$.

Synthesis of **Naph-4C**

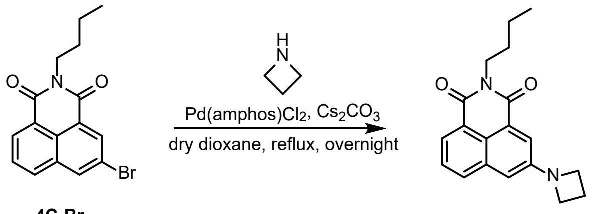

4C-Br **Naph-4C**

At nitrogen atmosphere, **4C-Br** (50 mg, 0.15 mmol), Pd(amphos)Cl_2 (12 mg, 0.017 mmol), Cs_2CO_3 (114 mg, 0.35 mmol) was suspended into 3 mL dry toluene, azetidine (57 μL, 0.85 mmol) was dissolved in 2 mL dry dixone then added to the reaction mixture by injection syringe. The reaction was heated to reflux, after stirring overnight in a nitrogen atmosphere, solvent was removed under reduced pressure and the residue was further purified by flash column chromatography (DCM) to obtain an orange solid of 32.5 mg, yield 70%.

^{1}H NMR (400 MHz, $CDCl_3$) δ 8.27 (d, J = 7.2 Hz, 1H), 7.92 (d, J = 8.2 Hz, 1H), 7.81 (d, J = 2.2 Hz, 1H), 7.57 (t, J = 7.8 Hz, 1H), 6.91 (d, J = 2.0 Hz, 1H), 4.21-4.13 (m, 2H), 4.06 (t, J = 7.2 Hz, 4H), 2.54-2.41 (m, 2H), 1.71 (dt, J = 15.2, 7.6 Hz, 2H), 1.50-1.38 (m, 2H), 0.98 (t, J = 7.3 Hz, 3H).

^{13}C NMR (100 MHz, $CDCl_3$) δ 164.58, 164.48, 150.15, 133.24, 131.61, 127.08, 126.84, 123.23, 122.52, 121.73, 118.42, 110.06, 52.38, 40.17, 30.25, 20.39, 16.89, 13.86.

HRMS (ESI) m/z Found 309.1594 $(M+H)^+$, calculated 309.1603 for $C_{19}H_{21}N_2O_2^+$.

2.7 Synthesis of Naph-8C

Synthesis of **8C-Br**

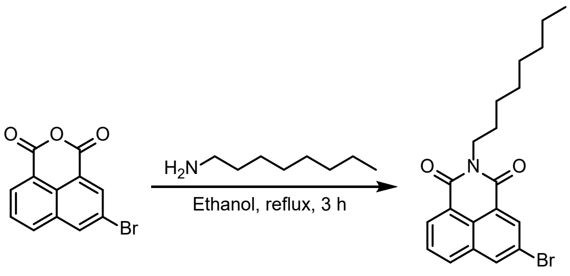

8C-Br

Octylamine (130 mg, 1 mmol) was dissolved in 2 mL ethanol, then added to a suspension of 3-bromo-1,8-naphthalic anhydride (100 mg, 0.36 mmol) in 5 mL ethanol. After stirred and refluxed for 3 h, the mixture was cooled to room temperature. The solvent was removed under

reduced pressure and the residue was purified by flash column chromatography (PE:DCM = 5:1) to obtain a white solid of 56 mg, yield 40%.

^{1}H NMR (400 MHz, $CDCl_3$) δ 8.65 (d, *J* = 1.6 Hz, 1H), 8.59 (d, *J* = 7.3 Hz, 1H), 8.35 (s, 1H), 8.11 (d, *J* = 8.2 Hz, 1H), 7.77 (t, *J* = 7.8 Hz, 1H), 4.21-4.12 (m, 2H), 1.79-1.66 (m, 2H), 1.48-1.22 (m, 10H), 0.87 (t, *J* = 6.6 Hz, 3H).

^{13}C NMR (100 MHz, $CDCl_3$) δ 163.66, 163.03, 135.40, 133.99, 132.84, 132.70, 131.32, 128.02, 126.64, 124.32, 122.95, 121.10, 40.69, 31.82, 29.33, 29.21, 28.09, 27.12, 22.64, 14.10.

HRMS (ESI) m/z Found 388.0908 $(M+H)^+$, calculated 388.0912 for $C_{20}H_{23}BrNO_2^+$.

Synthesis of **Naph-8C**

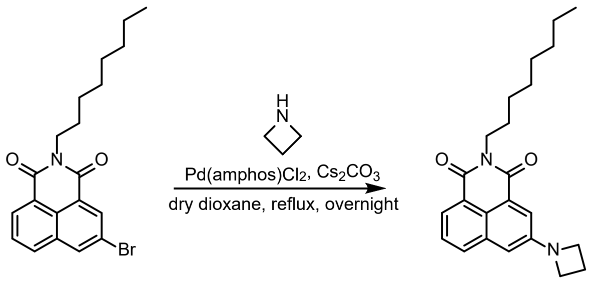

8C-Br **Naph-8C**

At nitrogen atmosphere, **8C-Br** (50 mg, 0.15 mmol), Pd(amphos)Cl_2 (12 mg, 0.017 mmol), Cs_2CO_3 (114 mg, 0.35 mmol) was suspended into 3 mL dry toluene, azetidine (57 μL, 0.85 mmol) was dissolved in 2 mL dry dixone then added to the reaction mixture by injection syringe. The reaction was heated to reflux, after stirring overnight in a nitrogen atmosphere, solvent was removed under reduced pressure and the residue was further purified by flash column chromatography (PE:DCM = 1:2) to obtain an orange solid of 17.5 mg, yield 37%.

^{1}H NMR (400 MHz, $CDCl_3$) δ 8.27 (d, *J* = 7.2 Hz, 1H), 7.93 (d, *J* = 8.2 Hz, 1H), 7.82 (s, 1H), 7.58 (t, *J* = 7.7 Hz, 1H), 6.92 (s, 1H), 4.19-4.11 (m, 2H), 4.07 (t, *J* = 7.1 Hz, 4H), 2.54-2.42 (m, 2H), 1.77-1.66 (m, 2H), 1.40-1.20 (m, 10H), 0.86 (d, *J* = 6.6 Hz, 3H).

^{13}C NMR (100 MHz, $CDCl_3$) δ 164.57, 164.47, 150.16, 133.25, 131.62, 127.09, 126.86, 123.25, 122.53, 121.75, 118.44, 110.07, 52.40, 40.45, 31.84, 29.36, 29.23, 28.15, 27.16, 22.65, 16.89, 14.10.

HRMS (ESI) m/z Found 365.2223 $(M+H)^+$, calculated 365.2229 for $C_{23}H_{29}N_2O_2^+$.

2.8 Synthesis of NBD-DMA

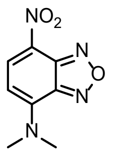

NBD-DMA

The **NBD-DMA** was synthesized according to previous literature reports.[1]

^{1}H NMR (400 MHz, $CDCl_3$) δ 8.45 (d, *J* = 9.0 Hz, 1H), 6.09 (d, *J* = 9.0 Hz, 1H), 3.64 (s, 6H).

2.9 Synthesis of NBD-DEA

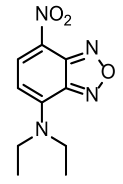

NBD-DEA

The **NBD-DEA** was synthesized according to previous literature reports.[2]

^{1}H NMR (400 MHz, $CDCl_3$) δ 8.41 (d, *J* = 9.1 Hz, 1H), 6.13 (d, *J* = 9.1 Hz, 1H), 3.98 (s, 4H), 1.41 (t, *J* = 7.0 Hz, 6H).

2.10 Synthesis of LD-BFP488

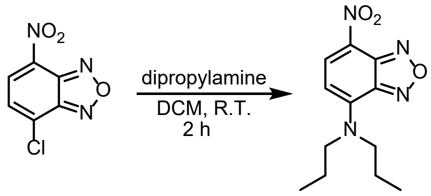

LD-BFP488

Dipropylamine (126 mg, 1.25 mmol) was slowly added into the solution of 4-chloro-7-nitrobenzofurazan (50 mg, 0.25 mmol) in 5 mL DCM. The mixture was stirred for 2 h at room temperature, then the solvent was removed under reduced pressure and the residue was further purified by flash column chromatography (PE:DCM = 1:2) to obtain an orange solid of 51 mg, yield 77%.

^{1}H NMR (400 MHz, $CDCl_3$) δ 8.41 (d, *J* = 9.1 Hz, 1H), 6.10 (d, *J* = 9.1 Hz, 1H), 3.85 (s, 4H), 1.81 (dq, *J* = 15.1, 7.4 Hz, 4H), 1.06 (t, *J* = 7.4 Hz, 6H).

^{13}C NMR (100 MHz, $CDCl_3$) δ 145.02, 144.97, 144.40, 135.46, 121.61, 100.89, 55.69, 20.59, 11.13.

HRMS (ESI) m/z Found 265.1310 $(M+H)^+$, calculated 265.1301 for $C_{12}H_{17}N_4O_3^+$.

2.11 Synthesis of BDP-MeS

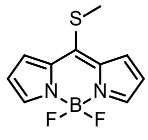

BDP-MeS

The **BDP-MeS** was synthesized according to previous literature reports.[3]

^{1}H NMR (400 MHz, $CDCl_3$) δ 7.80 (s, 2H), 7.42 (d, *J* = 3.8 Hz, 2H), 6.53 (d, *J* = 3.2 Hz, 2H), 2.92 (s, 3H).

2.12 Synthesis of BDP-2MeS

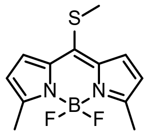

BDP-2MeS

The **BDP-2MeS** was synthesized according to previous literature reports.[4]

^{1}H NMR (400 MHz, $CDCl_3$) δ 7.29 (d, J = 4.1 Hz, 2H), 6.27 (d, J = 4.1 Hz, 2H), 2.71 (s, 3H), 2.60 (s, 6H).

2.13 Synthesis of BDP-4MeS

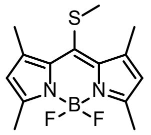

BDP-4MeS

The **BDP-4MeS** was synthesized according to previous literature reports.[5]

^{1}H NMR (400 MHz, $CDCl_3$) δ 6.09 (s, 2H), 2.61 (s, 6H), 2.52 (s, 6H), 2.46 (s, 3H).

2.14 Synthesis of LD-BFP405

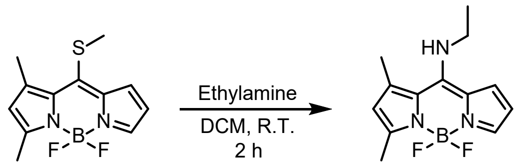

BDP-S **LD-BFP405**

The **BDP-S** was synthesized according to previous literature reports.[3]

Ethylamine (85 mg, 1.9 mmol) was added to the solution of **BDP-S** (50 mg, 0.19 mmol) in 10 mL DCM at room temperature. After 2 h, the solvent was removed under reduced pressure and the residue was further purified by flash column chromatography (DCM) to give a pale solid of 17 mg, yield 35%.

^{1}H NMR (400 MHz, $CDCl_3$) δ 7.57 (s, 1H), 6.98 (s, 1H), 6.45 (s, 1H), 6.14 (s, 1H), 5.94 (s, 1H), 3.68 – 3.55 (m, 2H), 2.49 (s, 3H), 2.33 (s, 3H), 1.43 (t, J = 7.0 Hz, 3H).

^{13}C NMR (100 MHz, $CDCl_3$) δ 148.09, 145.42, 133.21, 130.64, 122.68, 121.99, 120.71, 117.65, 113.86, 42.33, 15.40, 14.70, 13.82.

HRMS (ESI) m/z Found 264.1481 $(M+H)^+$, calculated 264.1484 for $C_{13}H_{17}BF_2N_3^+$.

2.15 Synthesis of LD-BFP543

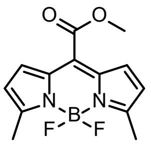

LD-BFP543

The **LD-BFP543** was synthesized according to previous literature reports.[6]

^{1}H NMR (400 MHz, $CDCl_3$) δ 7.22 (d, J = 4.1 Hz, 2H), 6.31 (d, J = 4.2 Hz, 2H), 3.99 (s, 3H), 2.63 (s, 6H).

3. Experimental Procedures

3.1 Spectral measurements in the different solvents

The probe stock solution (2 mmol) were prepared in DMSO. Then, the UV−vis absorption and fluorescence spectra of LD-BFPs were measured in the different solvents (ACN, $CHCl_3$, DMSO, EtOH, water) at a concentration of 5 μM.

Coumarin 153 was used to obtain relative fluorescence quantum yields of compounds (fluorescence quantum yields of Coumarin 153 is 0.53 in ethanol). The quantum yield (φ) was calculated using the following equation.

$$\varphi F_{(X)} = \varphi F_{(S)} \cdot (A_S F_X / A_X F_S)(\eta_X / \eta_S)^2$$

Where φF, A and F represent the fluorescence quantum yield, the absorbance at the excitation wavelength and the area under the corrected emission curve, respectively. And η is the refractive index of the solvent. Subscripts X and S refer to the unknown and to the standard.

3.2 Cell culture

HeLa (helacyton gartleri) cells were purchased from Cell Bank of Type Culture Collection of Chinese Academy of Sciences. HeLa cells were maintained in Dulbecco's modified Eagle's medium (DMEM, Gibco) supplemented with 10% fetal bovine serum (FBS, Hyclone) which were cultured in a humidified atmosphere of 5% CO_2/95% air at 37°C. Before the imaging experiments, HeLa cells were seeded on glass bottom cell culture dish (Nest, polystyrene, Φ 15 mm) for 1-2 days to reach 60-80% confluency. The cells were then used for further experiments.

3.3 Cell transfection

Lipid droplet membrane plasmid pEFIRES-P-ACSL3-mCherry was purchased from addgene. Transfection experiment was performed according Lipofectamine 2000 (Invitrogen) according to the manufacturer's protocol.

Briefly, 1.5 μL Lipofectamine 2000 (Invitrogen) and appropriate plasmid were firstly diluted in 20 μL DMEM (dulbecco's modified eagle medium), respectively. 5 min later, the diluted plasmid in 20 μL DMEM was added to the diluted Lipofectamine 2000 (Invitrogen) with homogeneous mixing. Another 10 min later, the mixture was added to the cell-culture dish in 1 mL DMEM. The final concentration of plasmid was controlled at 500-1000 ng/mL. After incubated 4 h in 37 °C, the culture medium was changed from DMEM to DMEM with 10% FBS. 24 - 48 h later, the transfected cells were used for imaging.

3.4 Cell toxicity assay

The cytotoxicity of **LD-BFPs** was detected by MTT assays. Hela Cells were seeded into 96-well plates with 1 × 10^4 cells/well and cultured for 12 h. After the adding of various probe (5 μM), the cells were incubated for 24 h at 37 ºC with 5% CO_2 atmosphere. Then, the medium was replaced with fresh 100 μL medium and 10 μL of 5 mg/mL MTT solution per well. After incubating the cells for 4 h, the medium was removed out carefully, and 150 μL DMSO was added to each well to dissolve blue formazan. Finally, the absorption was recorded at 570 nm using a UV-Vis microplate reader.

3.5 Imaging

3.5.1 Confocal imaging of HeLa cells

For imaging lipid droplet in living HeLa cells, the experimental group was incubated with 2 μM LD-BFPs in DMEM for 30 min at 37°C, then directly used for imaging. In order to confirm the specificity of probes for lipid droplets, HeLa cells were co-stained with corresponding commercial LD dyes (BODIPY 493 or LD 540) at 500 nM. Microscopic images of the cells were obtained on a live-cell confocal laser scanning microscope without wash-out steps.

3.5.2 FRAP imaging

Fluorescence recovery after photobleaching (FRAP) experiments were carried out on Olympus FV1000 MPE confocal laser scanning microscope. Two images were taken before the bleach pulse and 13 images after the bleaching of regions of interest (ROIs) at 10% laser transmission to minimize scan bleaching. The imaging interval is about 3 seconds. For photobleaching process, the ROIs were bleached by 100% 488 nm laser for 10 seconds. The fluorescence intensity was acquired by the software in imaging system. The ± s.d. from at least 5 FRAP experiments each.

3.5.3 SIM imaging

For imaging lipid droplet in living HeLa cells by SIM, the experimental group was incubated with 2 μM LD-BFPs in DMEM for 30 min at 37°C, then directly used for imaging.

To verify the photostability of LD-BFPs in living cells, the imaging interval was 5 s with the 15 min total imaging time. (Figure S10)

To verify the high spatial resolution of LD imaging, the HeLa cells were pretreated with 200 μM metformin for 12 h to reduce the LD diameter. Then the HeLa cells were stained by 2 μM LD-BFP488 for 30 min and directly used for imaging. (Figure S11)

4. Spectroscopic characterizations of LD-BFPs

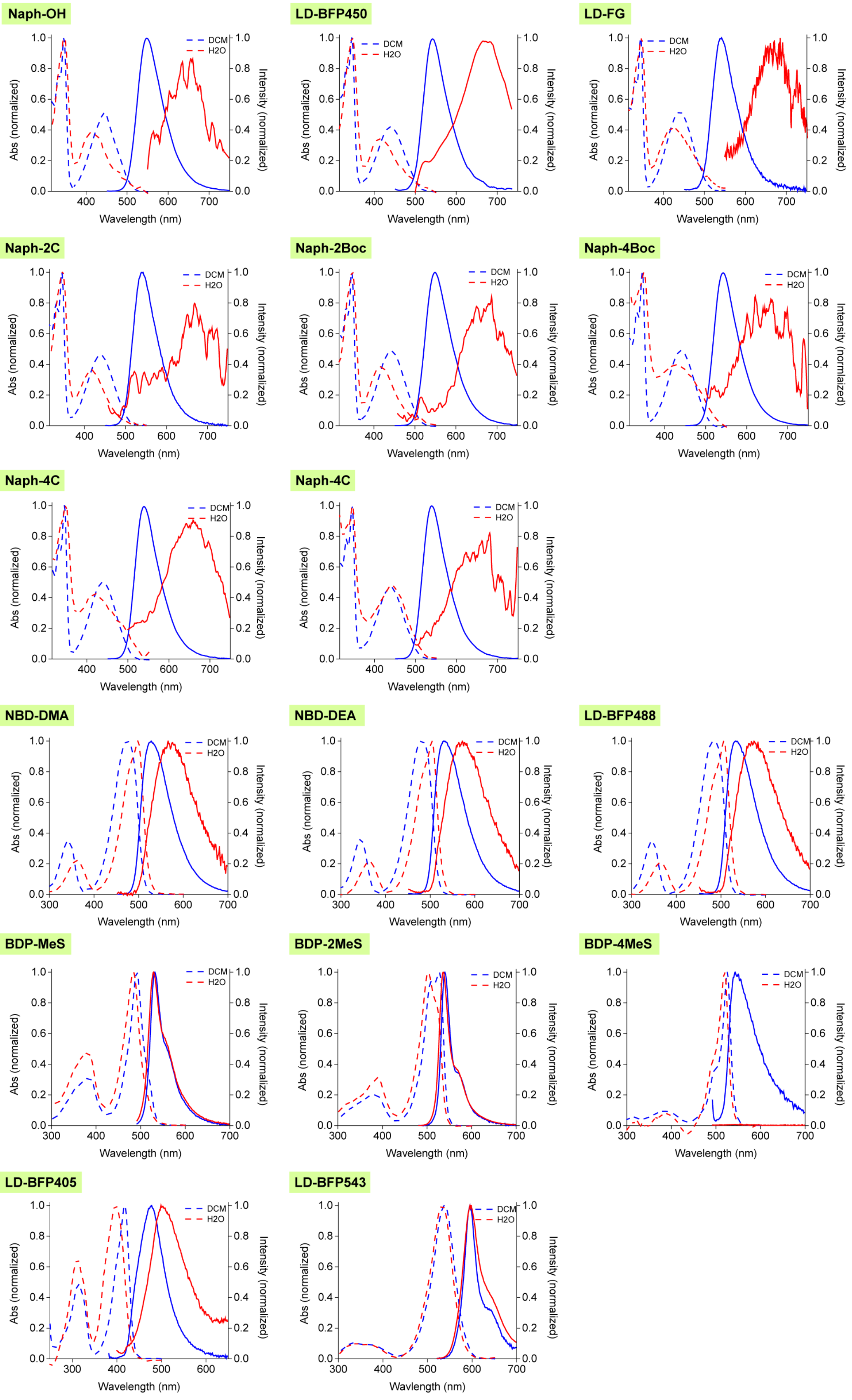

Figure S1. UV-Vis absorption spectrum (dotted line) and fluorescence emission spectrum (solid line) of 5 μM LD-BFPs in dichloromethane (DCM) and water (H_2O).

Table S1. Photophysical data for LD-BFPs in dichloromethane (top line) and water (bottom line): peak UV-vis absorption wavelength (λ_{abs}), maximum emission wavelength (λ_{em}), Stokes shifts, molar absorption coefficient (ε), fluorescence quantum yield (φ) and brightness.

probe	λ_{abs} (nm)	λ_{em} (nm)	stokes shift (nm)	ε ($M^{-1}\cdot cm^{-1}$)	φ	brightness
**Naph-OH**	444	549	105	4201	0.63	2646
	413	645	232	3054	<0.01	31
**LD-BFP450**	437	544	107	3899	0.50	1950
	413	674	261	3360	<0.01	34
**LD-FG**	442	542	100	3604	0.70	2523
	415	669	254	3250	<0.01	33
**Naph-2C**	438	541	103	4517	0.63	4517
	416	655	239	2557	0.01	26
**Naph-2Boc**	441	549	108	4477	0.62	2846
	417	669	252	3450	0.01	35
**Naph-4Boc**	439	543	104	4373	0.71	3105
	431	608	177	2499	0.01	25
**Naph-4C**	439	538	99	4446	0.68	3023
	422	613	191	2046	0.02	41
**Naph-8C**	440	542	102	4014	0.66	2649
	443	613	170	3200	0.01	32
**NBD-DMA**	475	528	53	30612	0.21	6429
	498	566	68	40912	0.01	409
**NBD-DEA**	482	530	48	28910	0.04	1156
	504	572	68	40926	<0.01	409
**LD-BFP488**	485	542	57	30210	0.04	1208
	507	573	66	43558	<0.01	436
**BDP-MeS**	491	532	41	42084	0.44	18517
	484	528	44	26106	0.09	2350
**BDP-2MeS**	527	540	13	43604	0.80	34883
	503	537	34	27188	0.14	3806
**BDP-4MeS**	525	543	18	66140	0.01	661
	519	543	24	11434	<0.01	114
**LD-BFP405**	418	478	60	41028	0.17	6975
	396	499	103	19566	0.07	1370
**LD-BFP543**	539	595	56	36834	0.66	24310
	533	597	64	32926	0.27	8890

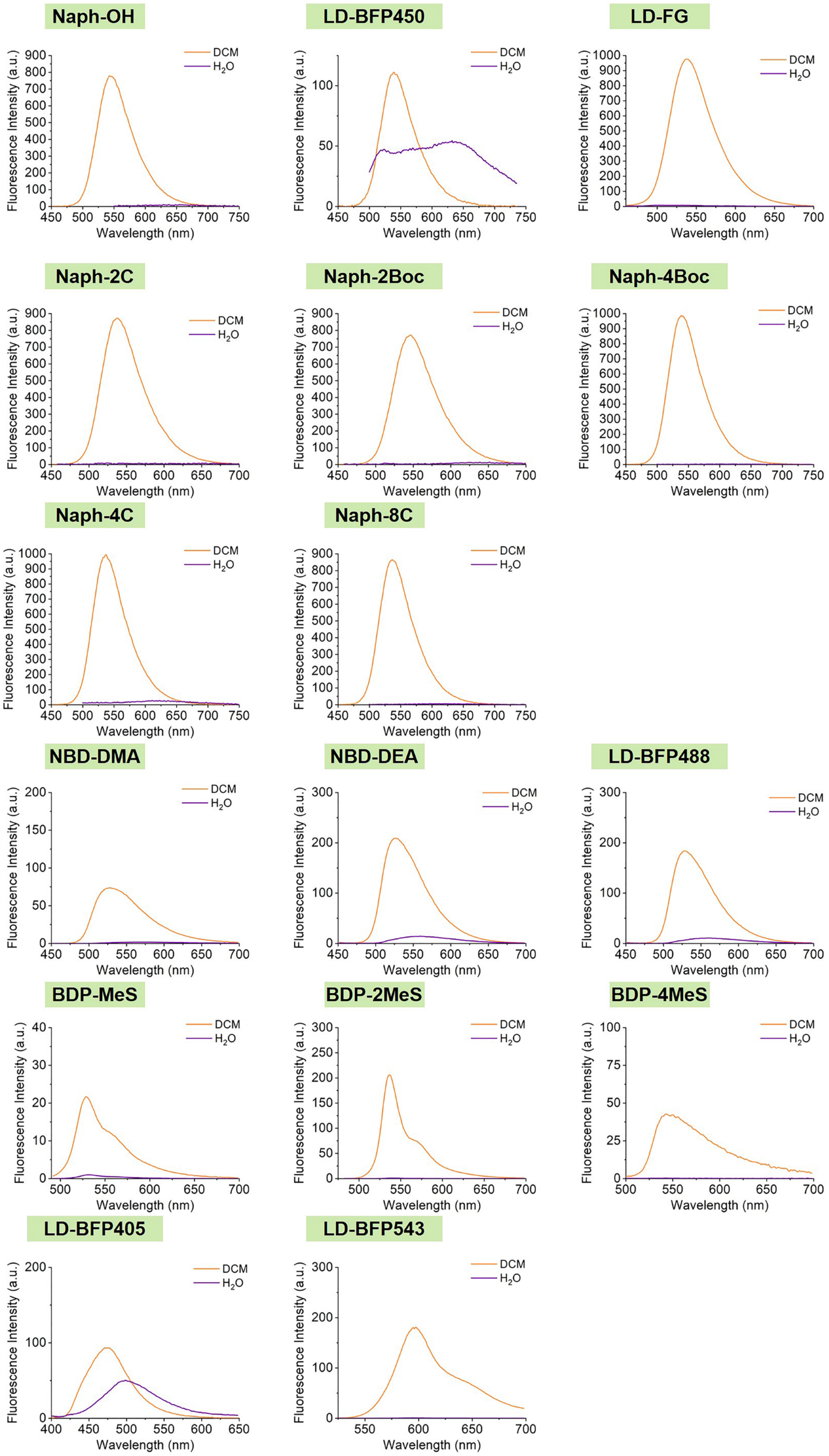

Figure S2. Fluorescence emission spectrum of 5 μM LD-BFPs in dichloromethane (DCM) and water (H_2O).

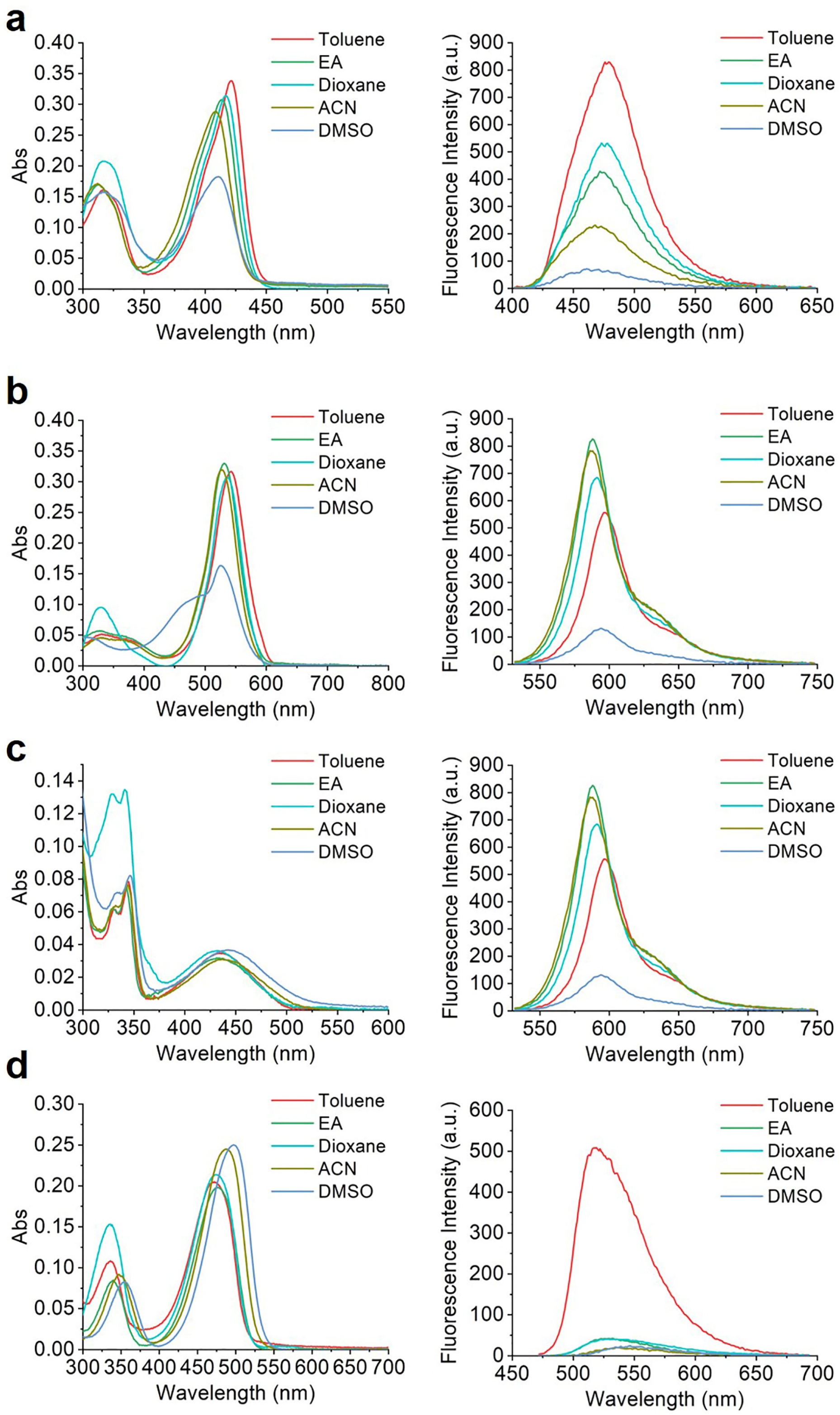

Figure S3 UV-Vis absorption spectrum and fluorescence emission spectrum of 10 μM LD-BFPs different solvents. (a) **LD-BFP405**, (b) **LD-BFP543**. (c) **LD-BFP450** and (d) **LD-BFP488**.

Table S2. Photophysical data for LD-BFPs in different solvents. peak UV-vis absorption wavelength (λ_{abs}), maximum emission wavelength (λ_{em}), Stokes shifts, molar absorption coefficient (ε), fluorescence quantum yield (φ) and brightness.

probe	Solvent	λ_{abs} (nm)	λ_{em} (nm)	stokes shift (nm)	ε ($M^{-1}cm^{-1}$)	φ	brightness
**LD-BFP405**	Toluene	422	479	57	33771	0.26	8735
	EA	414	472	58	30739	0.07	2388
	Dioxane	417	473	56	31420	0.12	3973
	ACN	408	468	60	28834	0.04	1068
	DMSO	411	461	50	18224	0.02	400
**LD-BFP543**	Toluene	542	594	52	31648	0.32	13099
	EA	532	588	56	32962	0.33	11909
	Dioxane	537	591	54	30678	0.31	12270
	ACN	527	586	59	28842	0.29	9776
	DMSO	526	593	70	31872	0.32	4859
**LD-BFP450**	Toluene	437	518	81	3487	0.62	2168
	EA	431	549	116	3181	0.49	1552
	Dioxane	432	528	96	3627	0.62	2257
	ACN	437	573	136	3109	0.30	934
	DMSO	442	597	155	3665	0.26	953
**LD-BFP488**	Toluene	471	519	48	20493	0.27	5494
	EA	477	532	55	19805	0.02	433
	Dioxane	474	522	48	21387	0.08	1772
	ACN	489	535	46	24467	0.01	258
	DMSO	497	547	50	25004	0.02	539

5 Fluorescent imaging of LD-BFPs in living cells

5.1 Colocalization experiments of LD-BFPs with commercial LD dyes in living cells

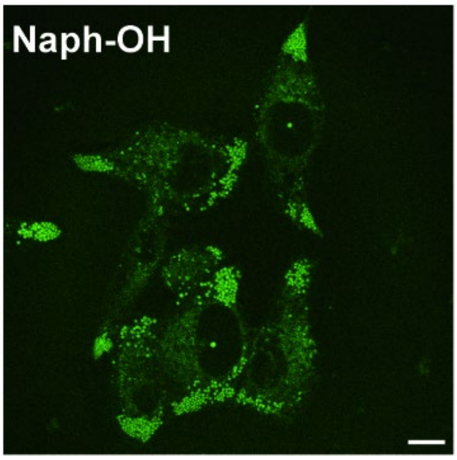

Figure S4. Confocal images of living HeLa cells incubated with 2 μM **Naph-OH** for 60 min. Scale bar = 10 μm.

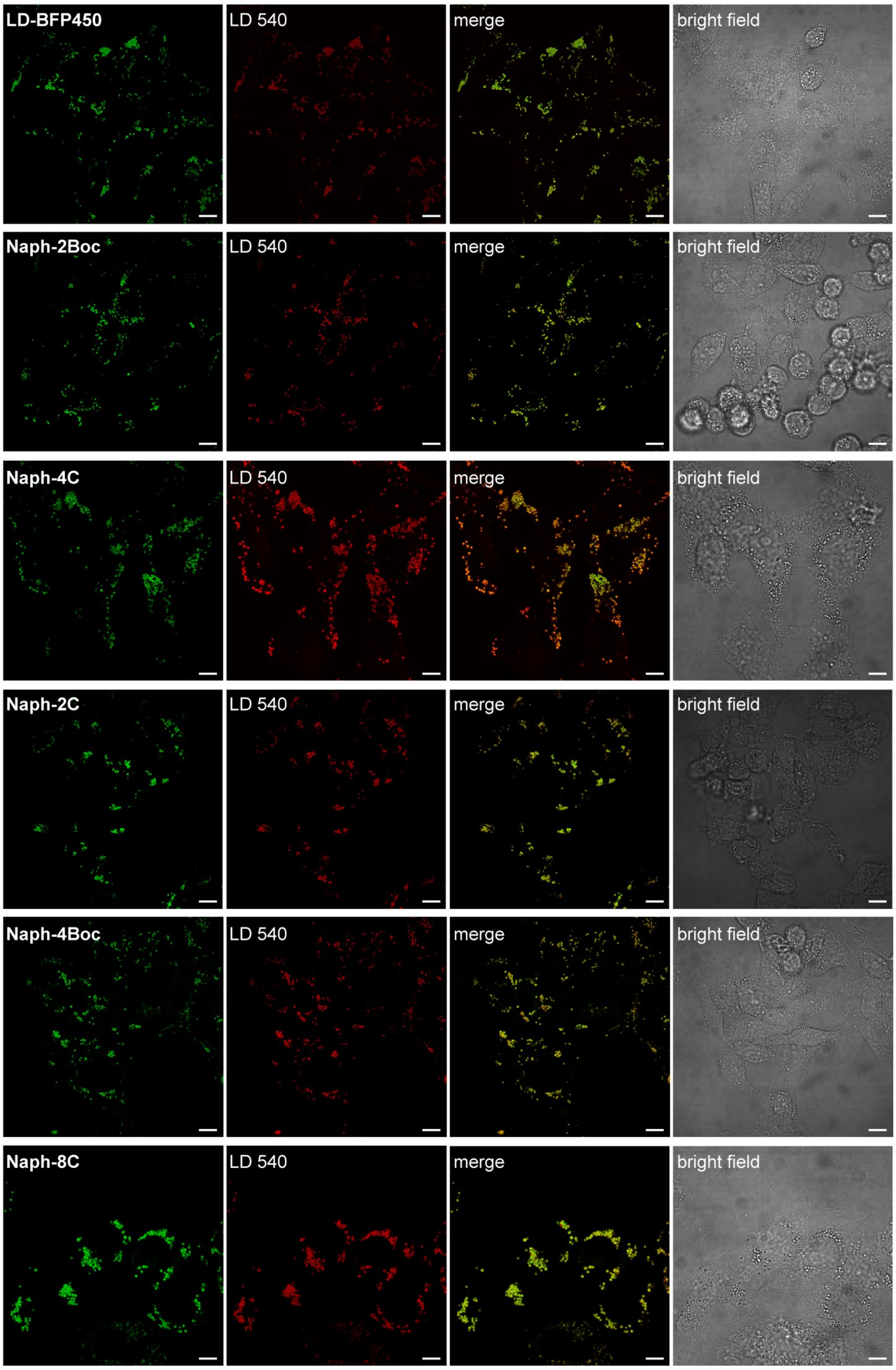
LD-BFP450
LD 540
merge
bright field
Naph-2Boc
LD 540
merge
bright field
Naph-4C
LD 540
merge
bright field
Naph-2C
LD 540
merge
bright field
Naph-4Boc
LD 540
merge
bright field
Naph-8C
LD 540
merge
bright field

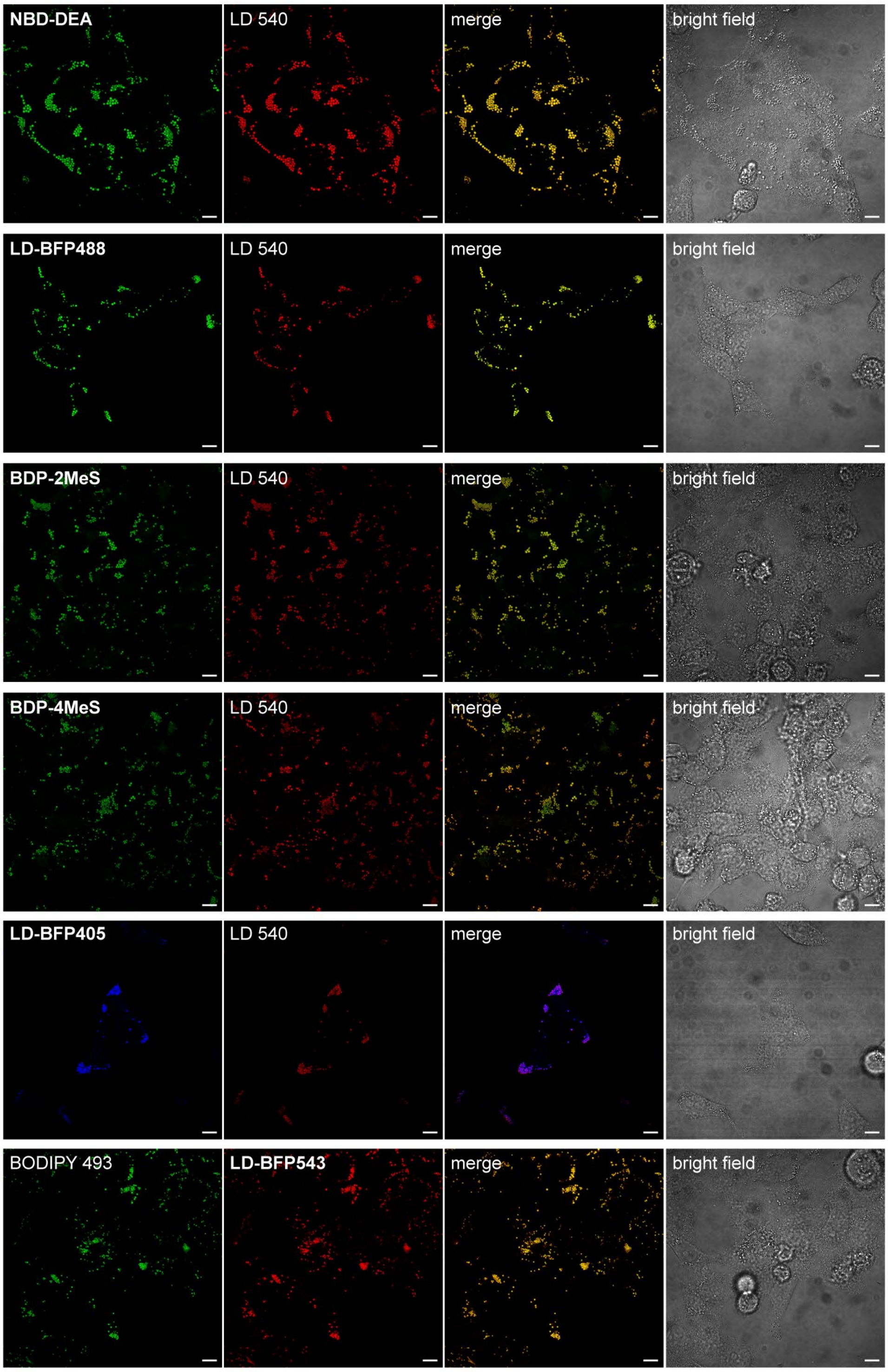

Figure S5. Colocalization experiments of LD-BFPs in living HeLa cells. Dual color imaging of living HeLa cells incubated with 2 μM LD-BFPs and 500 nM corresponding commercial LD dyes for 30 min. Scale bar = 10 μm.

5.2 FRAP experiments of Naph-LD-BFPs in living HeLa cells

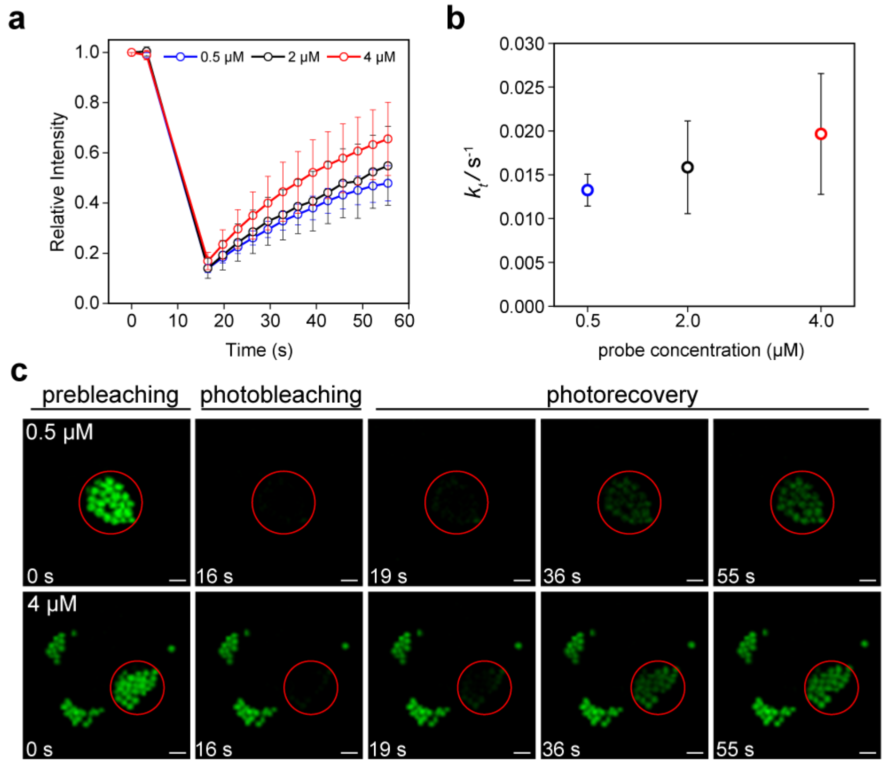

Figure S6. FRAP experiments performed on HeLa cells incubated with different concentration **Naph-4Boc**. (a) Fluorescent intensity changes of photobleaching area during FRAP experiments. (b) The photorecovery rate k_t at initial stage of FRAP experiment. (c) The confocal images of different concentration **Naph-4Boc** in FRAP experiments. The red circle represented the bleaching area. Scale bar = 2 µm.

5.3 FRAP experiments of NBD-LD-BFPs in living HeLa cells

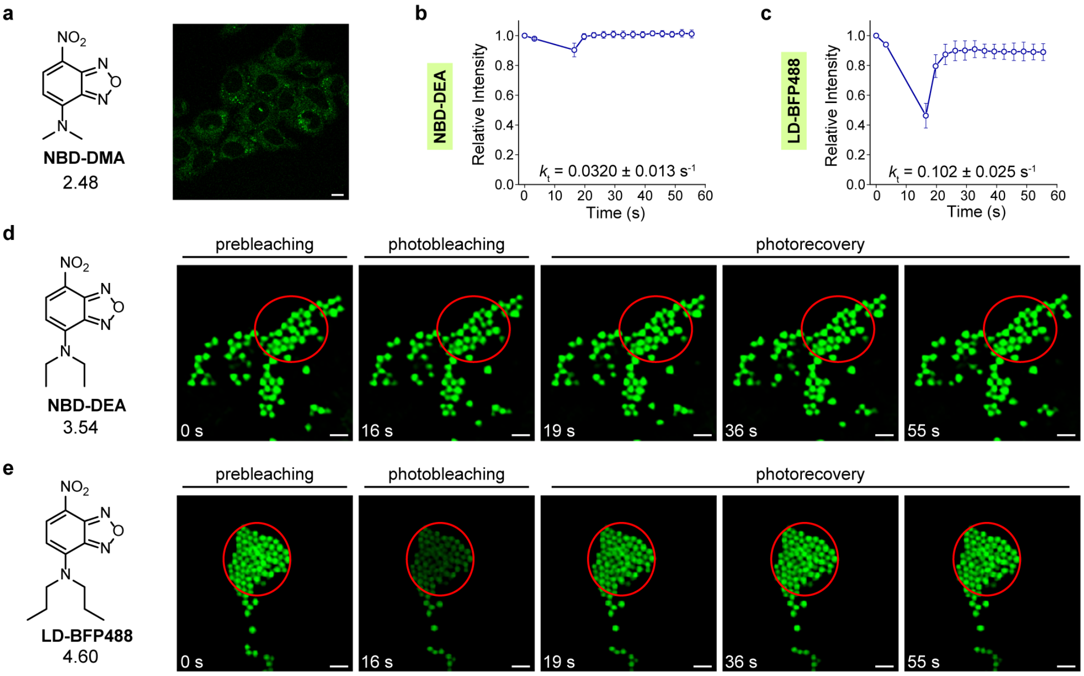

Figure S7. FRAP experiments of NBD-LD-BFPs in HeLa cells. (a) Confocal image of living HeLa cells incubated with 2 μM **NBD-DMA** for 60 min. Scale bar = 10 μm. (b) (c) Fluorescent intensity changes and photorecovery rate k_t of photobleaching area during FRAP experiments. (d) (e) The confocal images of living HeLa cells stained with 2 μM **NBD-DMA** and 2 μM **LD-BFP488** respectively in FRAP experiments. The cells were incubated with the probe for 30 min. The red circle represented the bleaching area. Scale bar = 2 μm.

5.4 FRAP experiments of BDP-LD-BFPs in living HeLa cells

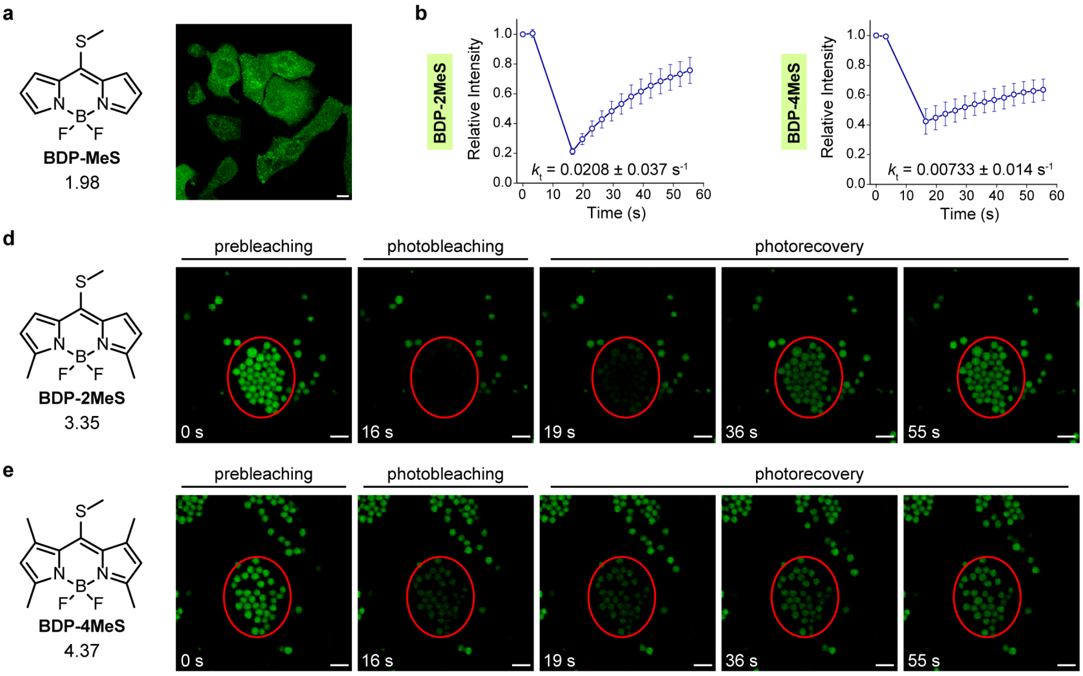

Figure S8. FRAP experiments of BDP-LD-BFPs in HeLa cells. (a) Confocal image of living HeLa cells incubated with 2 μM **BDP-MeS** for 60 min. Scale bar = 10 μm. (b) (c) Fluorescent intensity changes and photorecovery rate k_t of photobleaching area during FRAP experiments. (d) (e) The confocal images of living HeLa cells stained with 2 μM **BDP-2MeS** and 2 μM **BDP-4MeS** respectively in FRAP experiments. The cells were incubated with the probe for 30 min. The red circle represented the bleaching area. Scale bar = 2 μm.

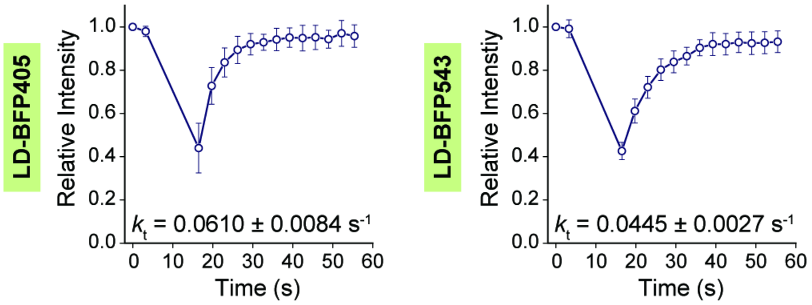

Figure S9. Fluorescent intensity changes and photorecovery rate k_t of photobleaching area during FRAP experiments performed on living HeLa cells incubated with 2 μM **LD-BFP405** and 2 μM **LD-BFP543** respectively for 30 min.

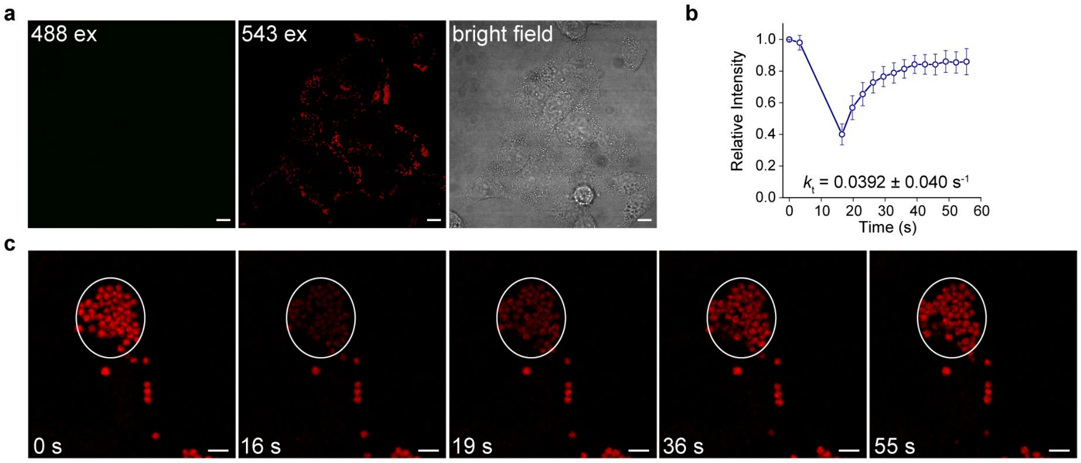

Figure S10. (a) Dual color imaging of living HeLa cells after incubating with 2 μM **LD-BFP543** for 5 h. Scale bar = 10 μm. (b) Fluorescent intensity changes and photorecovery rate k_t of photobleaching area during FRAP experiments performed on living HeLa cells after incubating with 2 μM **LD-BFP543** for 5 h. (c) The confocal images of living HeLa cells after incubating with 2 μM **LD-BFP543** for 5 h in FRAP experiments. The white circle represented the bleaching area. Scale bar = 2 μm.

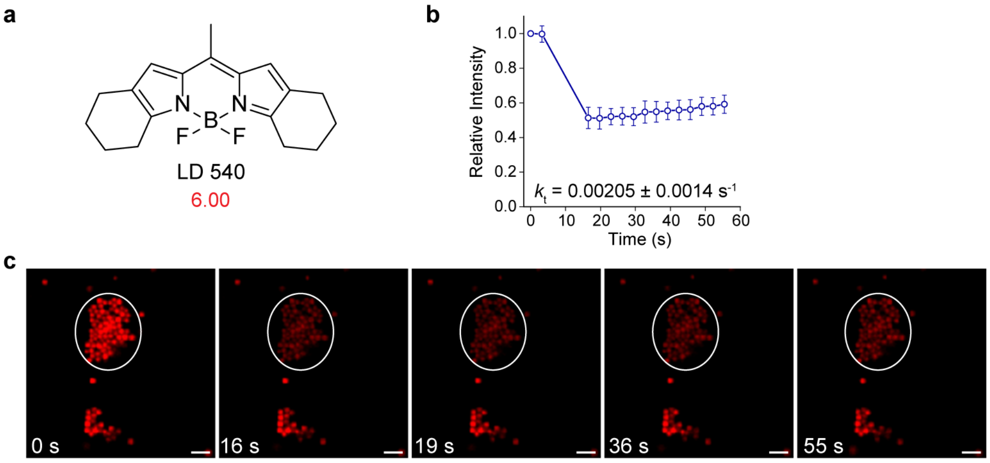

Figure S11. (a) The chemical structure and Clog *P* value of LD 540. (b) Fluorescent intensity changes and photorecovery rate k_t of photobleaching area during FRAP experiments performed on living HeLa cells after incubating with 2 μM LD 540 for 30 min. (c) The confocal images of living HeLa cells after incubating with 2 μM LD 540 for 30 min in FRAP experiments. The white circle represented the bleaching area. Scale bar = 2 μm.

6. Super-resolution imaging of LD dynamics

6.1 Photostability of LD-BFPs in living HeLa cells

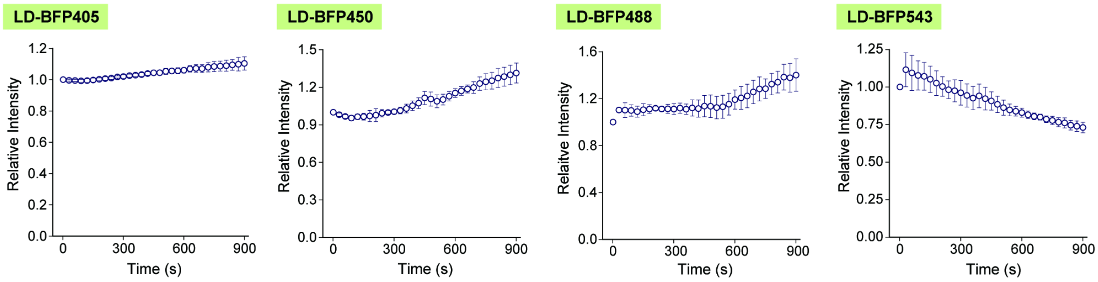

Figure S12. Relative intensity changes of multicolor LD-BFPs in living HeLa cells during SIM imaging. Each data point skiped 6 data pointes. The imaging interval was 5 s within 15 min. The cells were incubated with 2 μM corresponding probe for 30 min.

6.2 The spatial resolution of LD-BFP488 in living HeLa cells

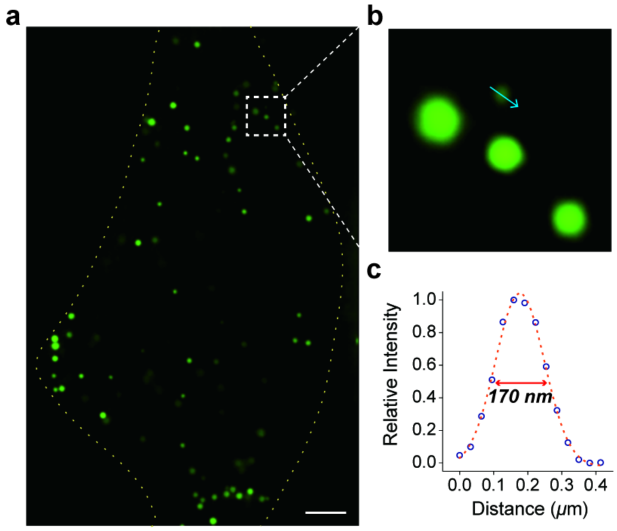

Figure S13. (a) SIM image of living HeLa cell incubated with 2 μM **LD-BFP488** for 30 min. The cells were pretreated with 200 μM metformin for 12 h. Scale bar = 2.5 μm. (b) Enlarge image of white boxed region in (a). (c) The plot profile of blue arrow in (b), the spatial resolution of the image obtained by Gaussian fitting is 170 nm.

6.3 Biocompatibility test of multicolor LD-BFPs in living HeLa cells

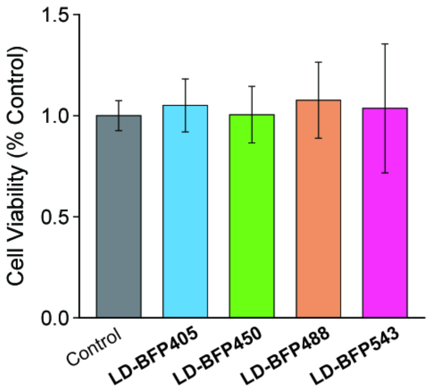

Figure S14. Toxity test of multicolor LD-BFPs with 5 μM for 24 h in living HeLa cells.

6.4 Super-resolution imaging of LD dynamics

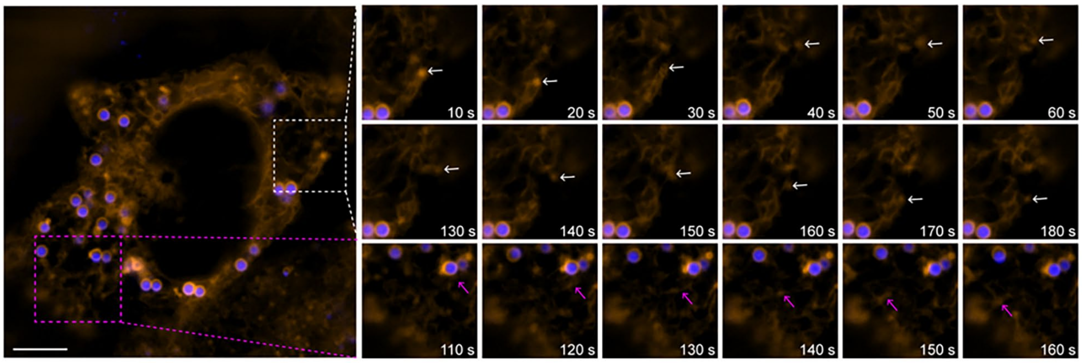

Figure S15. Time lapse SIM images of ACSL3-mcherry vesicles (orange) transport in living HeLa cells stained by 2 μM **LD-BFP405** (blue). The HeLa cells were pretreated with 10 μM sorafenib for 3 h to induce ferroptosis. The imaging interval was 10 s. Scale bar = 5 μm.

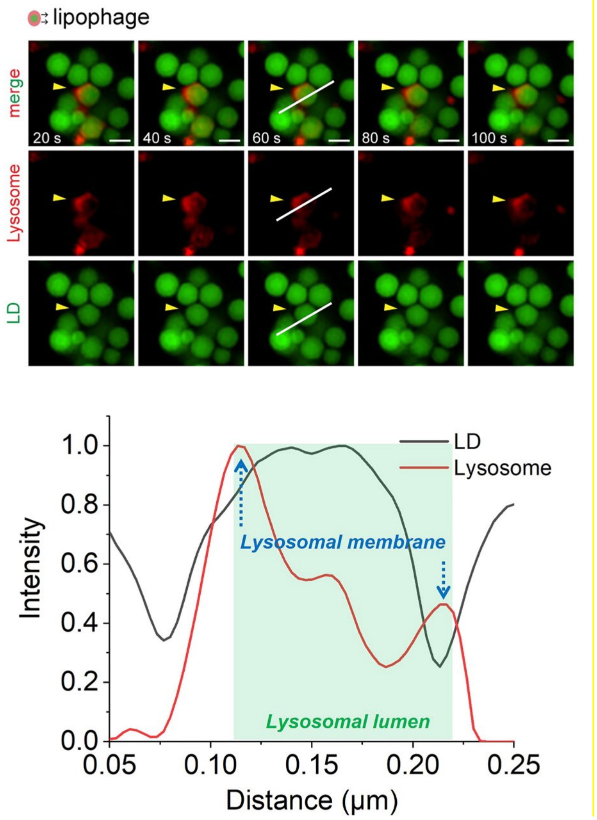

Figure S16. The dynamic lipophagy process and intensity distributions across the white line at 60 s.

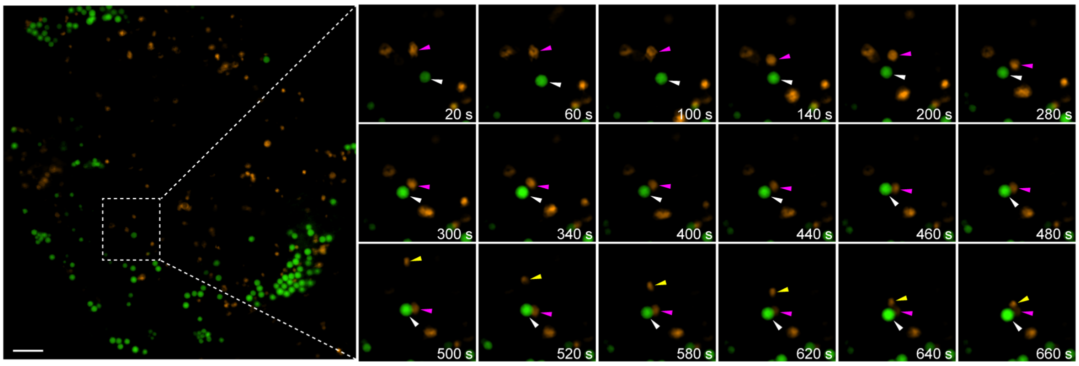

Figure S17. Time-lapse SIM image of HeLa cells and the enlarge images of LD-Lysosome contacts in boxed region during 10 minutes. The imaging interval was 20 s. The HeLa cell stained with 1 μM **LD-BFP450** (green) and 1 μM Lyso-probe (orange). The Lyso-probe was a novel fluorogenic probe synthesized by our lab used for specific lysosome labeling. Scale bar = 5 μm.

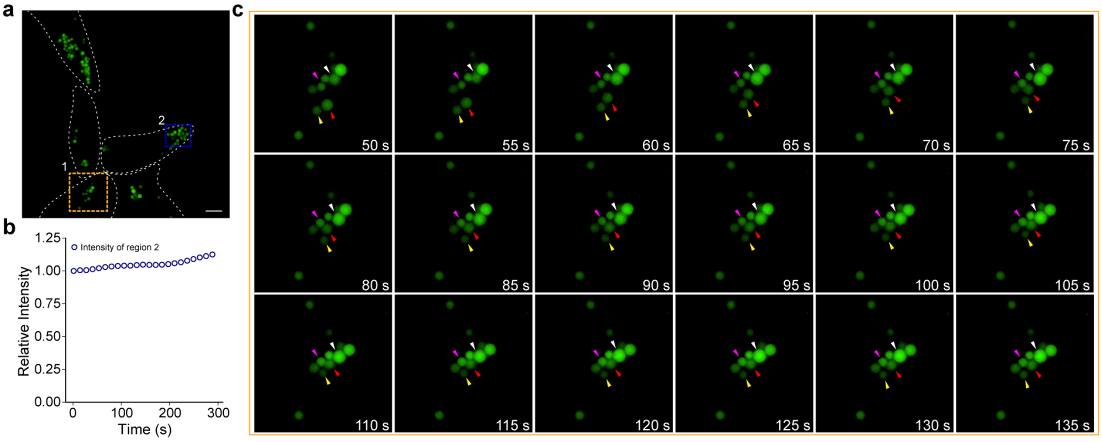

Figure S18. The recruitment of large LD cluster to small LD cluster. (a) SIM images of whole cells incubated with 2 μM **LD-BFP450**. (b) The relative intensity changes of boxed region 2 in (a) during 300 s. Each data point skiped 5 data pointes. The imaging interval was 2.5 s. (c) Time-lapse SIM images of in region 1 in (a) during 50-135 s. Scale bar = 5 μm.

7. Spectra characterization

7.1 ^{1}H-NMR, ^{13}C-NMR, HRMS spectra of intermediate and LD-BFPs

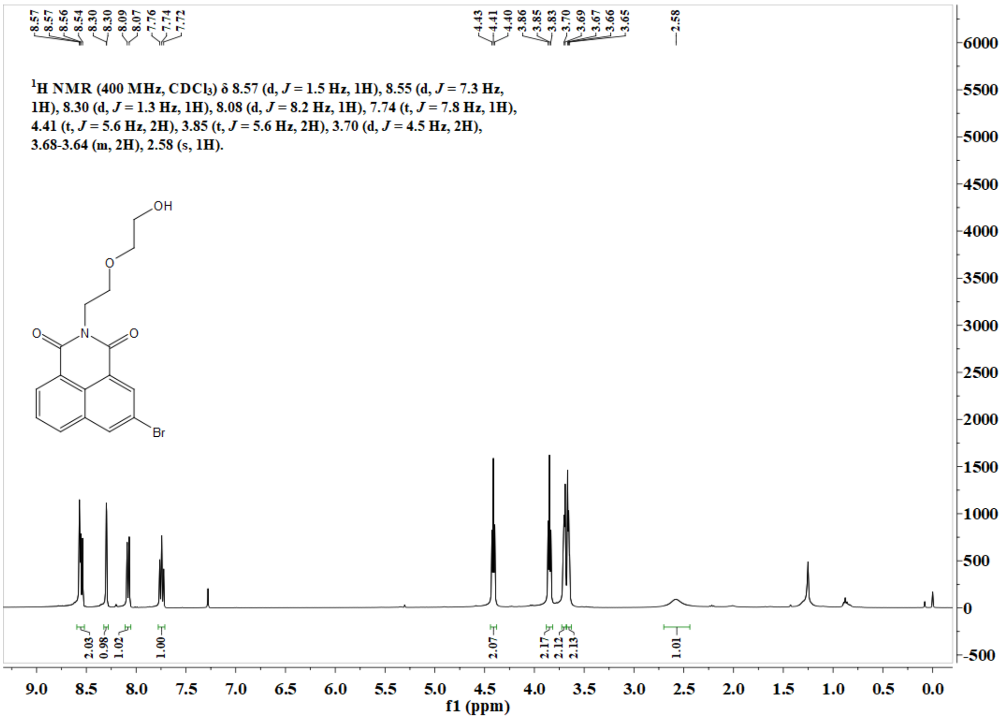

Figure S19. ^{1}H-NMR spectra of **OH-Br** in $CDCl_3$.

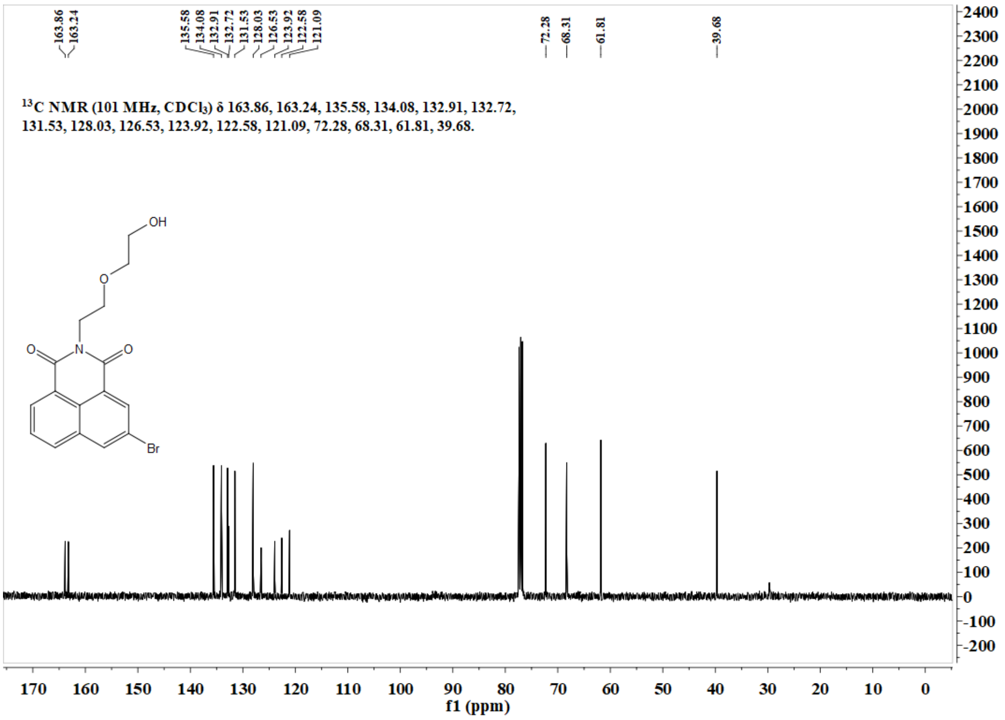

Figure S20. ^{13}C-NMR spectra of **OH-Br** in $CDCl_3$.

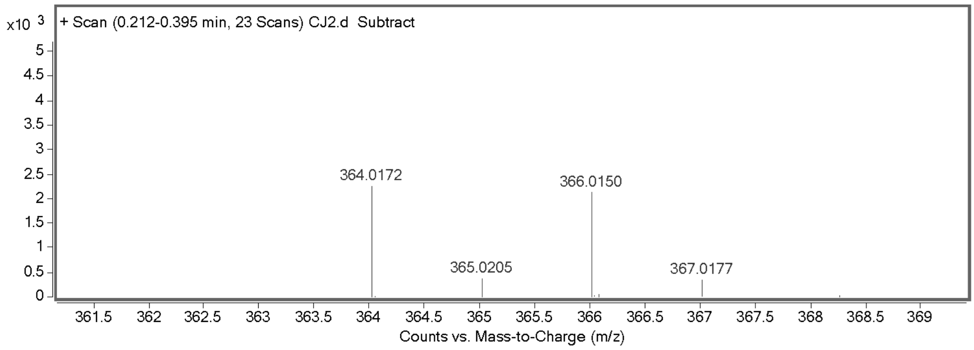

Figure S21. HRMS spectra of **OH-Br**.

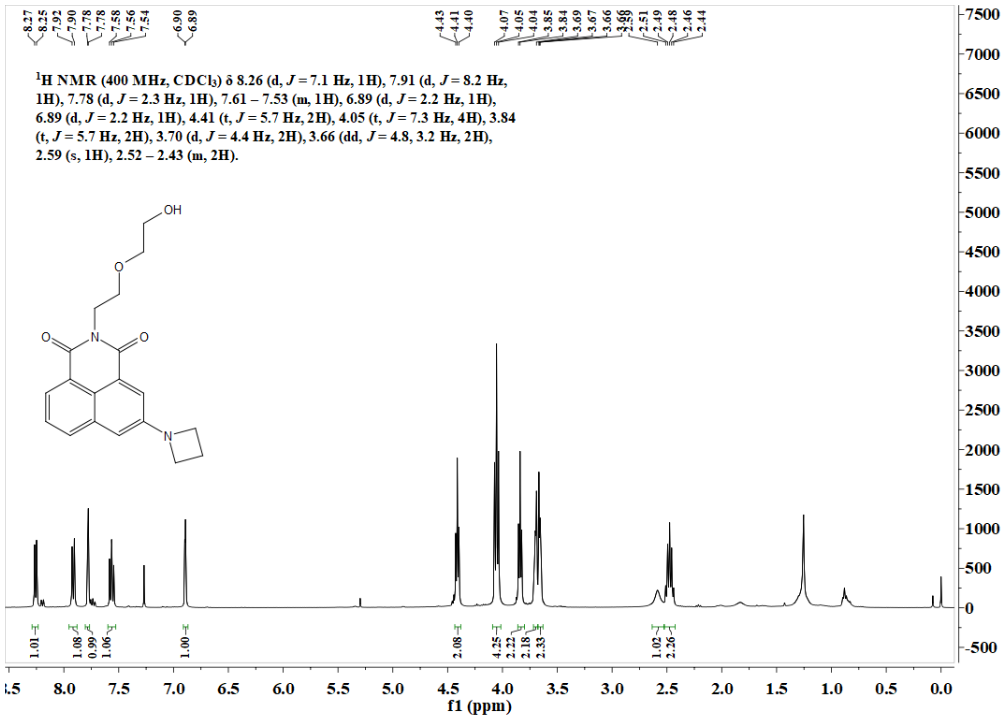

Figure S22. ^{1}H-NMR spectra of **Naph-OH** in $CDCl_3$.

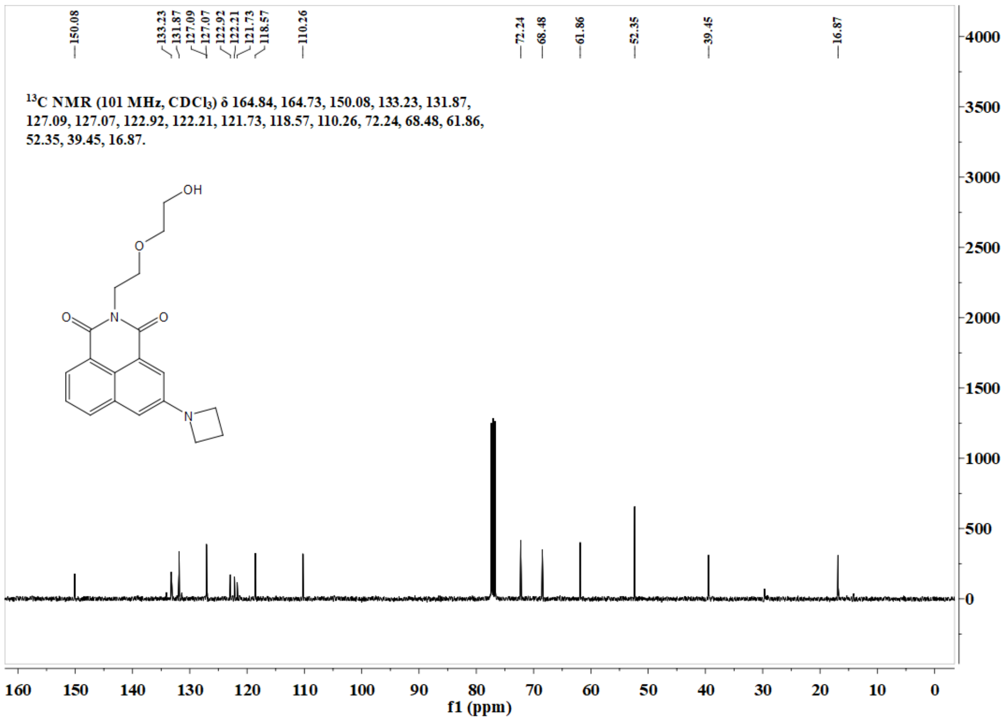

Figure S23. ^{13}C-NMR spectra of **Naph-OH** in CDCl$_3$.

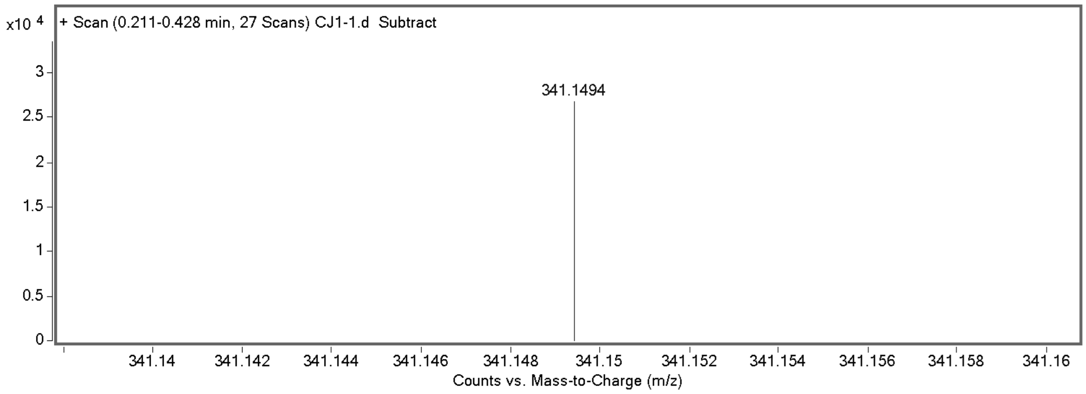

Figure S24. HRMS spectra of **Naph-OH**.

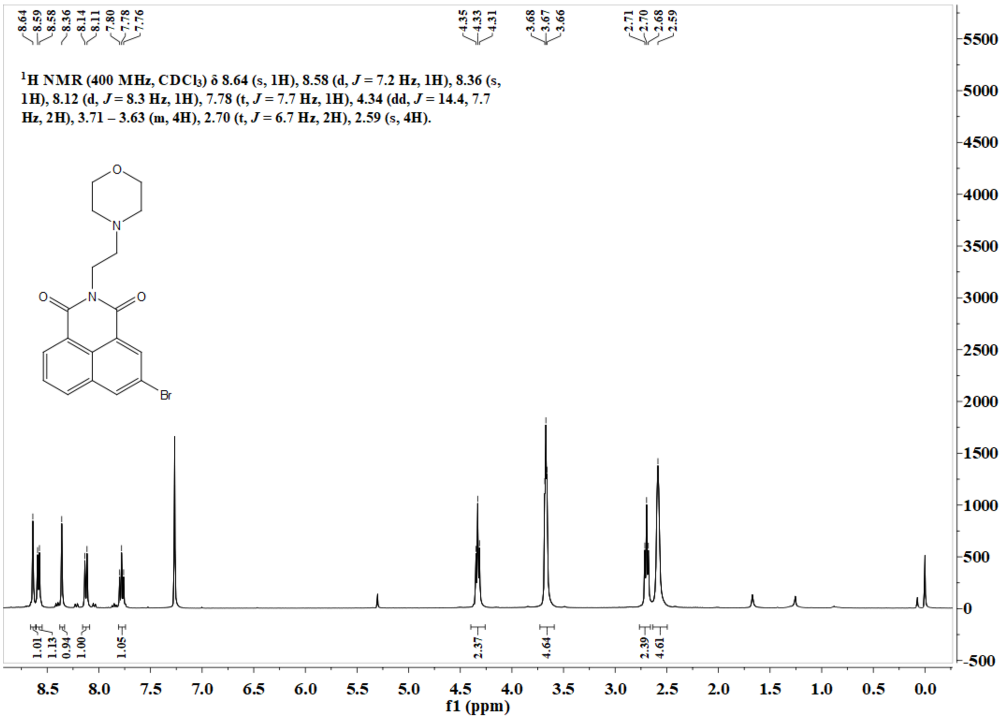

Figure S25. ^{1}H-NMR spectra of **Mor-Br** in $CDCl_3$.

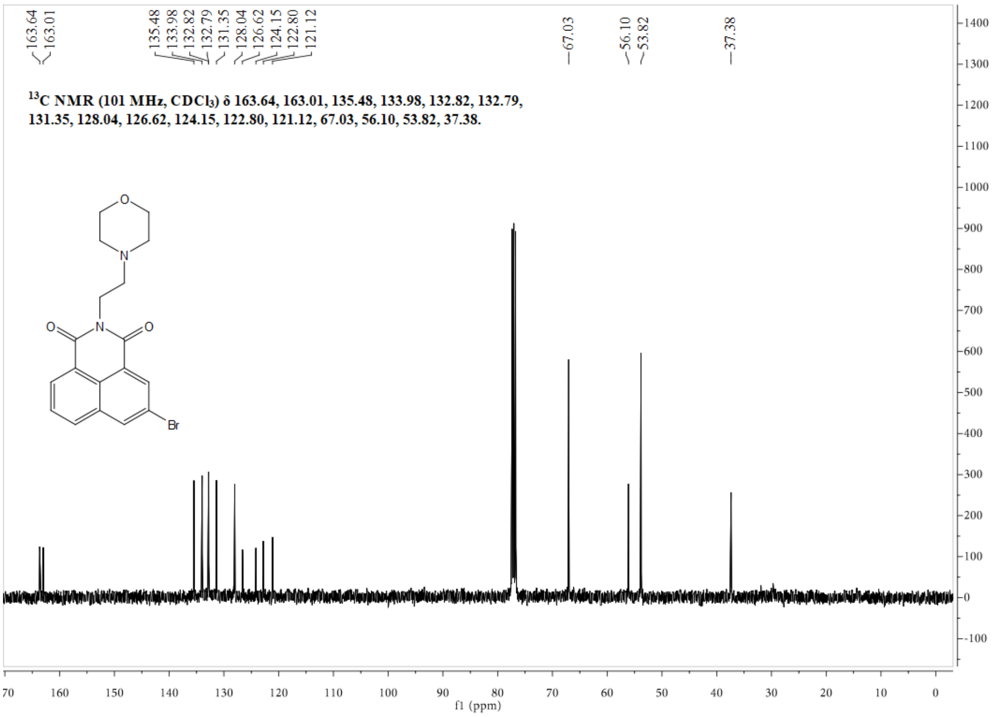

Figure S26. ^{13}C-NMR spectra of **Mor-Br** in $CDCl_3$.

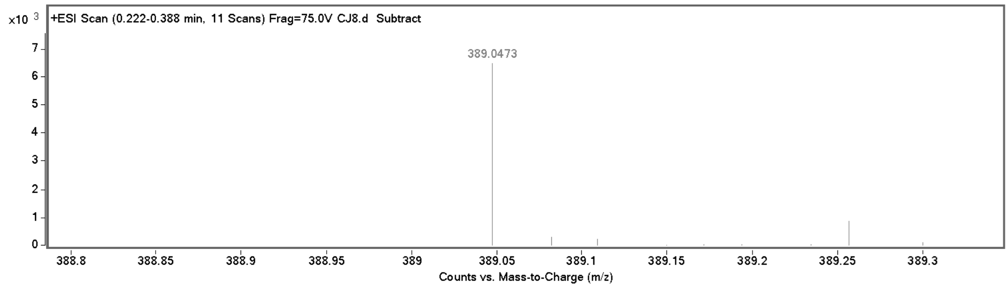

Figure S27. HRMS spectra of **Mor-Br**.

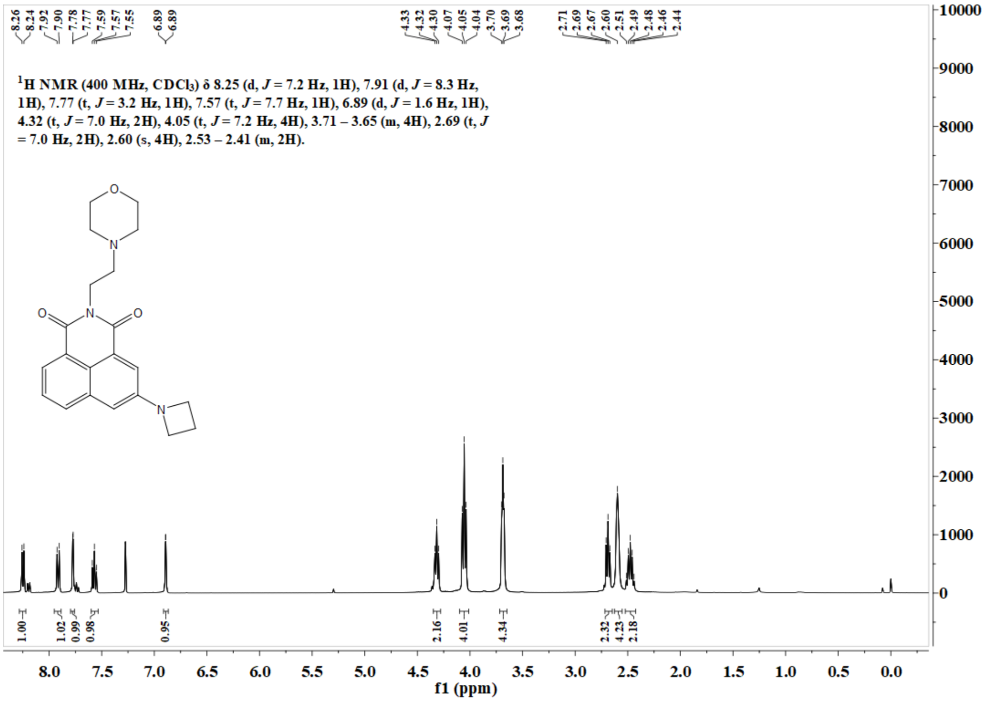

Figure S28. ^{1}H-NMR spectra of **LD-BFP450** in $CDCl_3$.

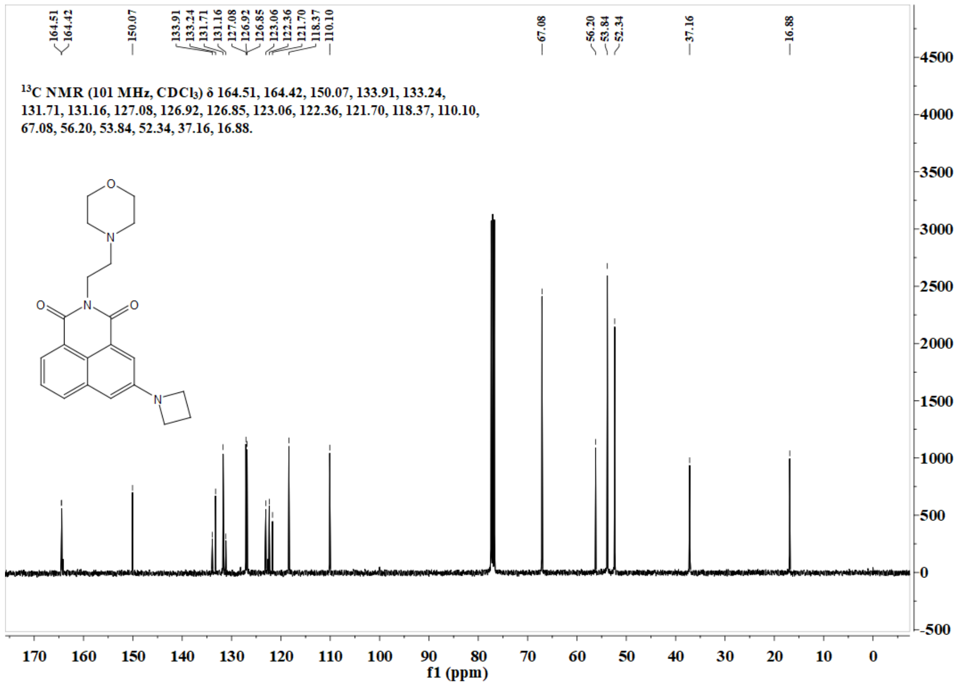

Figure S29. ^{13}C-NMR spectra of **LD-BFP450** in $CDCl_3$.

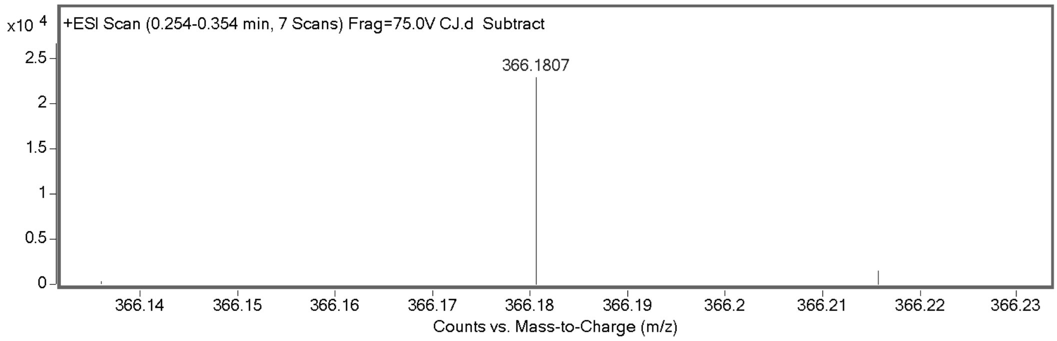

Figure S30. HRMS spectra of **LD-BFP450**.

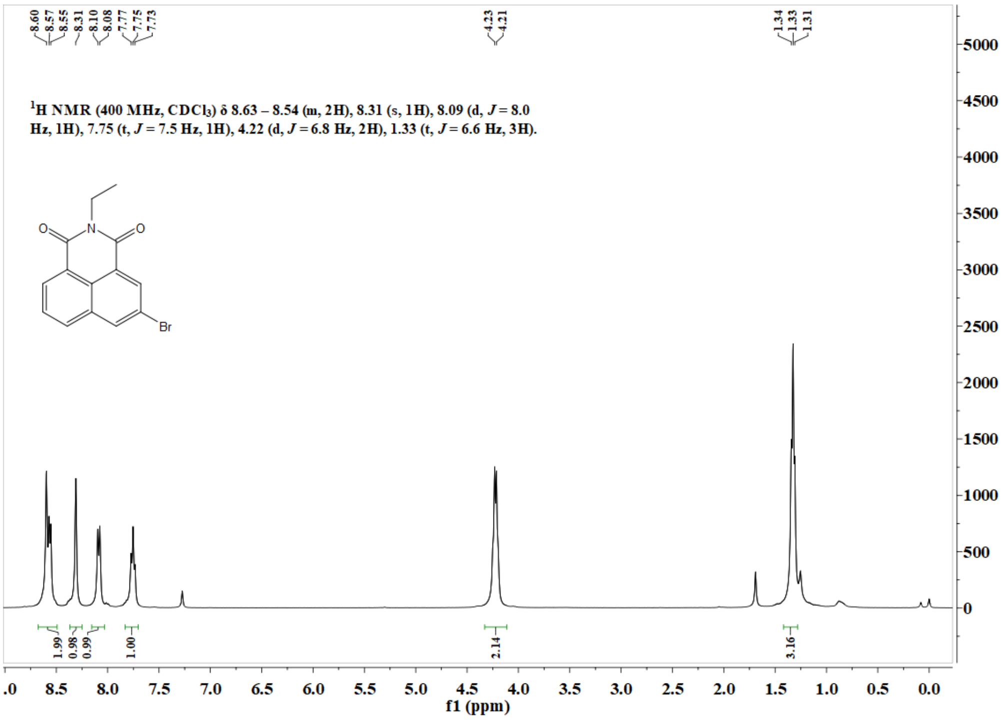

Figure S31. ^{1}H-NMR spectra of **2C-Br** in $CDCl_3$.

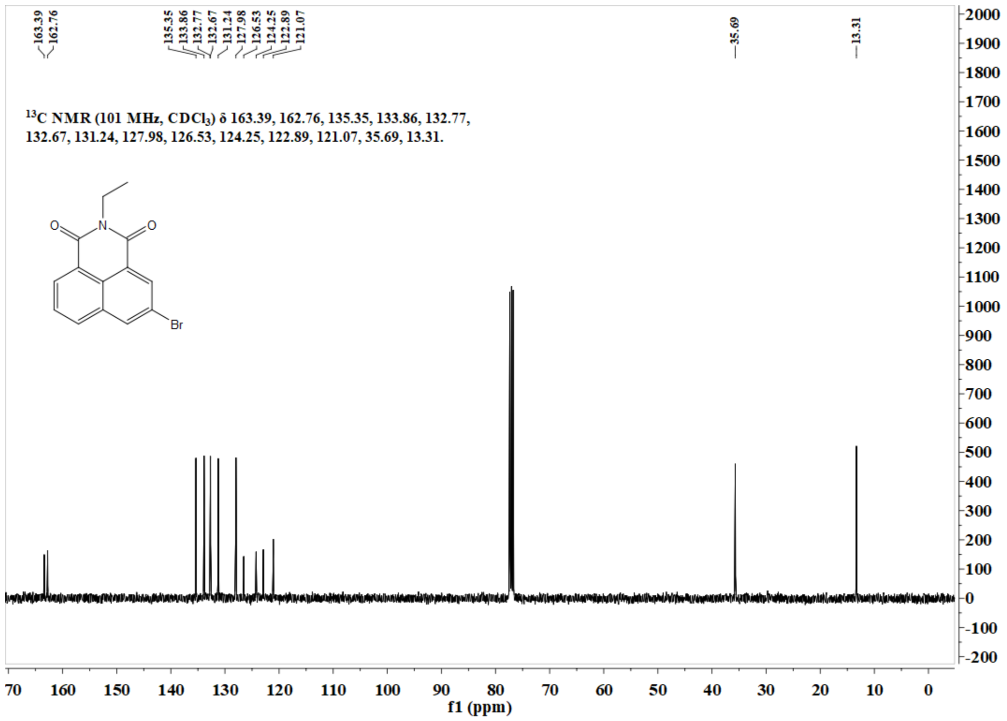

Figure S32. ^{13}C-NMR spectra of **2C-Br** in $CDCl_3$.

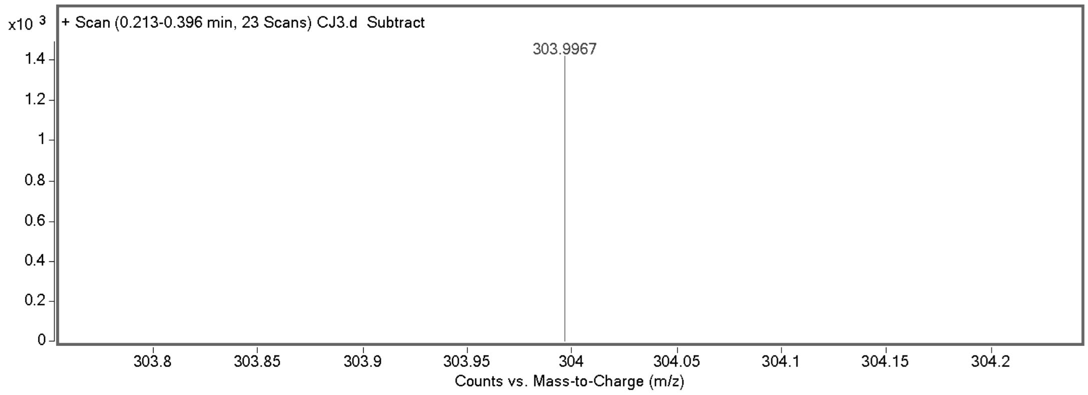

Figure S33. HRMS spectra of **2C-Br**.

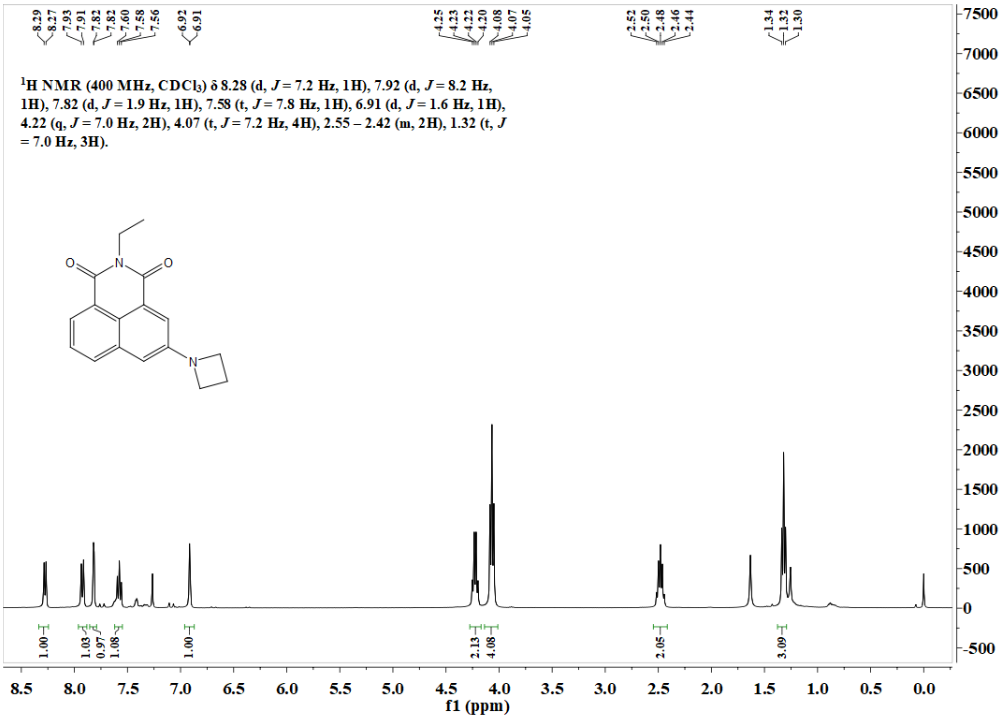

Figure S34. ^{1}H-NMR spectra of **Naph-2C** in $CDCl_3$.

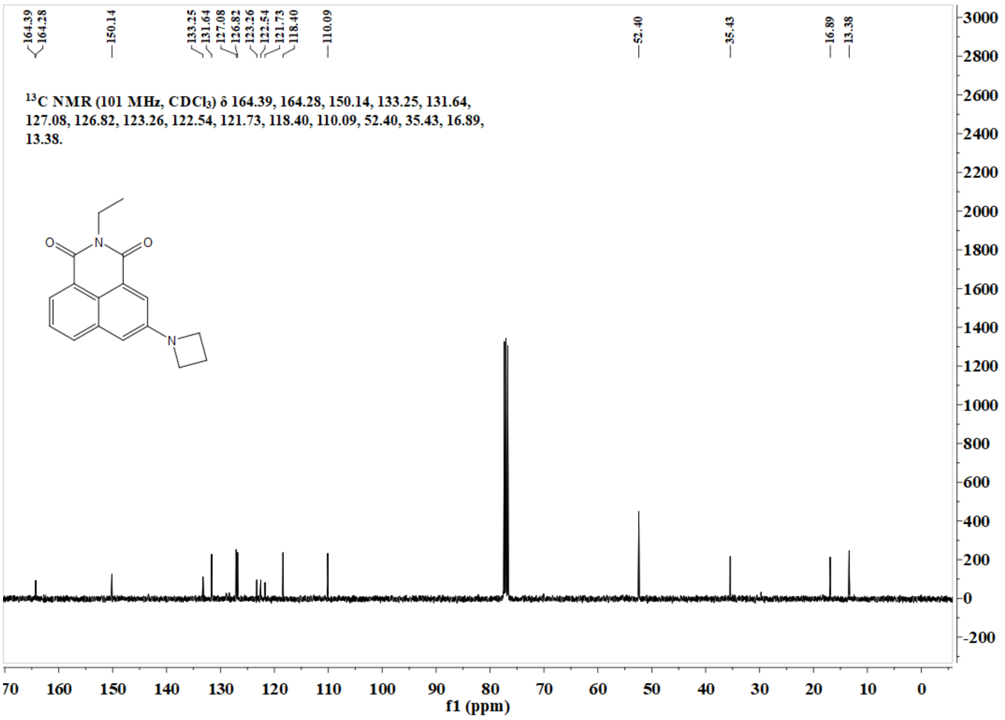

Figure S35. ^{13}C-NMR spectra of **Naph-2C** in $CDCl_3$.

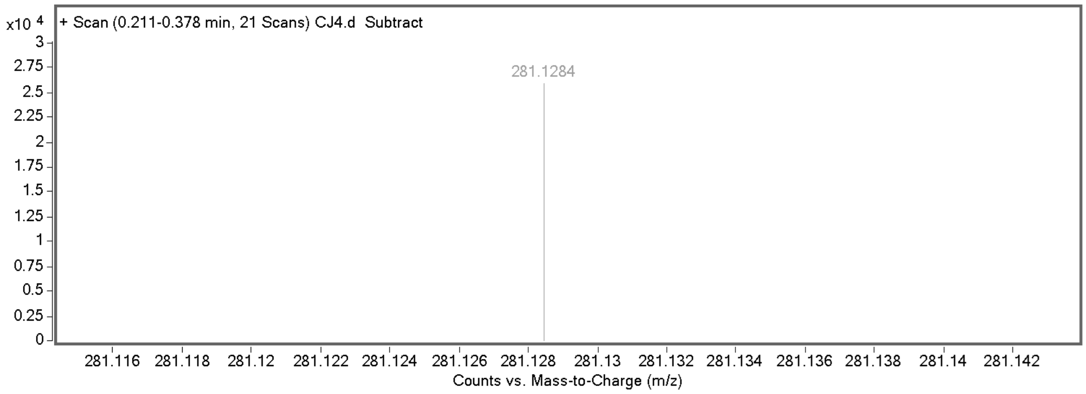

Figure S36. HRMS spectra of **Naph-2C**.

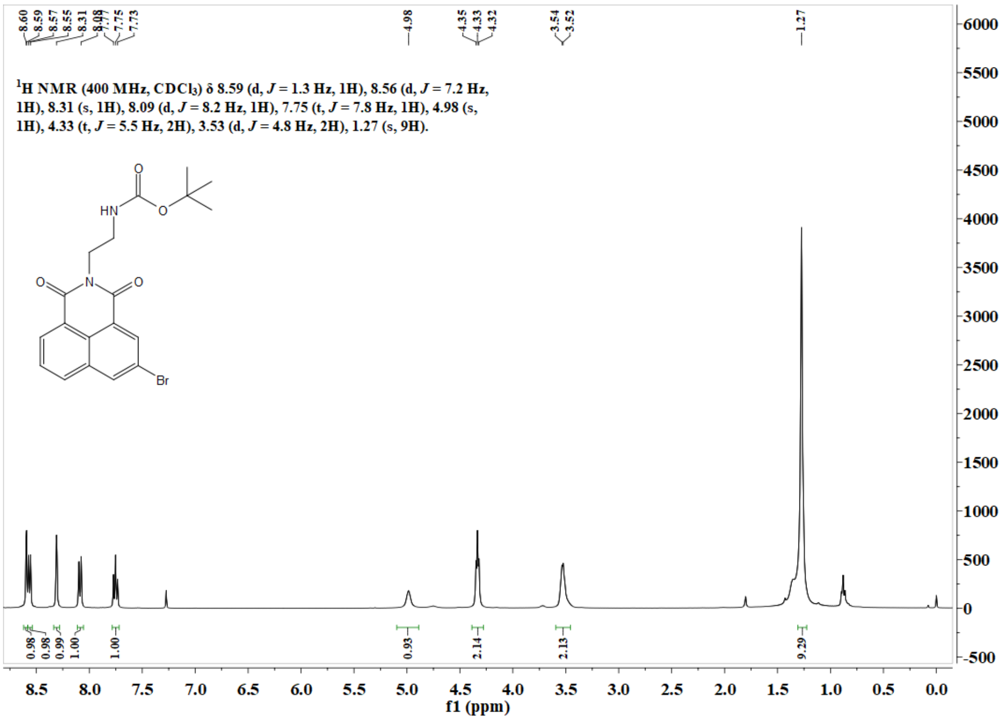

Figure S37. ^{1}H-NMR spectra of **2Boc-Br** in $CDCl_3$.

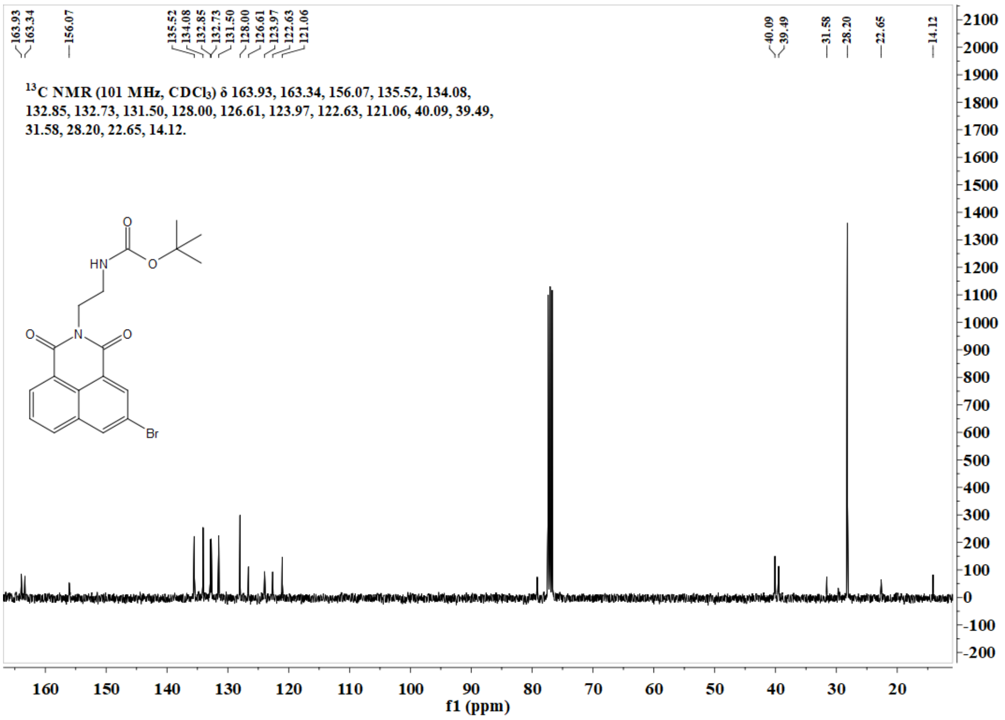

Figure S38. ^{13}C-NMR spectra of **2Boc-Br** in $CDCl_3$.

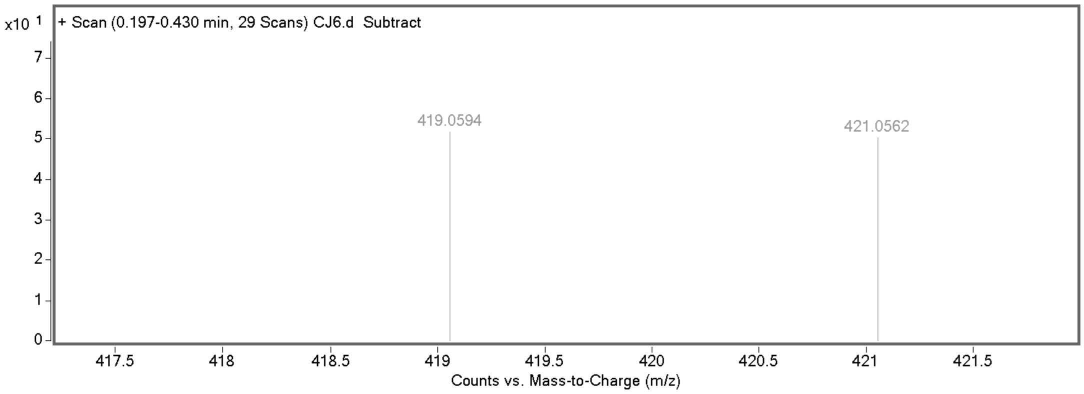

Figure S39. HRMS spectra of **2Boc-Br**.

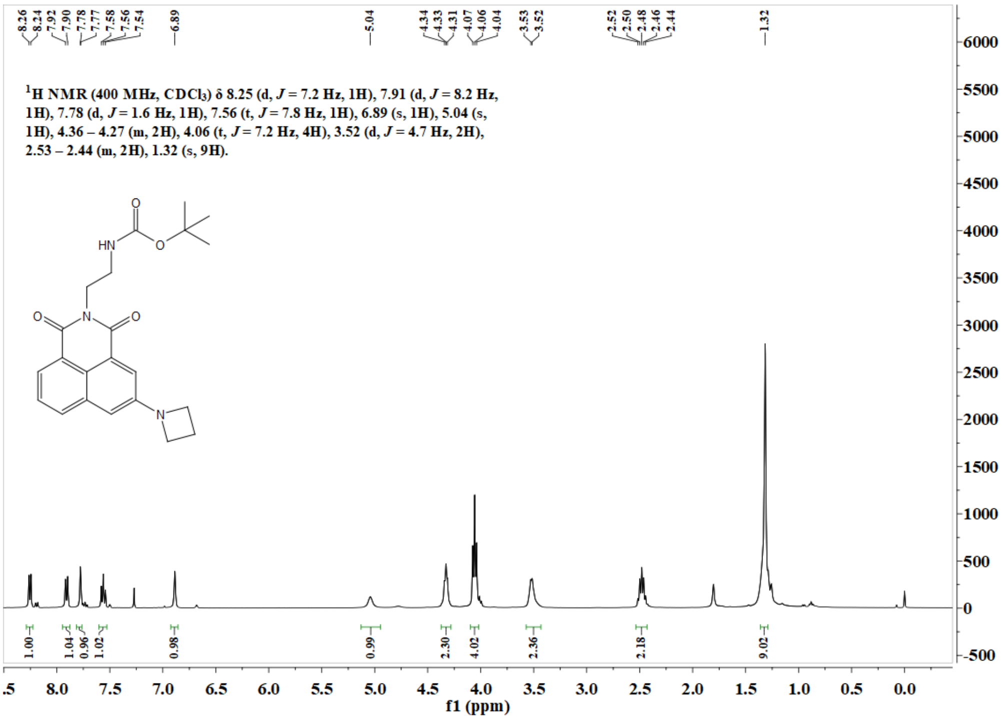

Figure S40. ^{1}H-NMR spectra of **Naph-2Boc** in $CDCl_3$.

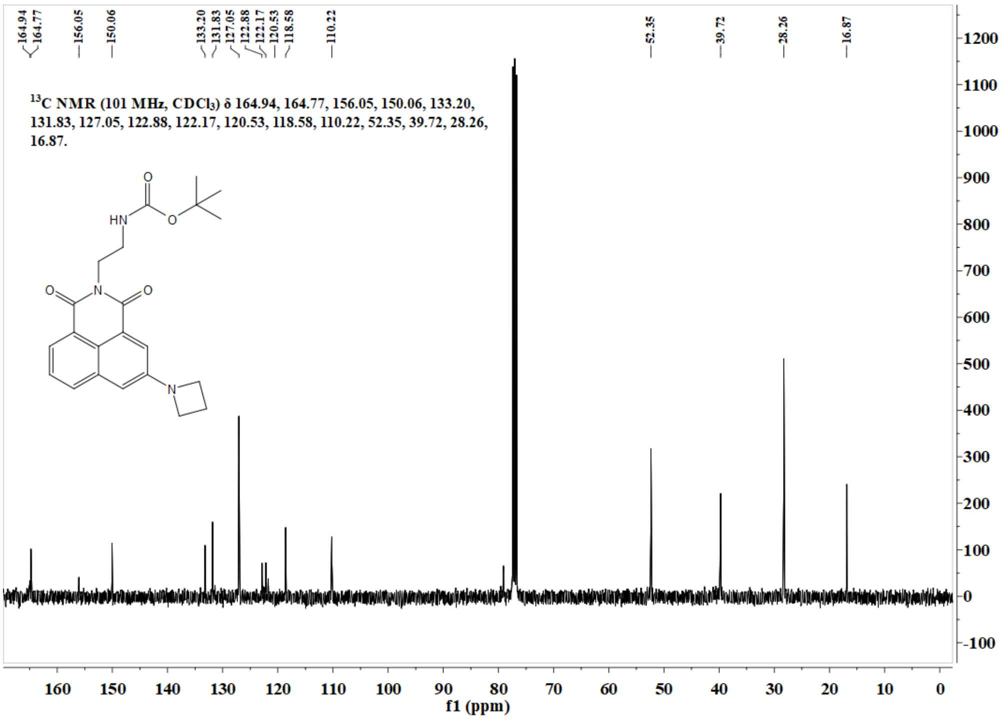

Figure S41. ^{13}C-NMR spectra of **Naph-2Boc** in $CDCl_3$.

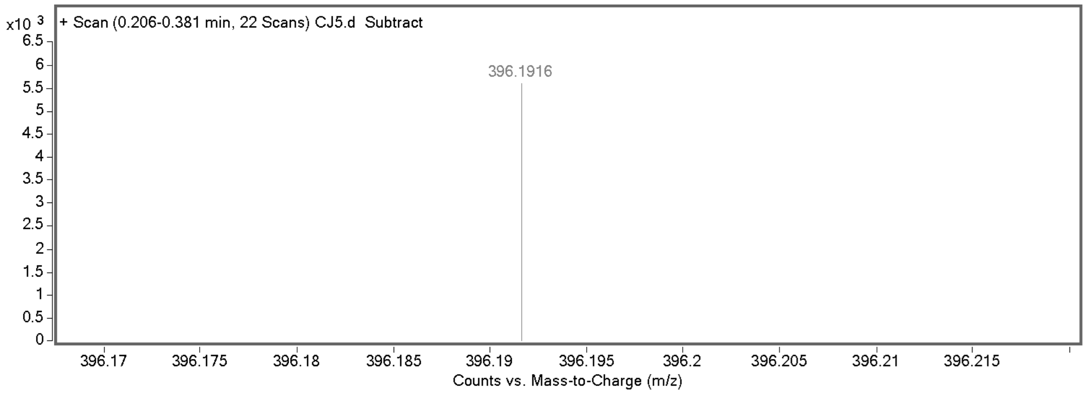

Figure S42. HRMS spectra of **Naph-2Boc**.

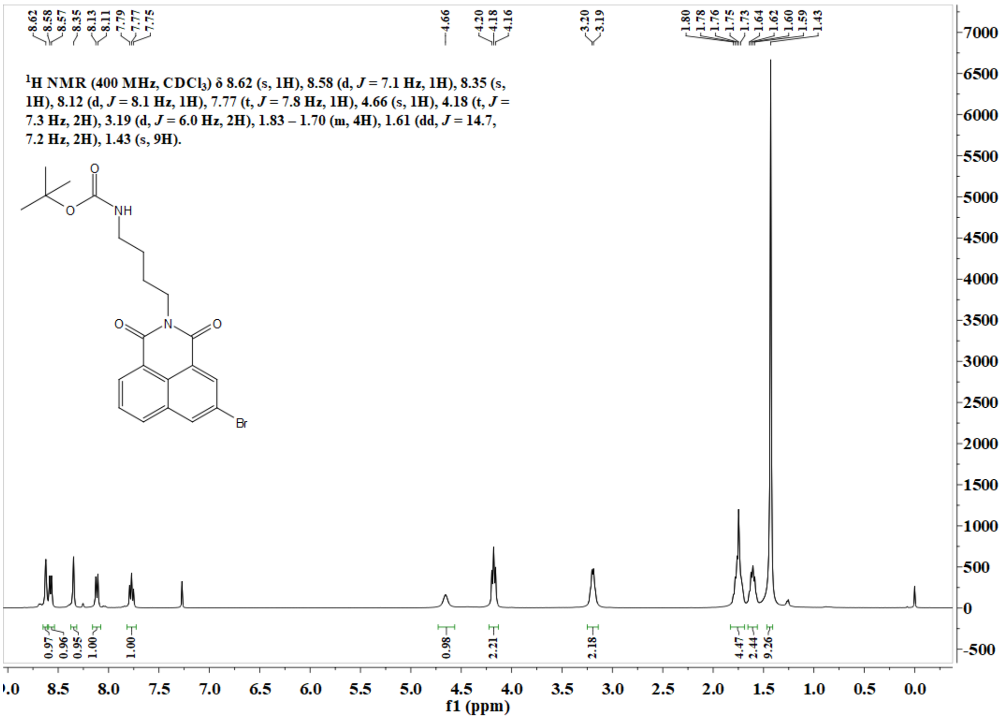

Figure S43. ^{1}H-NMR spectra of **4Boc-Br** in $CDCl_3$.

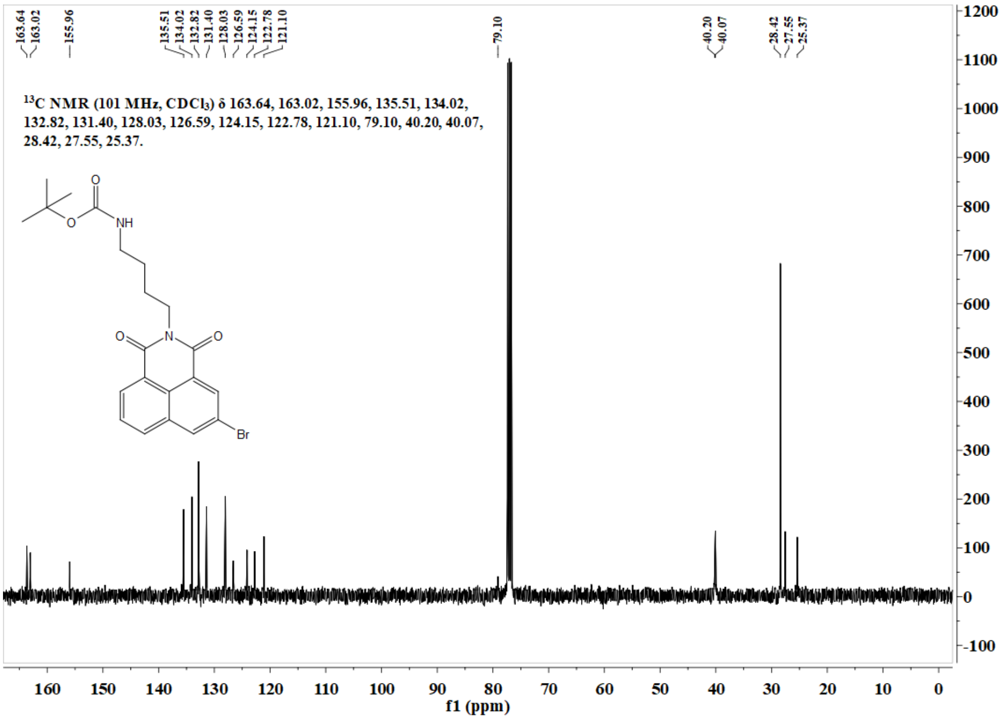

Figure S44. ^{13}C-NMR spectra of **4Boc-Br** in $CDCl_3$.

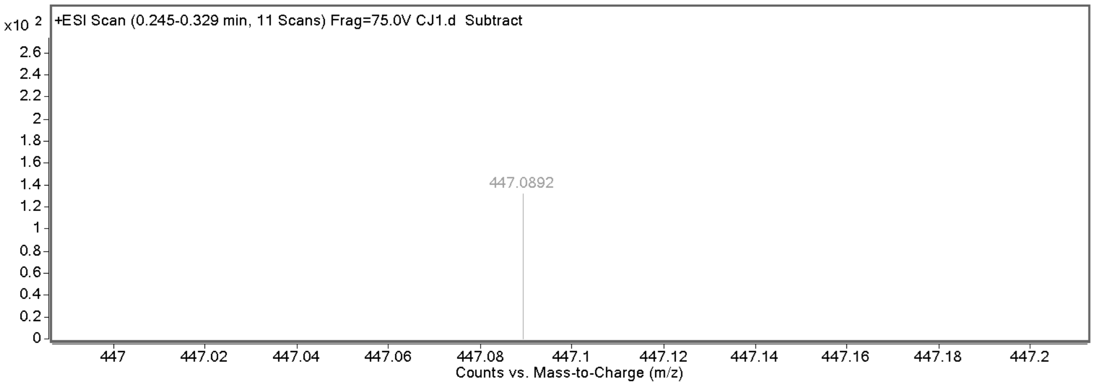

Figure S45. HRMS spectra of **4Boc-Br**.

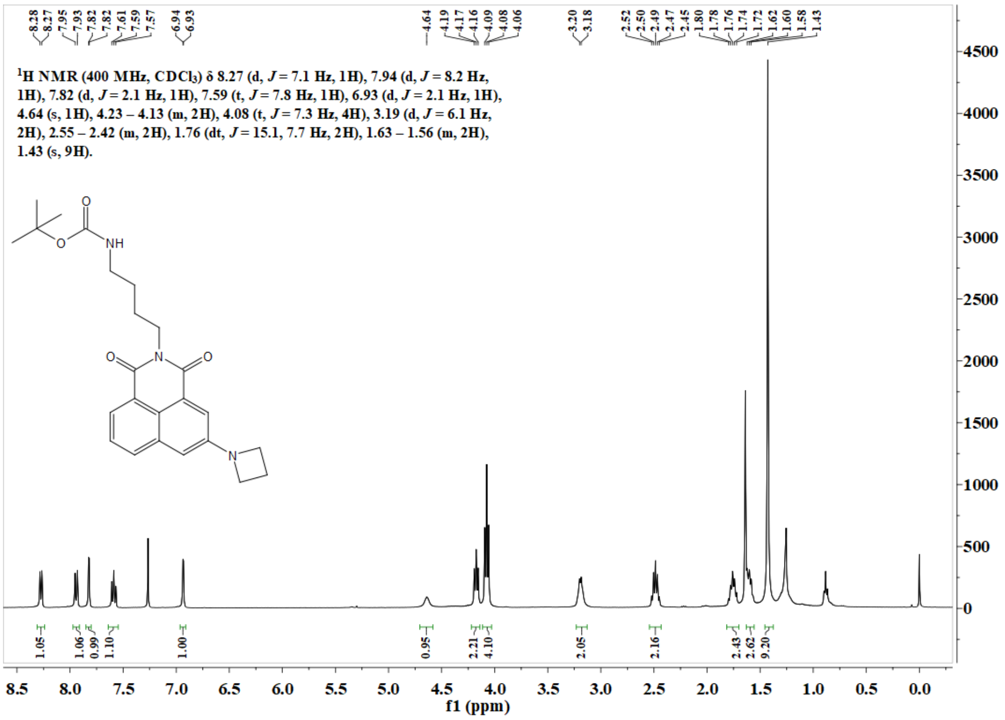

Figure S46. ^{1}H-NMR spectra of **Naph-4Boc** in $CDCl_3$.

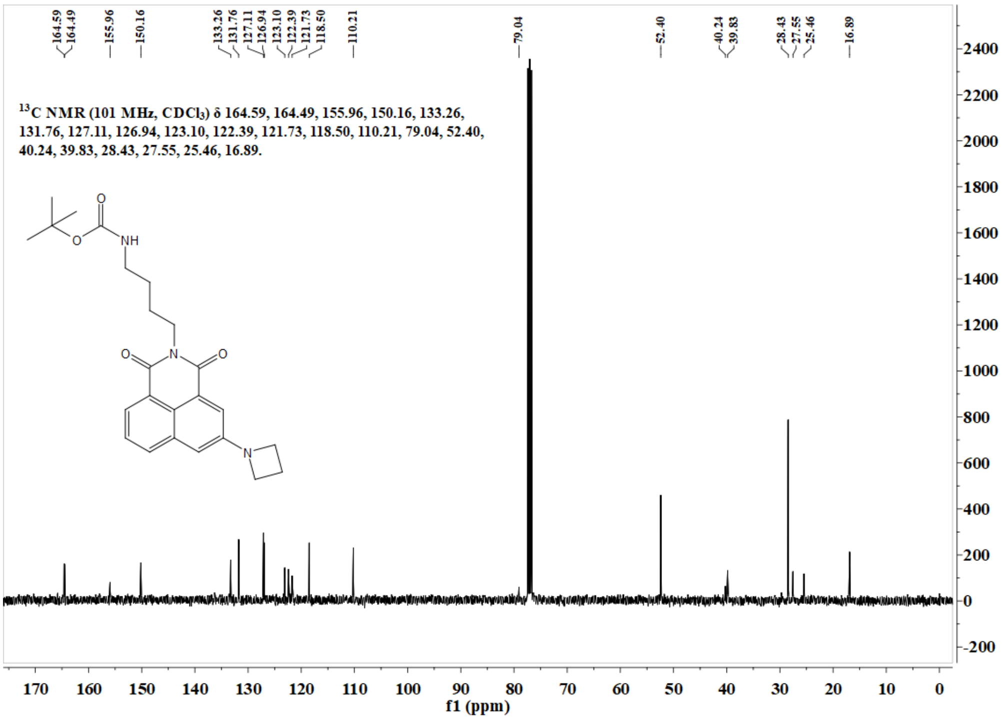

Figure S47. ^{13}C-NMR spectra of **Naph-4Boc** in $CDCl_3$.

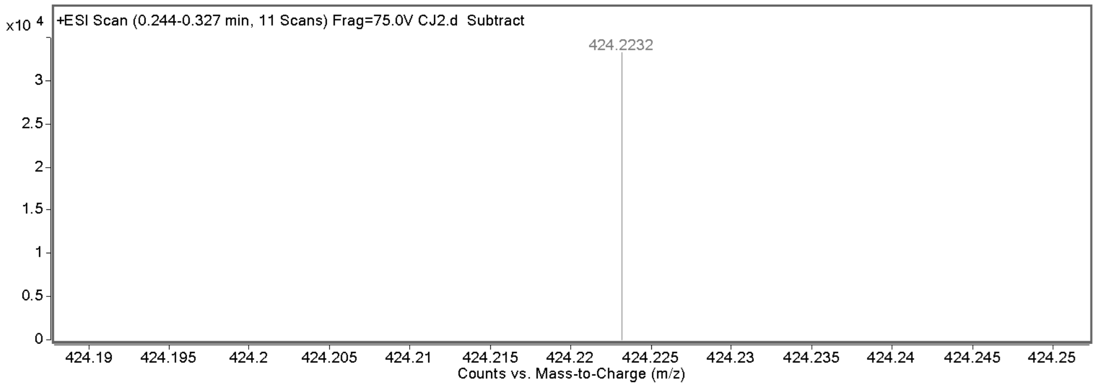

Figure S48. HRMS spectra of **Naph-4Boc**.

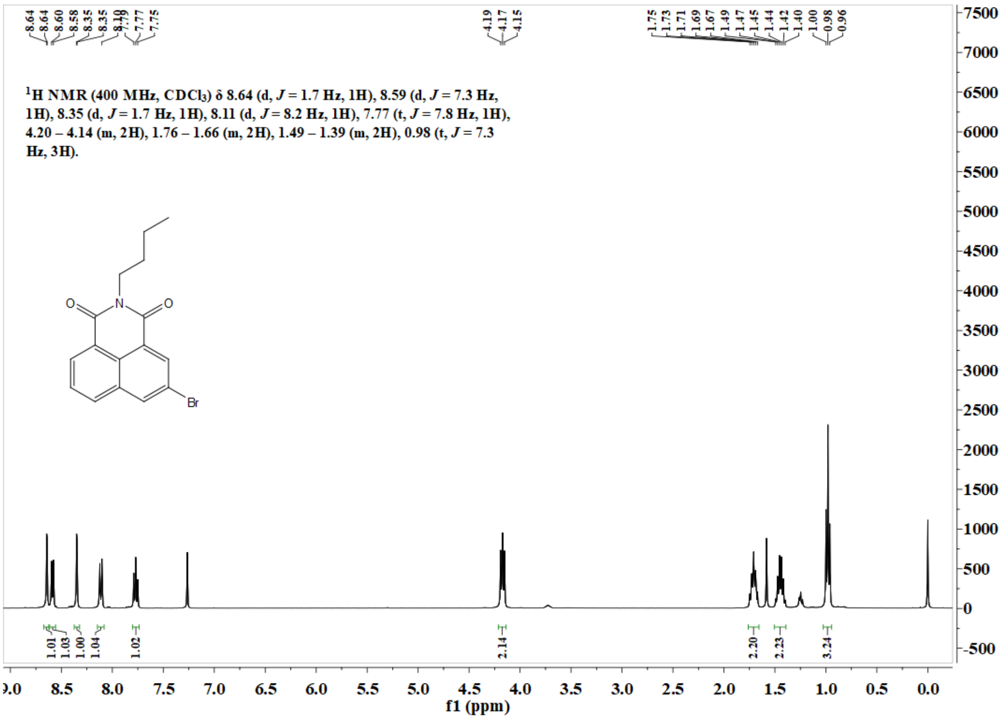

Figure S49. ^{1}H-NMR spectra of **4C-Br** in $CDCl_3$.

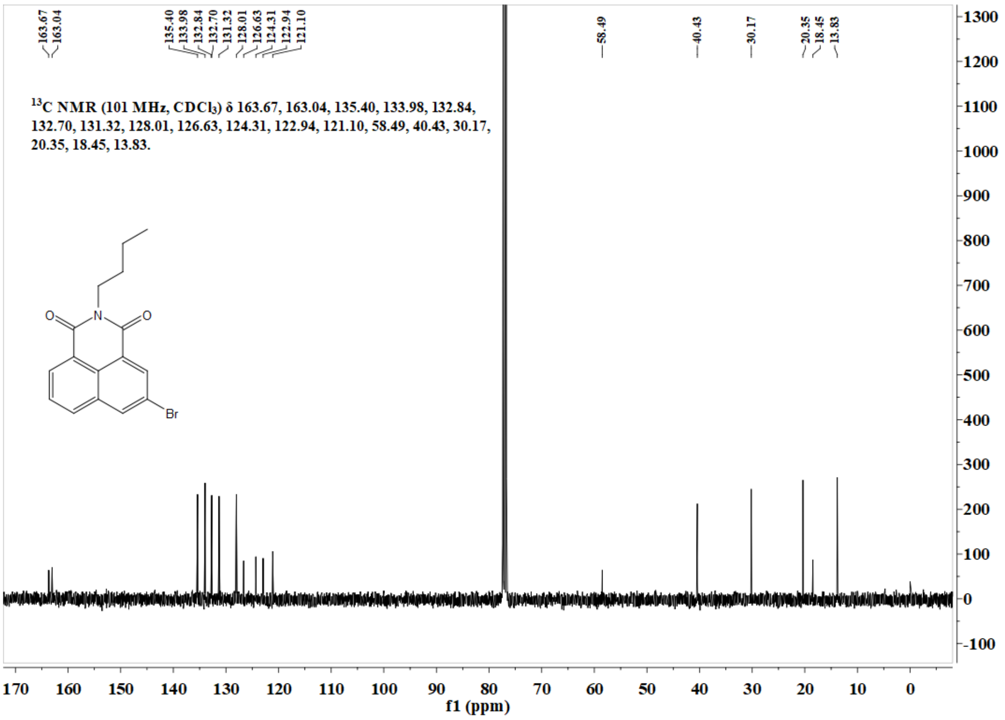

Figure S50. ^{13}C-NMR spectra of **4C-Br** in $CDCl_3$.

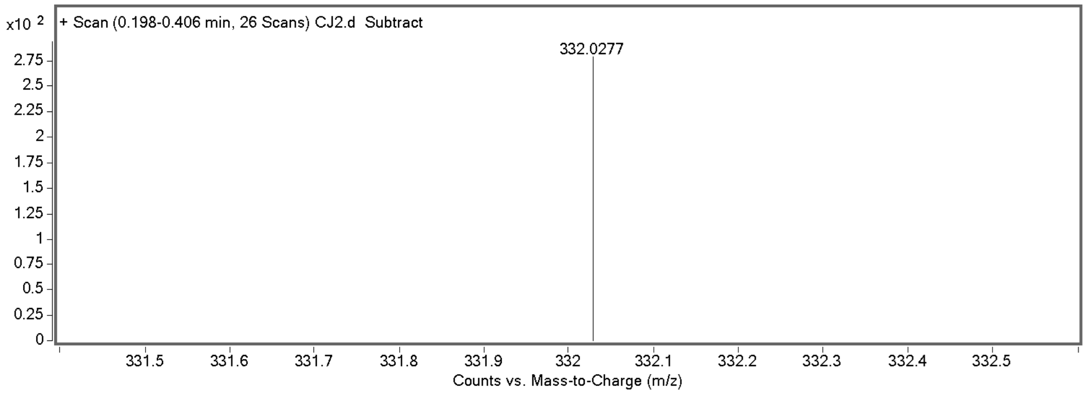

Figure S51. HRMS spectra of **4C-Br**.

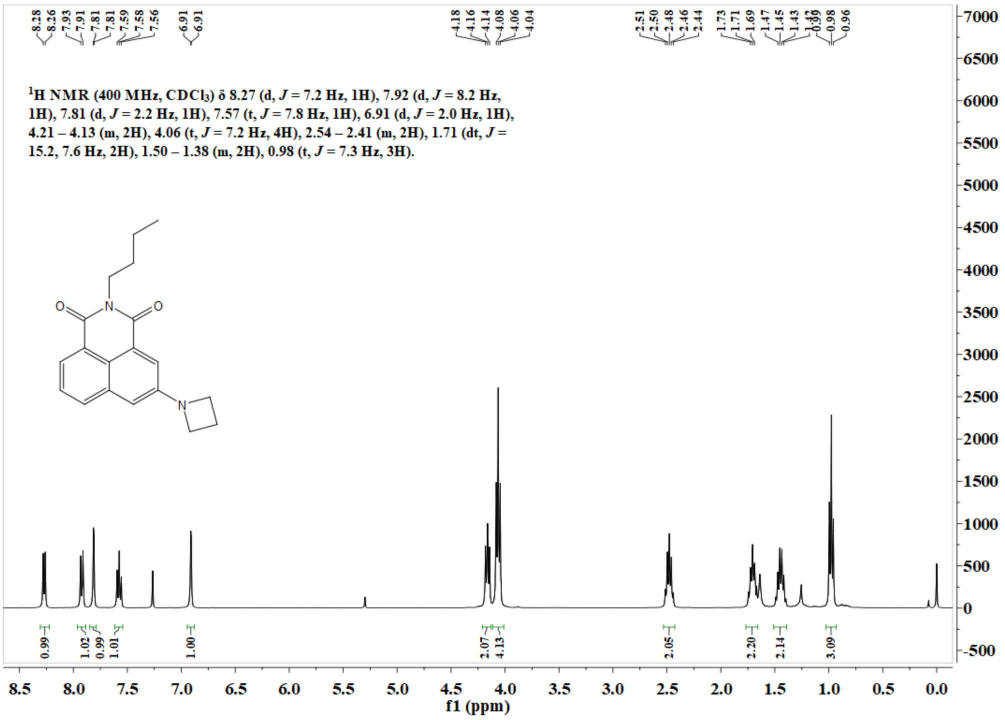

Figure S52. ¹H-NMR spectra of **Naph-4C** in $CDCl_3$.

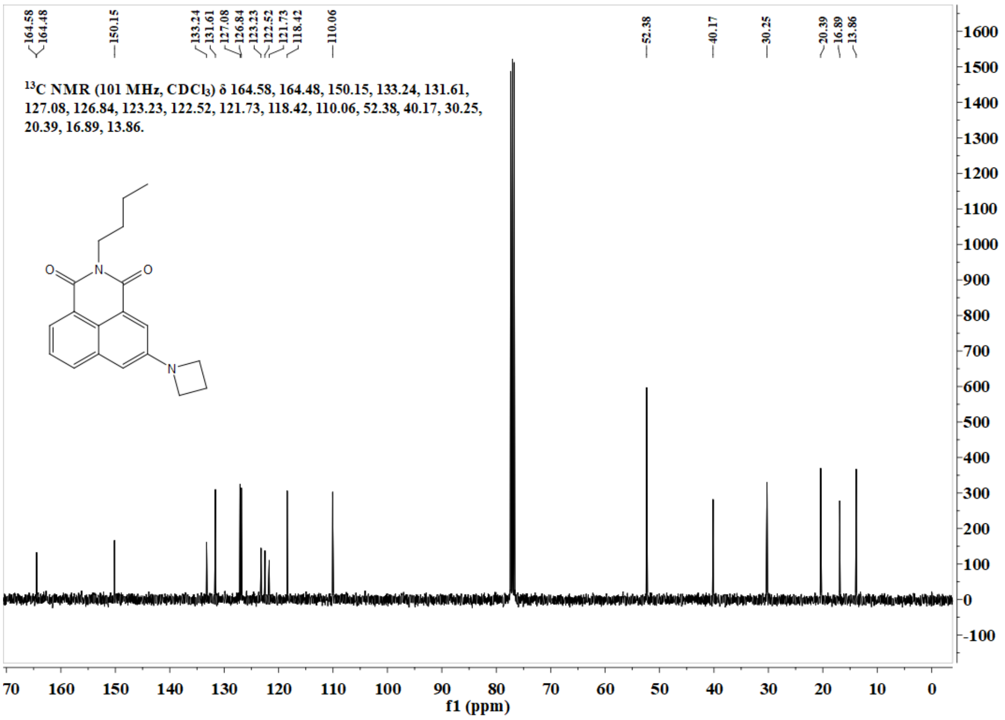

Figure S53. ¹³C-NMR spectra of **Naph-4C** in $CDCl_3$.

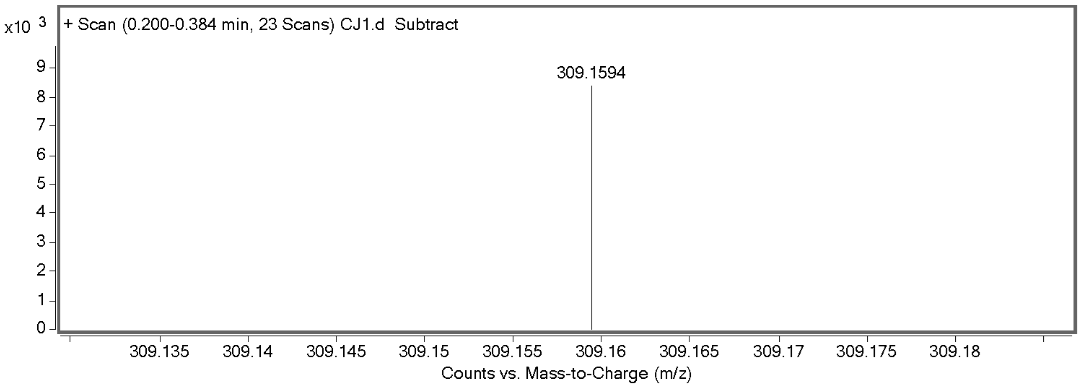

Figure S54. HRMS spectra of **Naph-4C**.

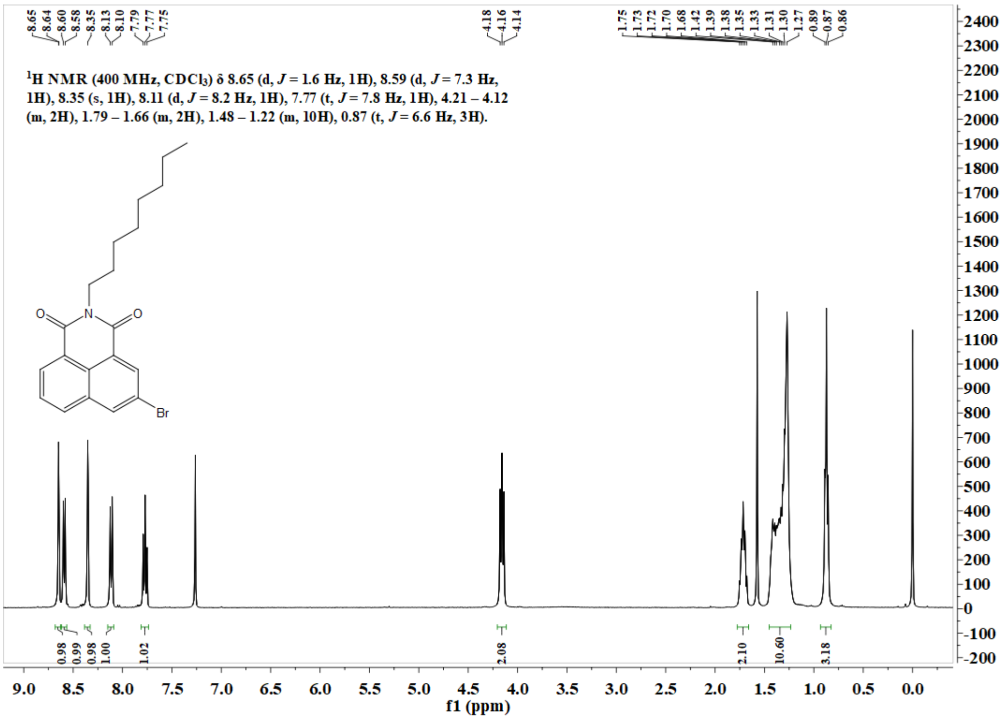

Figure S55. ^{1}H-NMR spectra of **8C-Br** in CDCl$_3$.

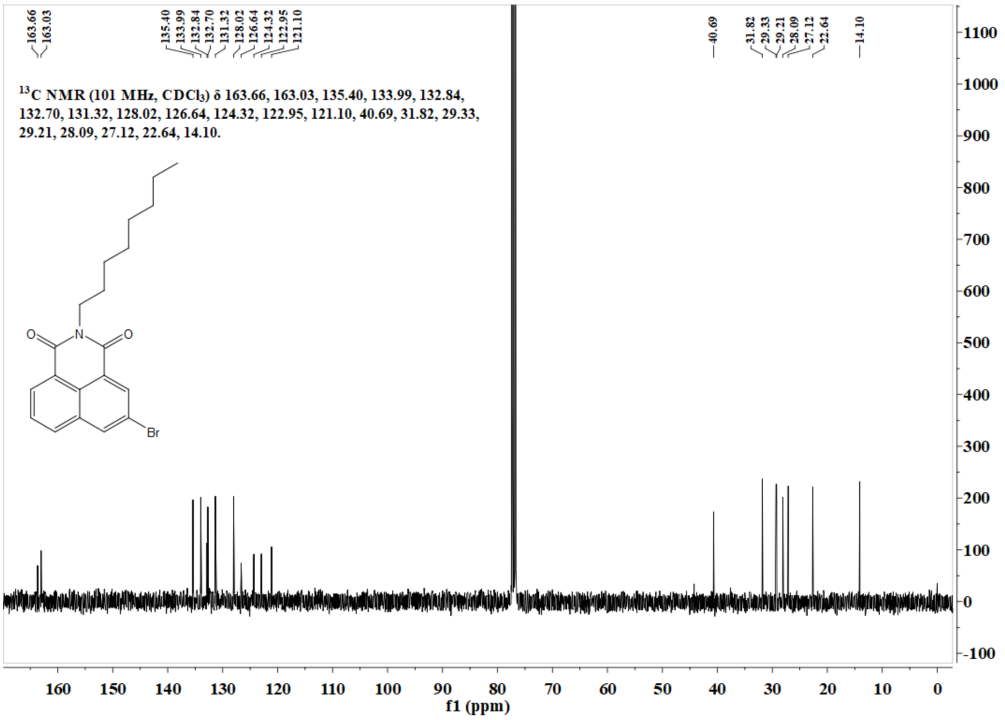

Figure S56. ^{13}C-NMR spectra of **8C-Br** in $CDCl_3$.

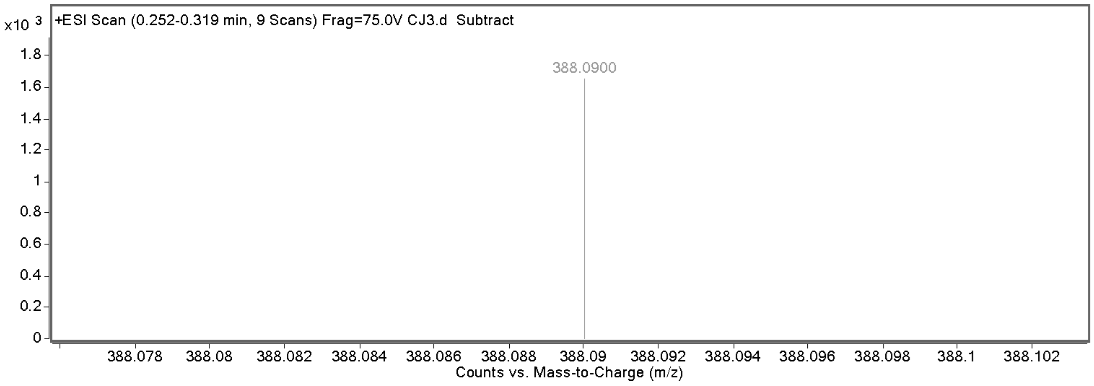

Figure S57. HRMS spectra of **8C-Br**.

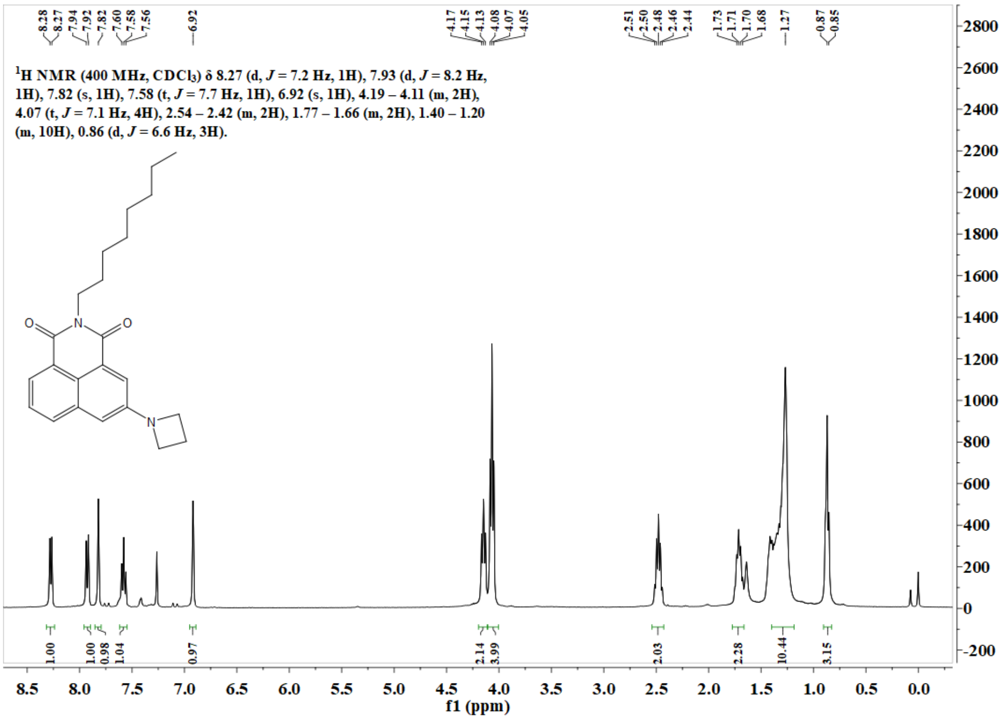

Figure S58. ^{1}H-NMR spectra of **Naph-8C** in CDCl$_3$.

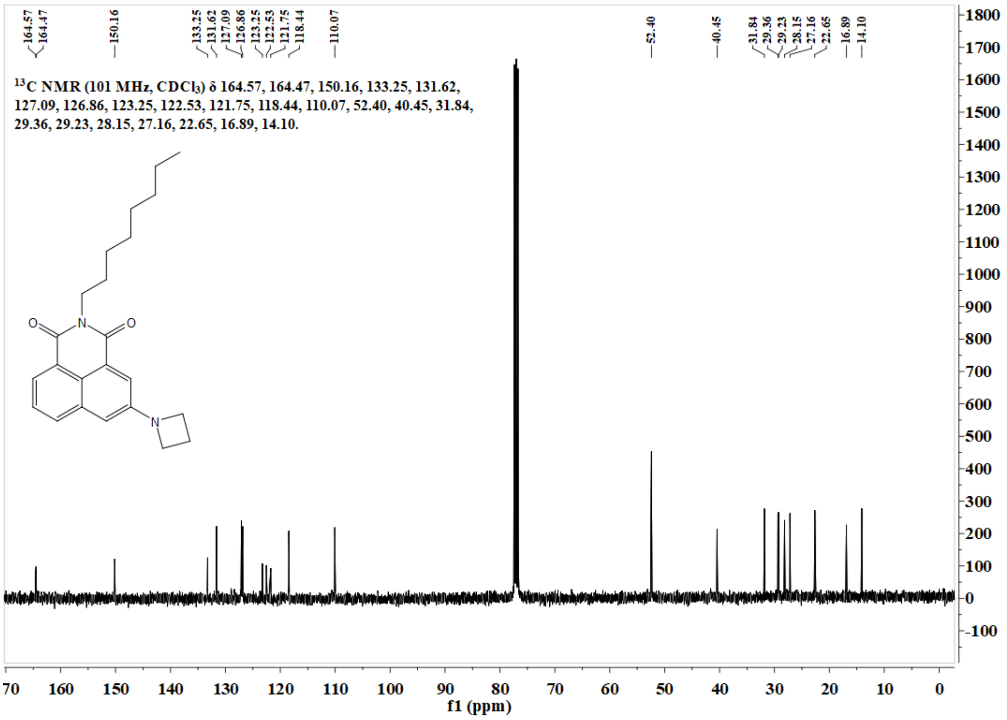

Figure S59. ^{13}C-NMR spectra of **Naph-8C** in CDCl$_3$.

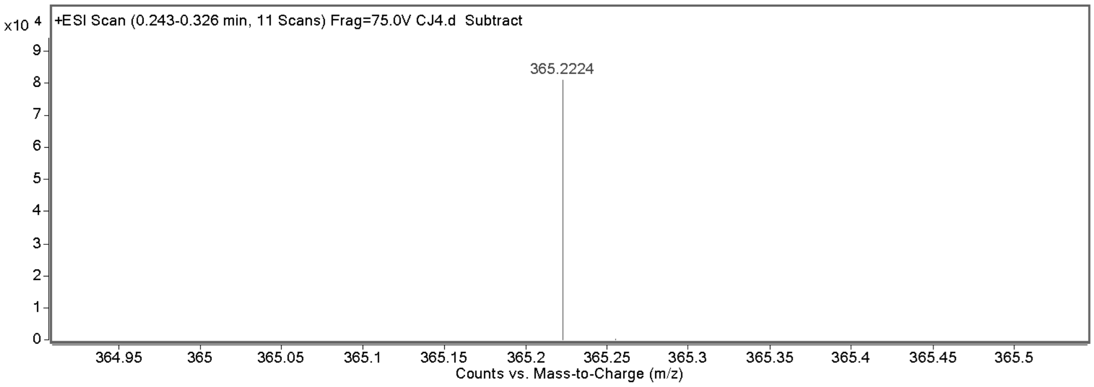

Figure S60. HRMS spectra of **Naph-8C**.

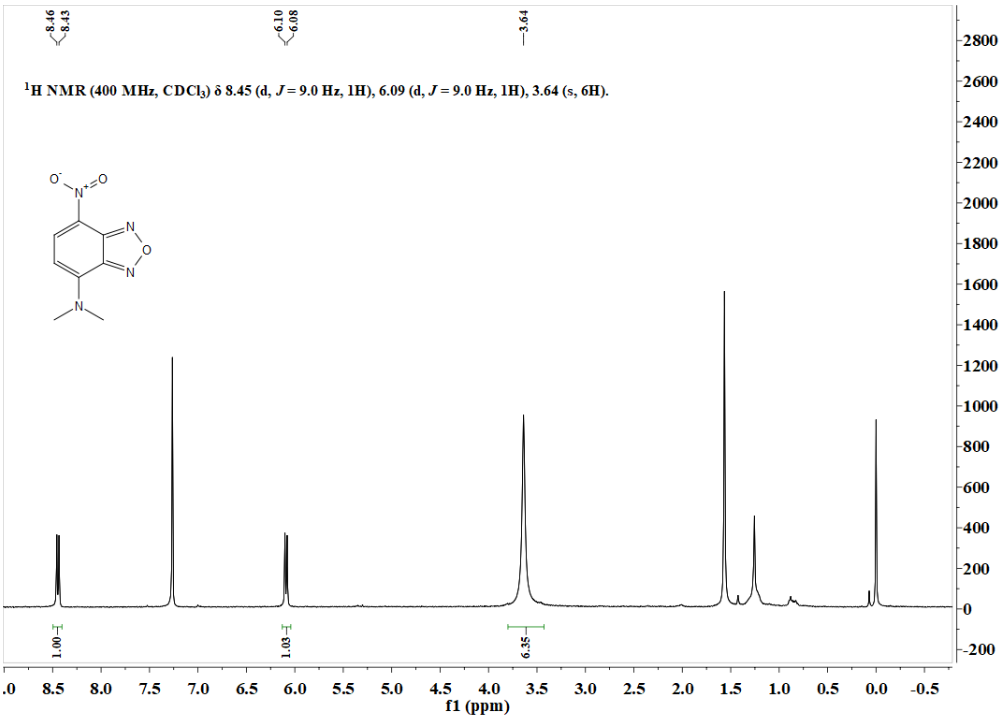

Figure S61. ^{1}H-NMR spectra of **NBD-DMA** in $CDCl_3$.

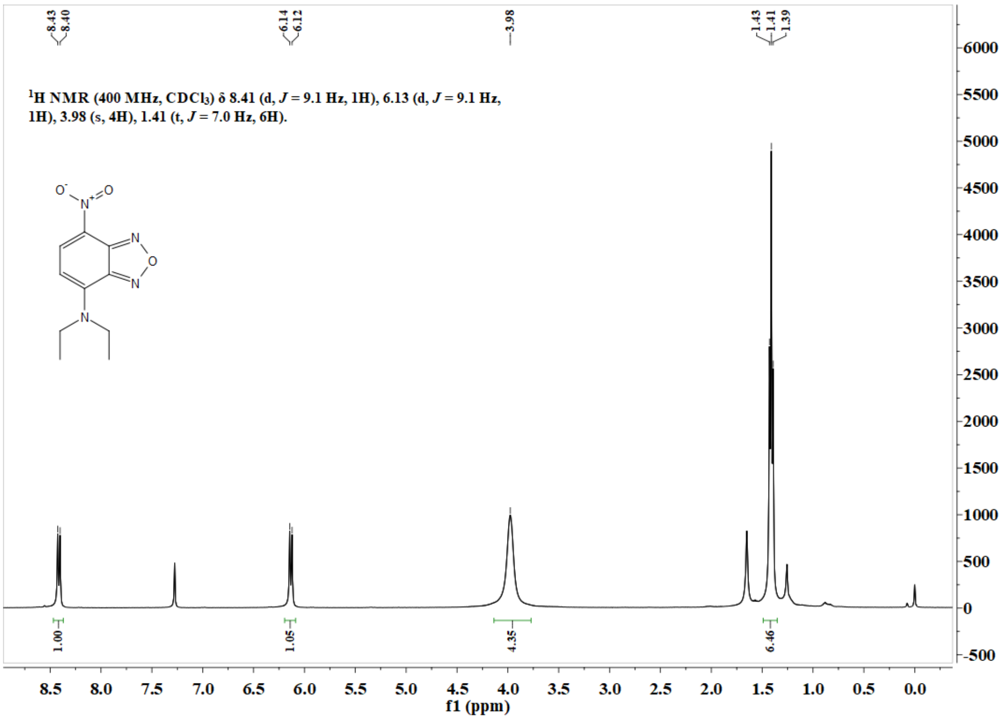

Figure S62. ^{1}H-NMR spectra of **NBD-DEA** in $CDCl_3$.

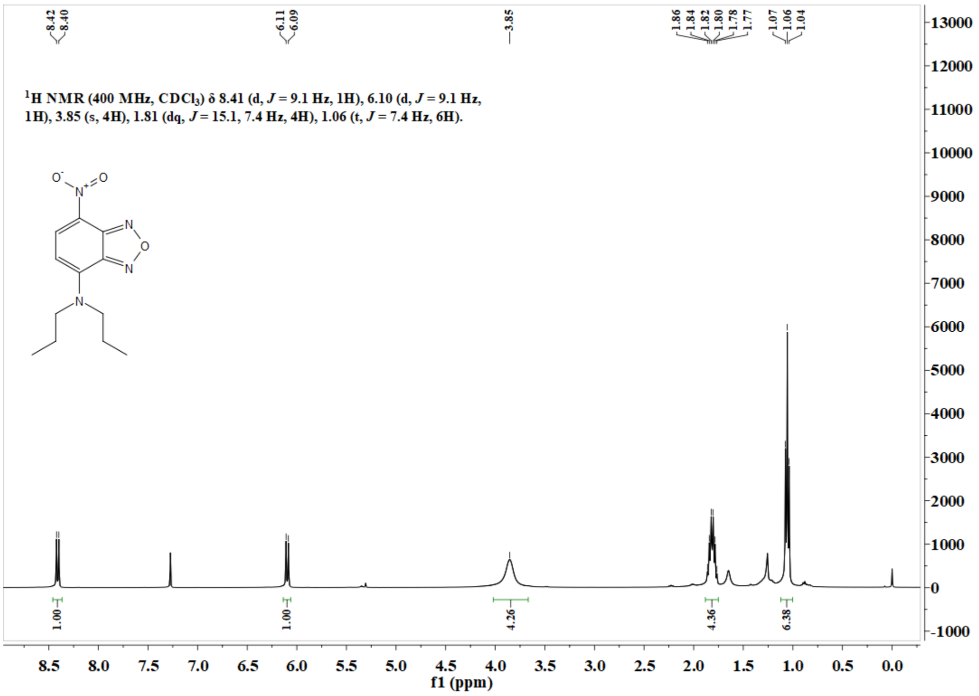

Figure S63. ^{1}H-NMR spectra of **LD-BFP488** in $CDCl_3$.

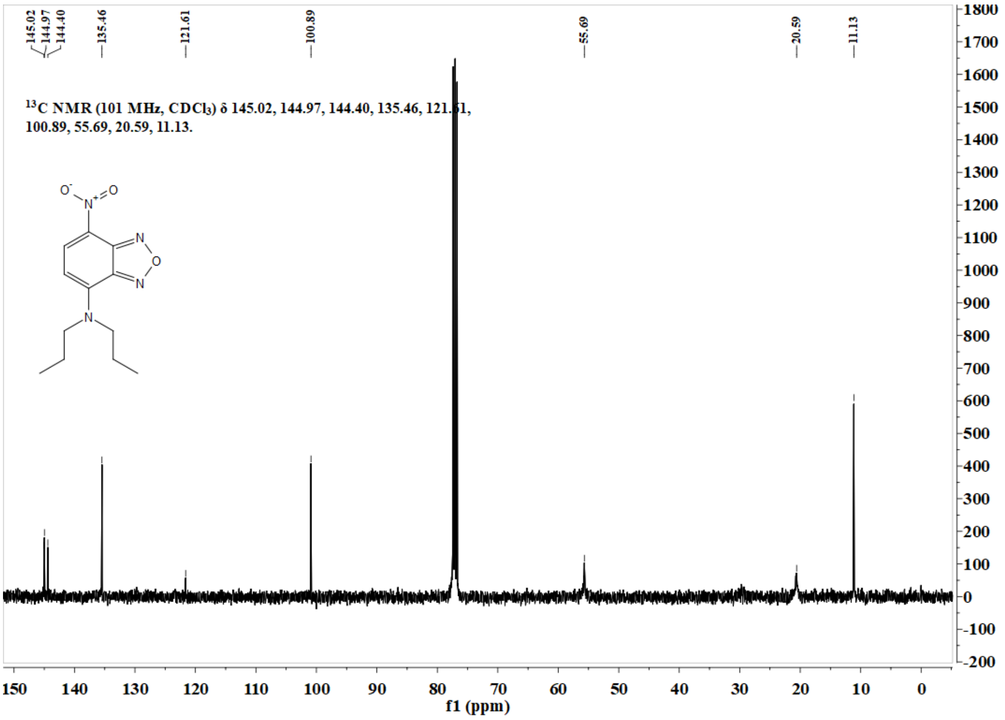

Figure S64. ^{13}C-NMR spectra of **LD-BFP488** in $CDCl_3$.

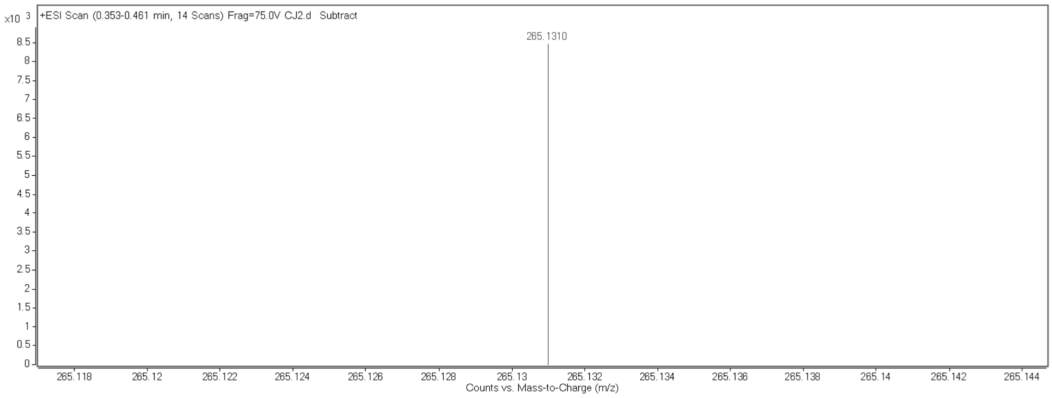

Figure S65. HRMS spectra of **LD-BFP488**.

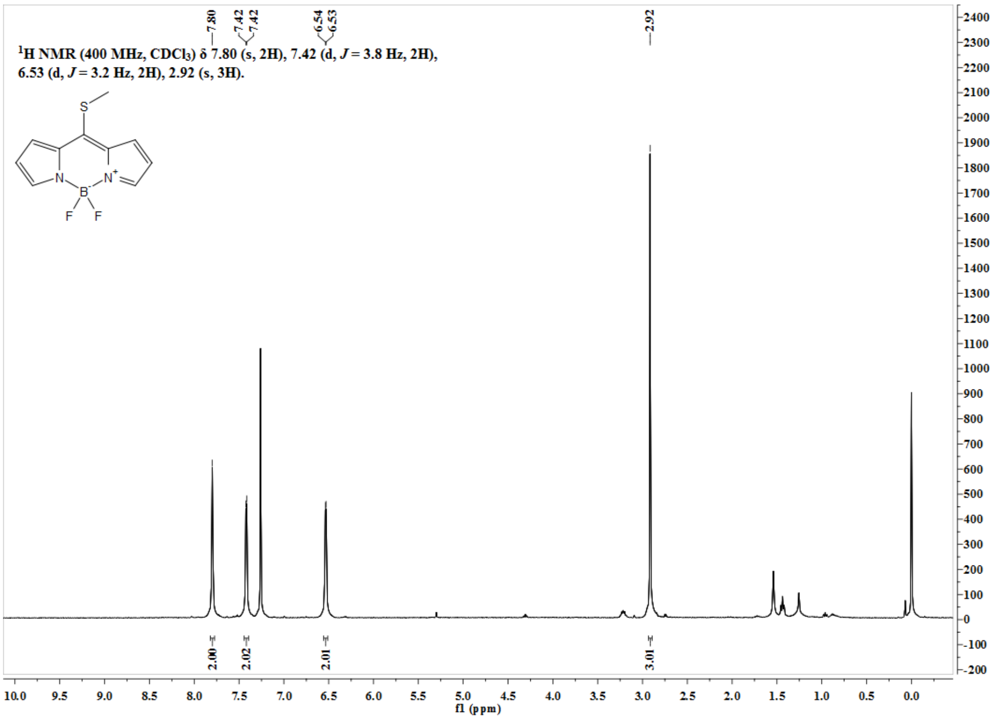

Figure S66. ^{1}H-NMR spectra of **BDP-MeS** in $CDCl_3$.

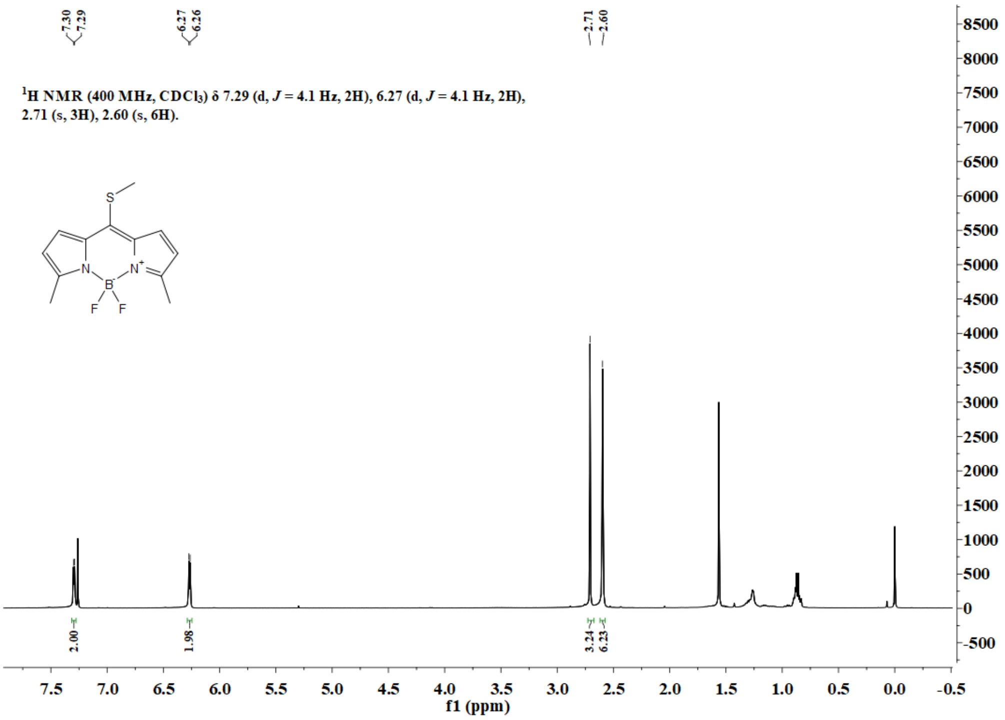

Figure S67. ^{1}H-NMR spectra of **BDP-2MeS** in $CDCl_3$.

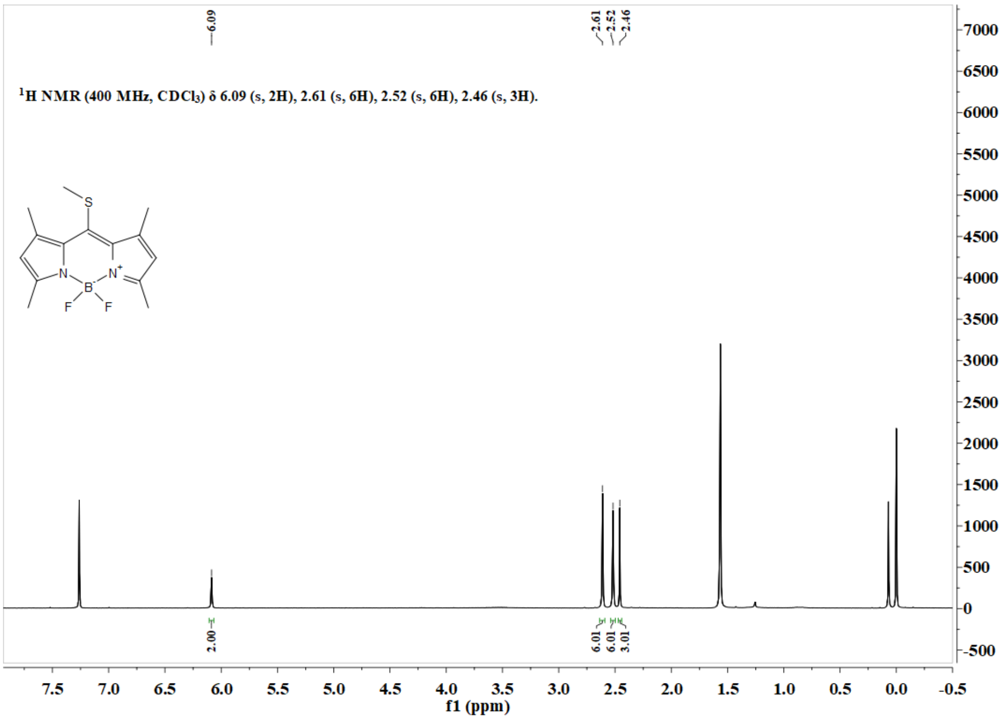

Figure S68. ^{1}H-NMR spectra of **BDP-4MeS** in $CDCl_3$.

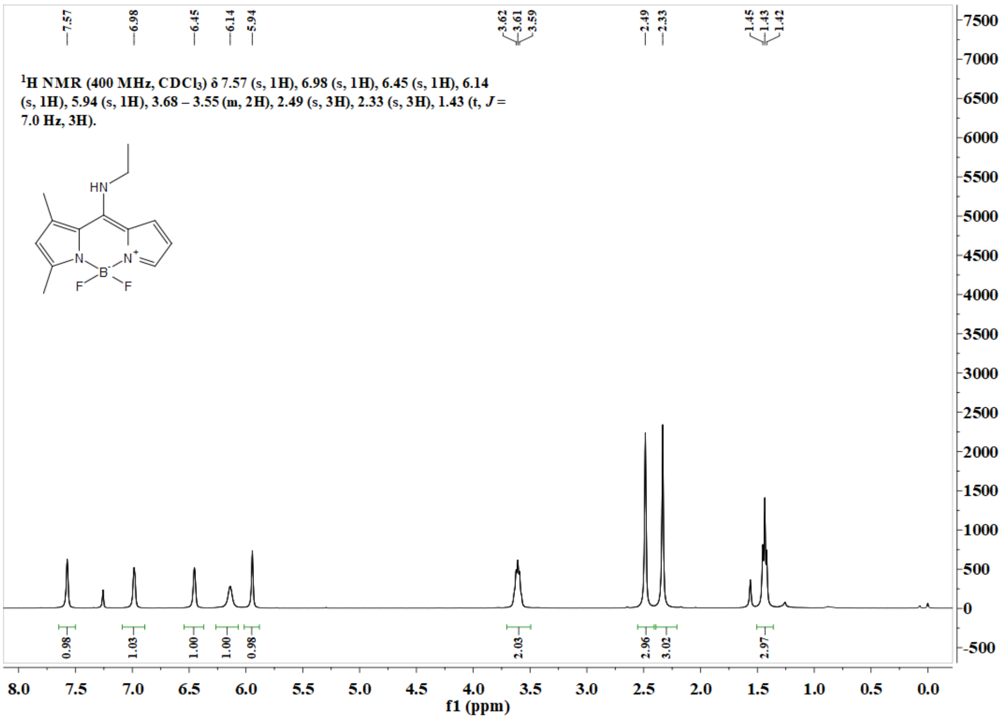

Figure S69. ^{1}H-NMR spectra of **LD-BFP405** in $CDCl_3$.

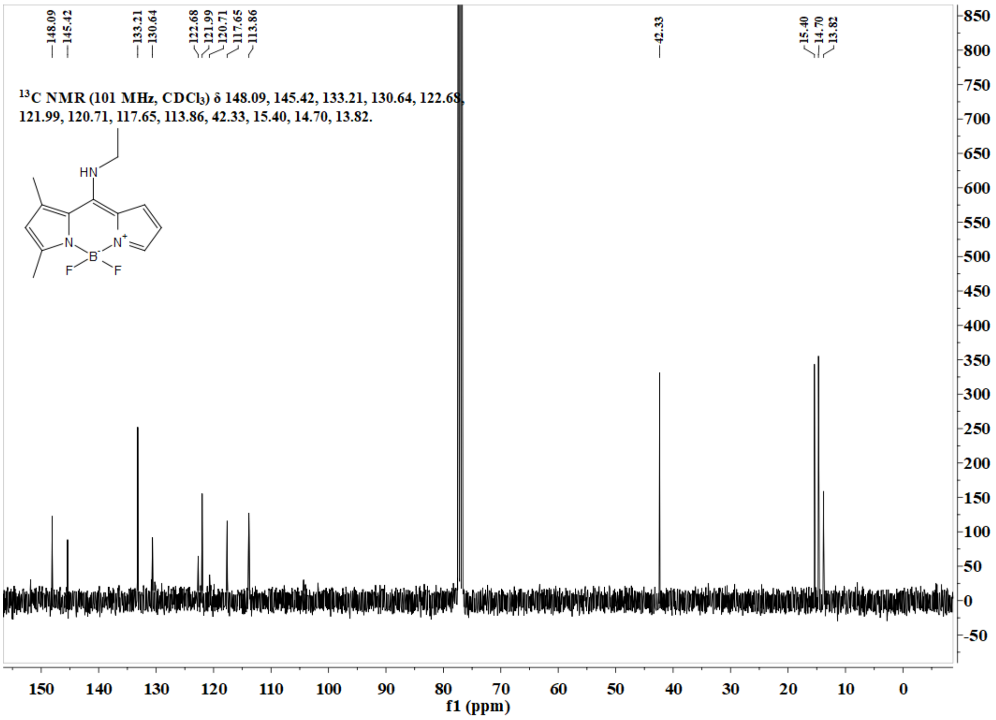

Figure S70. ^{13}C-NMR spectra of **LD-BFP405** in $CDCl_3$.

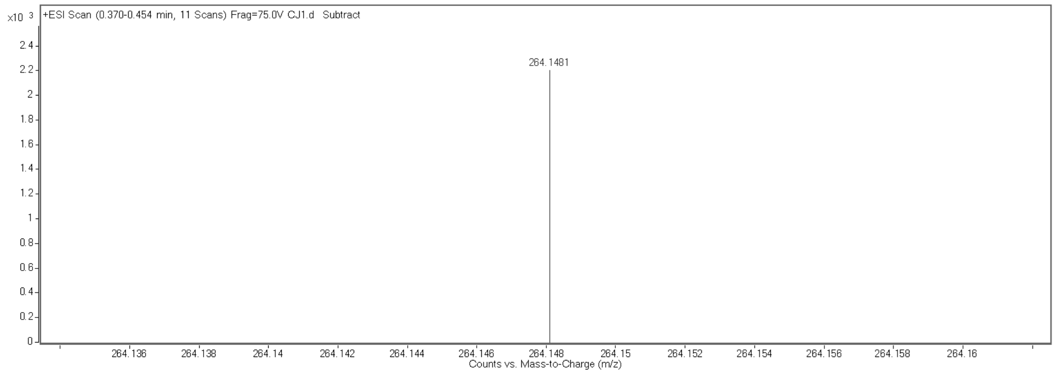

Figure S71. HRMS spectra of **LD-BFP405**.

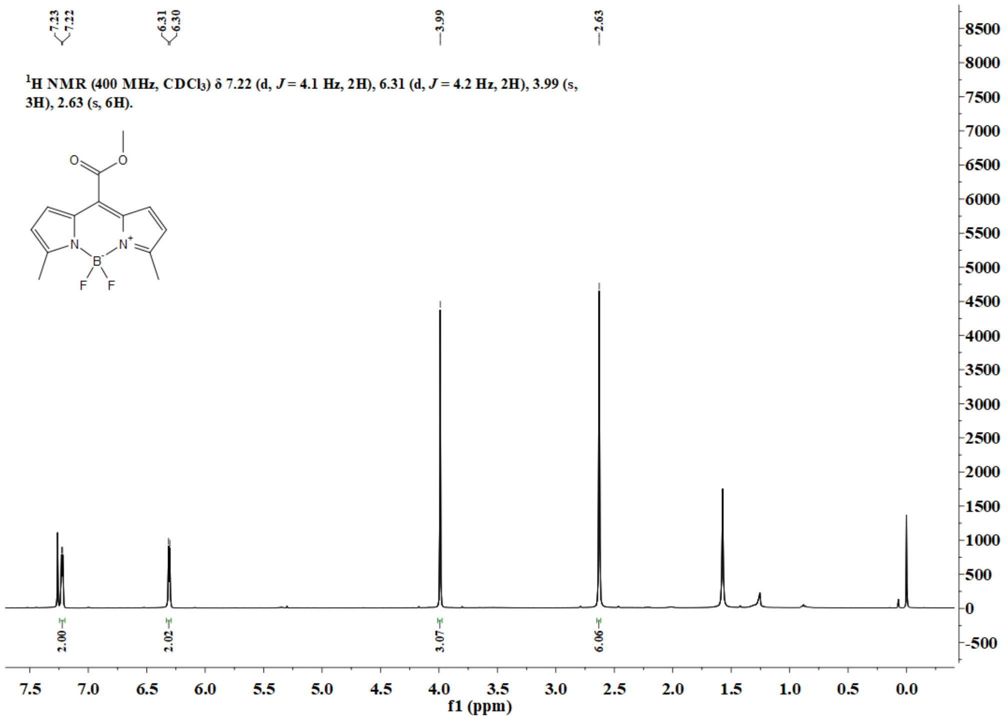

Figure S72. ^{1}H-NMR spectra of **LD-BFP543** in $CDCl_3$.

8. Reference

[1] Z. Ye, W. Yang, C. Wang, Y. Zheng, W. Chi, X. Liu, Z. Huang, X. Li, Y. Xiao, *J. Am. Chem. Soc.* **2019**, *141*, 14491.

[2] J. Pinte, C. Joly, K. Plé, P. Dole, A. Feigenbaum, *J. Agric. Food Chem.***2008**, *56*, 10003.

[3] W. Chi, Q. Qiao, R. Lee, W. Liu, Y. S. Teo, D. Gu, M. J. Lang, Y.-T. Chang, Z. Xu, X. Liu, *Angew. Chem. Int. Ed.* **2019**, *58*, 7073.

[4] T. V. Goud, A. Tutar, J.-F. Biellmann, *Tetrahedron* **2006**, *62*, 5084.

[5] X. Peng, Y. liu, Q. Shen, D. Chen, X. Chen, Y. Fu, J. Wang, X. Zhang, H. Jiang, J. Li, *J. Org. Chem.* **2022**, *87*, 11958.

[6] Z. Tian, L. Ding, K. Li, Y. Song, T. Dou, J. Hou, X. Tian, L. Feng, G. Ge, J. Cui, *Anal. Chem.* **2019**, *91*, 5638.
